# Towards the engineering of a photon-only two-stroke rotary molecular motor

**DOI:** 10.1038/s41467-022-33695-x

**Published:** 2022-10-28

**Authors:** Michael Filatov(Gulak), Marco Paolino, Robin Pierron, Andrea Cappelli, Gianluca Giorgi, Jérémie Léonard, Miquel Huix-Rotllant, Nicolas Ferré, Xuchun Yang, Danil Kaliakin, Alejandro Blanco-González, Massimo Olivucci

**Affiliations:** 1 Department of Chemistry, Kyungpook National University, Daegu, 702-701 South Korea; 2 Dipartimento di Biotecnologie, Chimica e Farmacia, Università di Siena, Via A. Moro 2, 53100 Siena, Italy; 3 Institut de Physique et Chimie des Matériaux de Strasbourg, Université de Strasbourg, CNRS UMR 7504, Strasbourg, France; 4 Institut de Chimie Radicalaire (UMR-7273), Aix-Marseille Université, CNRS, 13397 Marseille Cedex 20, France; 5 Chemistry Department, Bowling Green State University, Overmann Hall, Bowling Green, OH 43403 USA

**Keywords:** Light harvesting, Atomistic models, Excited states, Energy transfer

## Abstract

The rational engineering of photoresponsive materials, e.g., light-driven molecular motors, is a challenging task. Here, we use structure-related design rules to prepare a prototype molecular rotary motor capable of completing an entire revolution using, exclusively, the sequential absorption of two photons; i.e., a photon-only two-stroke motor. The mechanism of rotation is then characterised using a combination of non-adiabatic dynamics simulations and transient absorption spectroscopy measurements. The results show that the rotor moiety rotates axially relative to the stator and produces, within a few picoseconds at ambient T, an intermediate with the same helicity as the starting structure. We discuss how such properties, that include a 0.25 quantum efficiency, can help overcome the operational limitations of the classical overcrowded alkene designs.

## Abstract (web summary)

Improving the efficiency of light-driven molecular rotary motors is a challenging task. Here, the authors combine theoretical modeling, synthesis and spectroscopy to prepare a prototype molecular motor capable of avoiding inefficient thermally activated motion; thus offering prospects to implement a 2-stroke photon-only molecular motor.

## Introduction

The function of light-driven molecular motors is to generate unidirectional and cyclic motion, e.g., recursive translation, rotation or more complex cyclic displacements, using photons as the energy source^1–7^. The classical design of their rotary version is based on the concept of overcrowded alkenes^8–13^, where two moieties (rotor or rotator and stator) are connected by a double bond (the axle) and experience steric repulsion resulting in a helical shape (P or M helicity).

**Fig. 1:**
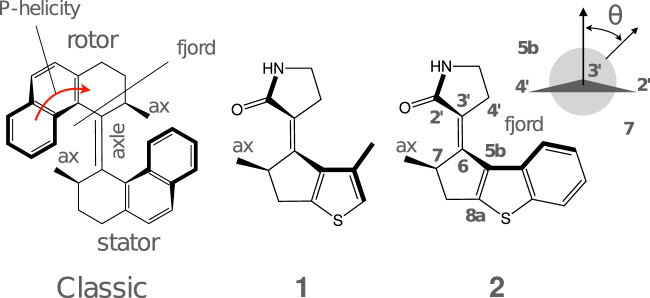
Chemical formulae of the classic light-driven rotary motor (LDRM) and the LDRMs studied here. The red arrow shows the direction, in which rotation of the upper part occurs with respect to the lower part of the motor.

**Fig. 2:**
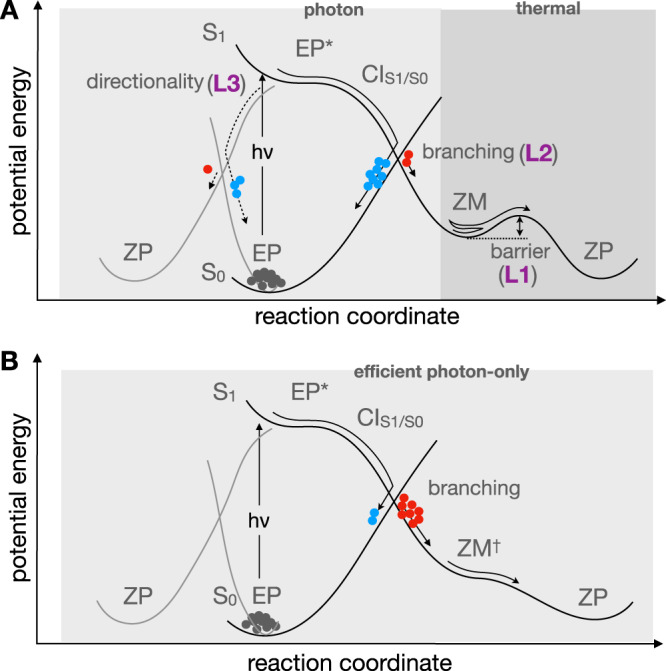
Schematic representation of the reaction path for the first half of LDRM working cycle starting from an *E*P configuration. **A** Classic unidirectional *E*P $\mathop{\to }\limits^{h\nu }$ *Z*M $\mathop{\to }\limits^{{T}^{\circ }}$ *Z*P half-cycle with the possibility of loss of *E*P $\mathop{\to }\limits^{h\nu }$ *Z*P directionality. The labels marking the system potential limitations (see text) are given in purple. **B** Target unidirectional, efficient, and ultrafast *E*P $\mathop{\to }\limits^{h\nu }$ *Z*P half-cycle. The acronyms *E*P, *Z*P, etc. stand for the structures with a specific helicity (P or M) in a specific configuration (*Z* or *E*). CI stands for conical intersection. The gray dots represent the initial structures, the red dots represent the productive trajectories, and the blue dots the unproductive trajectories.

At the level of a single molecule, the operation of a classic light-driven rotary motor (LDRM; see Fig. 1) starting in a *E*P configuration is sketched in Fig. 2A (here we use the general term “configuration” even if the M and P labels indicate the “helicity”). Photoexcitation to the *S*_1_ electronic state results in breaking of the *π*-bond of the axle (*E*P^*^), which releases the strain energy and activates a counterclockwise torsional motion (CCW) (more specifically, precessional)^14^ motion of the rotor with respect to the stator. At nearly orthogonal configurations, the decay to the ground electronic state (*S*_0_) occurs in the vicinity of a conical intersection (CI${}_{{S}_{1}/{S}_{0}}$)^15–17^.

**Fig. 3:**
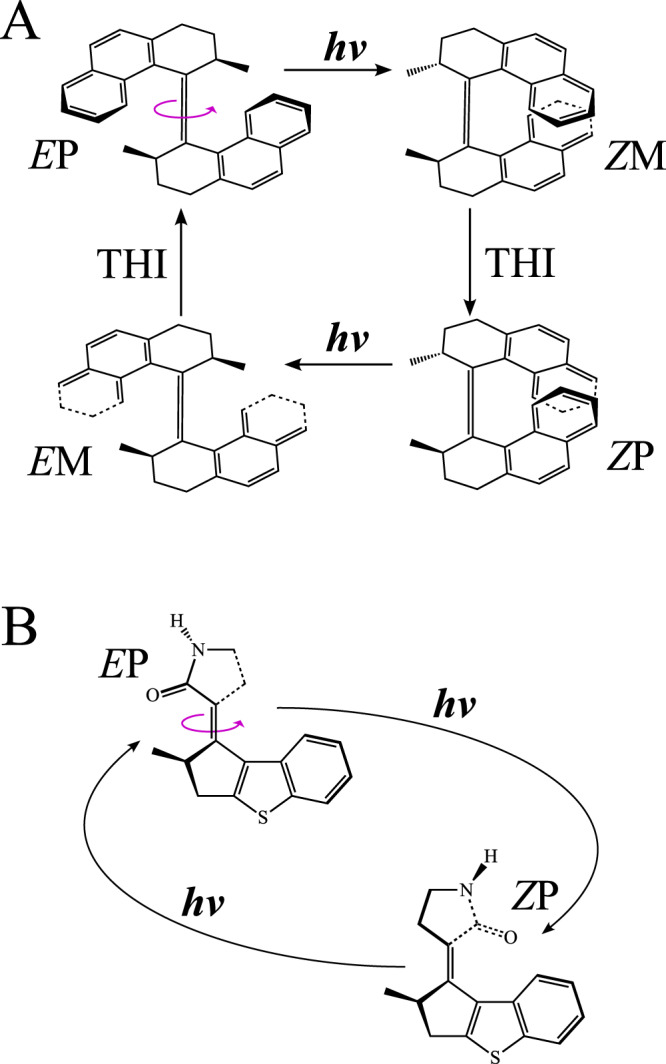
Rotary cycle (also working cycle or photochemical cycle). **A** Rotary cycle of a classic 4-stroke LDRM of Fig. 1. **B** Hypothetical rotary cycle of a 2-stroke LDRM proposed here. The two sequentially absorbed photons are, in general, of different wavelengths.

Upon decay, the immediate restoration of the *π*-bond drives further torsional relaxation in the same direction until formation of a *Z* configuration displaying an opposite M helicity (*Z*M). Subsequently, a thermally activated helix inversion (THI) step^8–10,15,16^, drives the system to a *Z*P configuration suitable for a further light-driven CCW half-rotation^8–10,15,16^. In fact, after absorption of the second photon, the same two-step mechanism is activated, leading to the reconstitution of the original reactant. Thus, the complete working cycle of a classical LDRM (see Fig. 3A) comprises four distinct steps, i.e., *E*P $\mathop{\to }\limits^{h\nu }$ *Z*M $\mathop{\to }\limits^{{T}^{\circ }}$ *Z*P $\mathop{\to }\limits^{h\nu }$ *E*M $\mathop{\to }\limits^{{T}^{\circ }}$ *E*P, where two photochemical steps (power strokes) are interlaced by two THI steps^8–10,15,16^.

The rate-limiting event along each half-cycle is the THI step that occurs on a timescale orders of magnitude longer than the photochemical step completed within a few ps^4,7,10–12,18,19^. THI steps limit the applicability of LDRMs (limitation 1; L1 in Fig. 2A), since the motor operation requires a temperature sufficient to overcome energy barriers of 10–20 kcal/mol. Therefore, there is an ongoing effort to design LDRMs with working cycles avoiding the THI steps or, equivalently, avoid the formation of intermediates *Z*M and *E*M for the CCW rotating motors discussed above (see Fig. 3B).

Several theoretically designed two-stroke LDRMs can be found in the literature^20–22^. However, only limited progress has been reported in their synthesis. In fact, Gerwien et al.^23^ have reported^23^ a photon-only LDRM based on the hemi-thioindigo chromophore, and Boursalian et al. have described^24^ a phosphororganic molecule that required respectively three and four photochemical steps to complete their working cycles.

At the level of molecular ensembles, rather than a single molecule, motor operation is affected by two additional limitations (limitations 2 and 3; L2 and L3 in Fig. 2A). The first is related to the branching occurring at the CI${}_{{S}_{1}/{S}_{0}}$ of each photochemical step, which allows a fraction of the photoexcited molecules to return to the original conformation; i.e., to undergo an ultrafast internal conversion—see the blue circles in Fig. 2.

For alkene-based molecular motors, this branching is expressed in terms of the quantum yield of isomerisation (Φ_iso_) and constitutes the second limitation to the construction of an efficient motor. For instance, the 3-stroke motor designed by Gerwien et al.^23^ does operate at low temperature, but, due to relatively low Φ_iso_ of the three photochemical steps, absorbs on average ∼1900 photons to complete a single 360° rotation^23^. This releases excessive heat in the device per effective rotation cycle, and limits the maximum number of rotation cycles performed before photo-degradation eventually occurs under continuous illumination.

The other additional limitation (see L3 in Fig. 2A) corresponds to a partial loss of CCW unidirectionality at the statistical level. Forces imposing a helical shape on *E*P^*^ may not be sufficient to fully prevent the rotation in the CW direction, e.g., imposed by the environmental effects. In this case a fraction of the *S*_1_ population would lead, after decay at a CI${}_{{S}_{1}/{S}_{0}}$, to a direct reconstitution of the same *E*P intermediate; thus, disrupting the rotary cycle concept.

The discussion above points to the following key properties that an efficient photon-only CCW rotating LDRM (see Fig. 2B) should have: (i) a full unidirectionality, (ii) a high Φ_iso_, (iii) the absence of *Z*M and *E*M intermediates, (iv) a limited number of photochemical steps; i.e., ideally a two-stroke working cycle^22^. Presently, the highest photoisomerisation quantum yield Φ_iso_ reported for overcrowded alkene motors reaches 85% for the so-called first generation motors^25^, which are however characterized by a very slow THI step^26^. The second generation motors have a much faster THI^27^, but a reduced Φ_iso_ in the range 1–20%^28^. However, to the best of our knowledge none of these systems features the absence of the THI steps.

So far, the design of molecules that meet the requirements i–iv has been proven to be difficult^13,28^ because the precise “engineering rules” are still largely unknown, and systematic screening of the efficiency of multiple photon-only candidates through synthesis, photochemical, and spectroscopic characterization is highly impractical.

A viable alternative to the laboratory based screening is the use of computational quantum chemistry methods to select candidates for experimental studies. Such a virtual screening requires the mapping of the reaction paths, necessary to detect L1, and the simulation of the photoinduced population dynamics providing information on L2 and L3. As an important byproduct, theoretical modeling reveals the atomistic details of the successful candidates and, in turn, allows the formulation of novel structure-based engineering rules.

Although, in the past, accurate wavefunction-based quantum chemistry methods have been successfully used for mapping reaction paths, their use in dynamics simulations is cumbersome; especially when the simulations are carried out to screen multiple candidacies. Clearly, less demanding computational methods that can deal with both photochemical and thermal reactions in a balanced way need to be employed.

A rapid characterization of the photo and thermal reactivity of large organic molecules is accessible using the quantum-chemical SSR method^29–31^; see the Supplementary Notes 1 and 3 for the acronym and theoretical details. SSR employs ensemble density functional theory (eDFT)^32–41^ to obtain the ground and excited potential energies but incorporates the pertinent multireference characteristics of the electronic states in a fashion reminiscent of the more traditional wavefunction based methods^29–31,42–44^. The method has been employed to study the dynamics of photoinduced isomerisation in different retinal proteins providing support for its predictive ability^45–47^.

Most importantly in the present context, SSR has been used to model different LDRMs^15,16,22,48–50^. These studies have suggested rules for modifying the photocycle of a classic LDRM^14^, with the scope to overcome the limitations mentioned above^22,48–51^. In particular, SSR enabled the design of **1** (see Fig. 1)^22^, which was predicted to overcome limitation L1 as a consequence of a decrease in steric strain in the fjord region.

However, the preparation of **1** was found to be impractical^22^. Accordingly, the authors envisioned that a bigger but synthetically viable homologue *E*-3’-(2-methyl-2,3-dihydro-1H-benzo[b]cyclopenta[d]thiophen-1-ylidene)pyrrolidin-2’-one (**2**), here abbreviated as MTDP, could display the desired working cycle properties.

Here, we report on a combined computational and experimental study of the MTDP working cycle. We first use the SSR method for a characterization of the entire photocycle of an isolated (i.e., gas phase) MTDP with the scope of justifying successive demanding synthesis. Then, the spectroscopic study of the first half (*E*P $\mathop{\to }\limits^{h\nu }$ *Z*M(?) $\mathop{\to }\limits^{{T}^{\circ }}$ *Z*P) of the working cycle of the synthesized MTDP is carried out. Finally, a set of QM/MM simulations is used to investigate the validity of the gas phase mechanism in the solvent environment.

Currently, an experimental study of the second half (*Z*P → *E*P) does not seem practically achievable due to difficulties with purification of the (meta-stable) *Z*P isomer from the photoequilibrium mixture. However, if the experiments confirm the validity of the computationally derived mechanism for the first half, the validity of the mechanism for the second half should follow by induction.

## Results

### Gas phase theoretical simulations

**Fig. 4:**
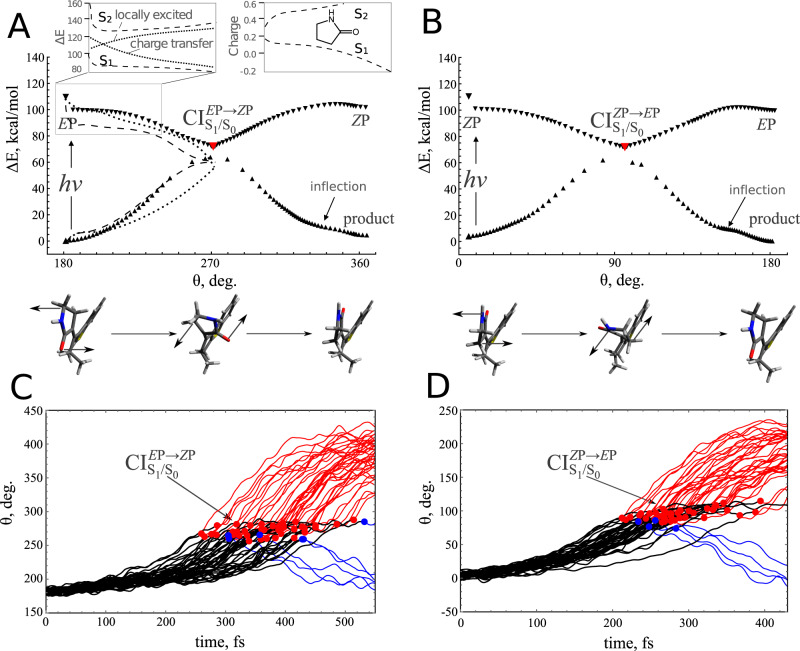
Simulated gas-phase photoisomerisation of MTDP. **A** Projection of the adiabatic minimum energy path (MEP) *E*P → *Z*P of the MTDP motor onto *θ* (see Fig. 1 for the definition of *θ*) calculated with the SSR method (triangles). The structures below the plot show the geometries of the reactant (*E*P), the conical intersection (CI${}_{{S}_{1}/{S}_{0}}$) and the product (ZP). The arrows indicate a CCW motion. The dashed energy profiles correspond to 3-root state-average XMS-CASPT2 energies calculated with a 2 electrons in 2 *π*-orbitals complete active space. The dotted energy profiles show the corresponding 5-root state-average with a 10 electrons in 10 *π*-orbitals complete active space. The insets display the relationship between the *S*_1_ and *S*_2_ states along the framed region. An avoided crossing between a charge transfer and locally excited state is supported by plotting the charge residing on the pyrrolidinone (also called oxindole) ring in the two states. The *S*_1_ → *S*_0_ nonadiabatic relaxation occurs near the geometry of CI${}_{{S}_{1}/{S}_{0}}$, which is shown by the red filled triangle. **B** The same for the *Z*P → *E*P step. **C** *θ* propagation during the quantum-classical population dynamics starting from *E*P. The propagation along the *S*_1_ PES (the black lines) is connected with the productive (the red lines) and unproductive (the blue lines) propagation along the *S*_0_ PES by the corresponding hop points (the red and blue circles) “imaging” a segment of the CI${}_{{S}_{1}/{S}_{0}}$ seam. **D** The same for the *Z*P → *E*P step. Source data are provided as a Source Data file.

The computed *S*_0_ equilibrium geometries of the *R* enantiomer (*S* is, trivially, a mirror image displaying a mirror-image motion. For this reason, *S* is not explicitly considered here; see also the Supplementary Information) of the *E*-**2** and *Z*-**2** diastereomers and of the two corresponding CI${}_{{S}_{1}/{S}_{0}}$’s are shown in Fig. 4A, B, respectively (see the Supplementary Information for more detail). The most stable diastereomer is *E*P, which is ca. 4 kcal/mol below *Z*P. Despite extensive attempts, no stable M-helical configuration has been found by geometry optimization. As will be further discussed below, this is attributed to two factors absent in a classic motor: (1) the strain of the 3-ethylidene-cyclopentene (ECPE) moiety incorporating the MTDP stereogenic center (see the “Discussion” section) and (2) a decrease in the steric repulsion in the MTDP fjord region.

Figure 4A displays the energy profiles computed along the *S*_1_ and *S*_0_ minimum energy paths (MEPs) that compose the *E*P photochemical reaction path (see Supplementary Fig. 8 of the Supplementary Information, for the complete path). The equilibrium geometry of S_0_ *E*P and the corresponding CI${}_{{S}_{1}/{S}_{0}}^{EP\to ZP}$ were used as starting points for the construction of MEPs driven by the torsion angle *θ*.

Photoexcitation to S_1_ is achieved through a *π* → π^*^ transition centered around the C_6_=C${}_{3^{\prime} }$ axle. The transition corresponds to a local excitation, as confirmed by a rather low variation of the Mulliken charge on the pyrrolidinone unit, which, for *E*P, is ∼−0.17 in the *S*_1_ state vs. ∼−0.10 in the *S*_0_ state.

Photoexcitation breaks the *π*-bond of the C_6_=C${}_{3^{\prime} }$ axle, such that the initial *S*_1_ relaxation (not shown) occurs along the bond-length-alternation (BLA) stretching mode corresponding to a rapid lengthening of the C_6_=C${}_{3^{\prime} }$ bond and shortening of the former single bonds (see Supplementary Fig. 8). This enables torsion about the axle in the CCW direction induced, in principle, by the steric strain associated with the P helicity and the fjord region (see Fig. 1) and with the slope on the *S*_1_ potential energy surface (PES).

The system evolves toward CI${}_{{S}_{1}/{S}_{0}}^{EP\to ZP}$ (shown by the red filled triangle), where it decays to *S*_0_. Note that Fig. 4A, B shows MEPs on the adiabatic potential surfaces and the non-adiabatic events, such as the *S*_1_ → *S*_0_ decay, occur in the vicinity of the respective CIs and are not explicitly shown in the plots. The torsion continues on the *S*_0_ PES, where the *Z*P configuration is reached without encountering an M helical local minimum; the *Z*M species occurs only as an inflection (i.e., a flatter region) on the *S*_0_ PES. The same mechanism is obtained when the *Z*P diastereomer of **2** is photoexcited (see Fig. 4B). Hence, an isolated MTDP is predicted not to be affected by the limitation L1.

Notice that, consistently with the previous investigation of analogous neutral motors^51^, the *S*_1_ torsional relaxation induces a change in the electronic character from locally excited to charge transfer. This is demonstrated by the gradual accumulation of the negative charge on the pyrrolidinone unit from −0.17 to −0.53 and from −0.22 to −0.51 along the *S*_1_ branches of the MEP of *E*P-**2** and *Z*P-**2**, respectively (see Supplementary Fig. 9 of the Supplementary Information). The increase in charge separation is characteristic of a so-called twist-BLA CI${}_{{S}_{1}/{S}_{0}}$, and is consistent with the calculated *S*_1_ and *S*_0_ MEP coordinates that describe a rotation with no pyramidalisation at C${}_{3^{\prime} }$ nor C_6_; i.e., an axial rotation and not the precessional motion described  in overcrowded alkene motors^14–16^.

In order to further document the *S*_1_ relaxation mode and the corresponding electronic structure changes, we computed the *S*_0_, *S*_1_ and *S*_2_ energy profiles along the *E*P-**2** MEP using the wavefunction-based multi-state multiconfigurational XMS-CASPT2 method with different active spaces. The results shown in Fig. 4A support the consistency of the XMS-CASPT2 and SSR methods. Furthermore, the *S*_1_ and *S*_2_ energy profiles and the corresponding charge distribution variations (see the insets in Fig. 4A) reveal, consistently with the SSR charge alterations, opposite trends in the electronic character along the *S*_2_ and the *S*_1_ potential energy curves, indicating the presence of an avoided crossing between these states (here, marked by sketching two diabatic curves).

The limitations L2 and L3 have initially been evaluated under isolated conditions by SSR-based quantum-classical dynamics simulations of the time evolution of photoexcited *E*P-**2** and *Z*P-**2** carried out by propagating a set of surface-hop trajectories (see the Supplementary Information for details). The results predict a full unidirectional CCW rotation of the pyrrolidinone rotator (see Fig. 4C, D and the Supplementary Multimedia files) indicating the absence of L3. Both half cycles are ultrafast with *S*_1_ lifetimes of ca. 400 fs and 300 fs for *E*P and *Z*P, respectively (see Supplementary Table 4 for different evaluation methods).

The excited state motion starts with the expected BLA relaxation occurring within the first ca. 100 fs. This is followed by the onset of the double bond twisting ultimately leading to non-adiabatic decay (i.e., in the CI${}_{{S}_{1}/{S}_{0}}^{EP\to ZP}$ and CI${}_{{S}_{1}/{S}_{0}}^{ZP\to EP}$regions) to *S*_0_ after a latency time of ca. 280 fs for *E*P-**2** and 220 fs for *Z*P-**2** rotation, respectively.

Consistent with the MEPs computations, the rotations appear axial (see Supplementary Movies 1 and 2), since no C_6_ or C${}_{3^{\prime} }$ pyramidalisation is detected during progression toward the CI${}_{{S}_{1}/{S}_{0}}$ seam. Most importantly, photoisomerisation ends up in the *Z*P-**2** (when started in *E*P-**2**) and the *E*P-**2** (when started in *Z*P-**2**) diastereomers, without encountering the M helical conformations. Finally, the two steps display Φ_iso_ values, computed as the fraction of trajectories reaching the photoproduct, of 0.87 and 0.91 for *E*P and *Z*P, respectively, suggesting that MTDP should also overcome the limitation L2. However, in the following, we shall see that the solvent effect can significantly decrease such a high directionality and Φ_iso_ values and that these effects are consistent with Φ_iso_ experimentally measured in methanol.

### Chemical synthesis

**Fig. 5:**
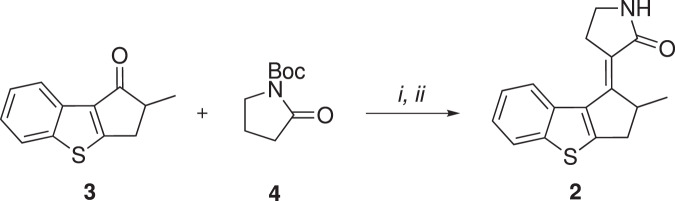
Synthesis of MTDP. Reagents and conditions: (i) LiHMDS, BF_3_ ⋅ Et_2_O, THF dry, −78 °C; (ii) TFA, DCM, 0 °C to room temperature.

In order to validate the computational predictions above, **2** was prepared in racemic (*R* + *S*) form by implementing the strategy suggested in ref. 22 (see Fig. 5). Accordingly, a racemate of the substrate 2-methyl-2,3-dihydro-1H-benzo[b]cyclopenta[d]thiophen-1-one^19^ was conjugated, through aldol condensation, with the commercially available N-Boc pyrrolidinone to obtain a mixture of diastereomers that, after treatment with trifluoroacetic acid, dehydrates and eliminates the protecting group that exclusively yields the *E*-**2** target. Therefore, our measurements have focused on the *E*P → *Z*P transition as an experimental testbed.

The geometrical structure of *E*-**2** was confirmed by the X-ray crystallography (see the Supplementary Note 2). Comparison of the X-ray geometry with the calculated gas phase equilibrium geometry revealed a good match between geometric parameters, with a maximum deviation of the bond lengths of only 0.014 Å; see the Supplementary Note 4. Note that, as anticipated above, in this and in the following comparisons we exclusively focus on the *R* enantiomer of **2**; because *S* is a mirror image, it would display exactly the same properties but rotate in the CW, rather than CCW, direction.

### Transient absorption (TA) spectroscopy

**Fig. 6:**
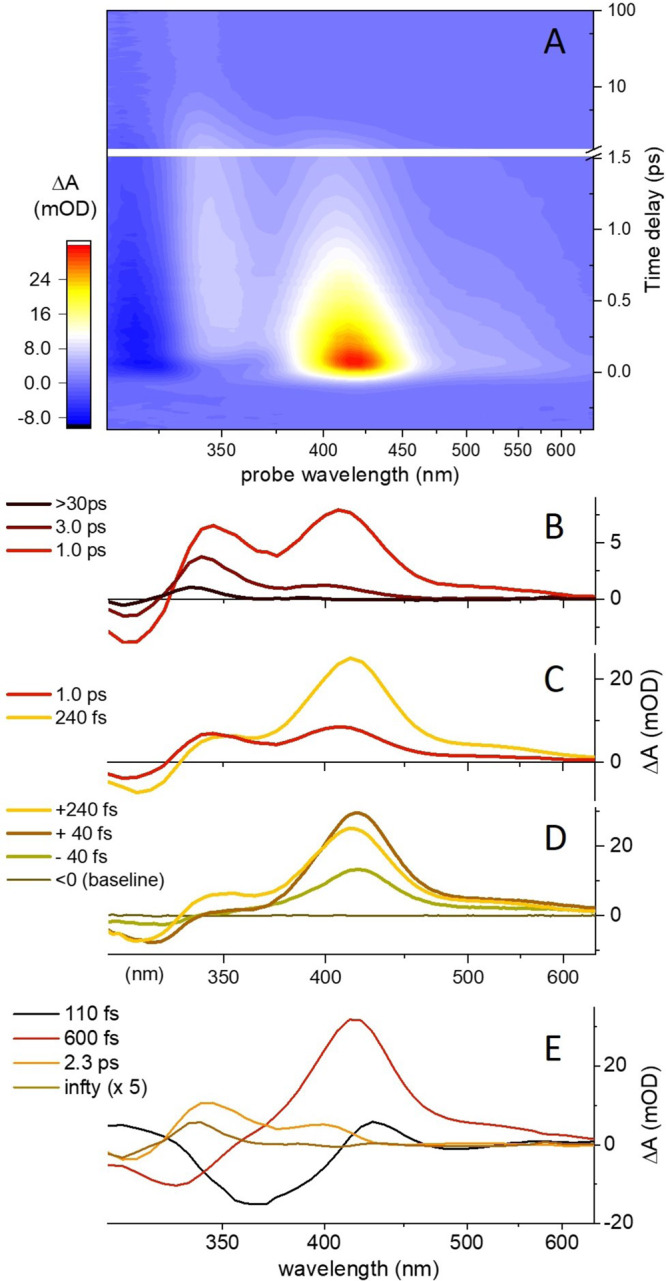
Transient absorption (TA) spectroscopy of a methanol solution of MTDP upon 290 nm light excitation. **A** False-color representation of the pump-induced absorption change (ΔA, in mOD) as a function of probe wavelength (nm) and pump-probe delay (in ps). A selection of TA spectra (ΔA) at late, intermediate, and early pump-probe time delays is displayed in **B**–**D**, respectively. **E** Decay-Associated Spectra (DAS) obtained from the global fit of the entire TA dataset displayed in **A** by a multiexponential kinetics involving 4 time constants (see also Supplementary Fig. 31). Source data are provided as a Source Data file.

The photoisomerisation dynamics of a methanol solution of *E*-**2** (*λ*_max_ = 305 nm) was investigated by femtosecond transient absorption upon 290 nm excitation. As shown in Fig. 6A, the ground state bleach (GSB, negative signal at wavelengths *λ* < 340 nm), and the excited state absorption (ESA, positive signal peaking at *λ* = 420 nm) both rise within the experimental time resolution (−40 fs and +40 fs spectra in Fig. 6D).

During the next 200 fs, the GSB level at *λ* < 320 nm remains constant, suggesting that no significant *S*_1_ → *S*_0_ population transfer occurs; consistently with the computationally predicted latency time of ca. 220–280 fs. The observed simultaneous growth of a secondary ESA band at ∼350 nm accompanied by a slight decay of the 420 nm ESA band is attributed to the early motion away from the FC region, which corresponds to the predicted character change from locally excited to charge transfer. The signal is also consistent with a red shift of the stimulated emission (negative signal), which initially (at 40 fs) overlaps and masks the 350 nm ESA and later on (at 240 fs) overlaps and attenuates the 420 nm ESA band.

The further evolution in Fig. 6C shows that the GSB and 420 nm ESA bands have markedly decayed by 1 ps, indicating significant *S*_1_ to *S*_0_ decay on this time scale. Simultaneously, the 350 nm ESA band is replaced by a 340 nm band assigned to the vibrationally hot *S*_0_ photoproduct. Within the next few ps (Fig. 6B), the 420 nm ESA band rapidly decays and the photoabsorption (PA) signature relaxes, due to *S*_0_ vibrational relaxation and cooling, and partially overlaps with the residual GSB band until a stationary or “final” TA spectrum is observed after 30 ps.

The photoisomerisation time scale is revealed by the global fit^52^ of the TA data with multiexponential decay kinetics (see the Supplementary Note 5 for details). The results of the fit of the dataset of Fig. 6A is displayed in Fig. 6E in the form of decay-associated spectra (DAS). The fastest, resolved time constant (110 fs) describes the spectral evolution assigned to the early motion away from the FC region because, consistent with Fig. 6D, the corresponding DAS reproduces the signal growth (negative amplitude) around 360 nm and the decay (positive amplitude) around 420 nm. The 600 fs DAS clearly displays the decay of the (positive) 420 nm ESA and the (negative) GSB around 320 nm, indicative of the *S*_1_ → *S*_0_ decay. The 2.3 ps DAS mostly reveals the *S*_0_ relaxation kinetics with further refilling of GSB due to the 340 nm PA band relaxation.

Finally, the “infinite” time DAS describes the final TA spectrum, Δ*A*^*∞*^(*λ*), observed beyond 30 ps. It is proportional to the difference between the steady-state photoproduct (*Z*-**2**) and reactant (*E*-**2**) absorption spectra (see Supplementary Note 5). This means that the stable *Z* diastereomer is already produced after decay to *S*_0_ and subsequent vibrational cooling by ∼30 ps. — i.e., in a single step. This is in marked contrast to classic 4-stroke motors, e.g., the oxindole motor^51^, where the “final” TA spectrum observed by ∼50 ps corresponds to the spectrum of a metastable configuration (e.g., *Z*M), which relaxes on a much slower timescale of THI (μs — ms) to the spectrum of a stable configuration (e.g., *Z*P)^51^.

Although the environmental effects were missing in the non-adiabatic trajectory calculations, the predicted 280 fs latency time required for initiating the double bond torsion and the 400 fs population decay time are not far from the experimentally assigned few hundred fs *S*_1_ vibrational relaxation and ca. 600 fs lifetime in methanol solution. Most importantly, the predicted absence of a *S*_0_ metastable species is consistent with the observation of a stable spectrum less than 30 ps after *S*_1_ decay.

The Φ^iso^ for the *E*-to-*Z* photoisomerisation of MTDP in MeOH at the 290 nm excitation wavelength has been determined using the final TA spectrum Δ*A*^*∞*^(*λ*). Using definition of Φ^iso^, one has:

1 $$\begin{array}{r}{{\Delta }}{A}^{\infty }(\lambda )={x}_{E}\,{{{\Phi }}}^{{{{{{\rm{iso}}}}}}}\,[{A}_{Z}(\lambda )-{A}_{E}(\lambda )],\end{array}$$

where *A*_*E*_(*λ*) and *A*_*Z*_(*λ*) are the known steady-state absorbance of the *E* and *Z* species (see Supplementary Note 5), and *x*_*E*_ is the proportion of *E*-**2** species excited by the pump pulse, i.e., the *E*-**2** excitation probability. Hence, Φ^iso^ can be inferred provided that *x*_*E*_ is known.

The excitation probability *x*_*E*_ was calibrated using *trans* azobenzene (*t* AB) as an actinometer. Two TA measurements (one for a reference methanol solution of *t*AB and one for a fresh methanol solution of *E*-**2**) were performed under the same pump and probe conditions. Then, from Eq. (1) and the known Φ^iso^ and absorption spectra of *c*AB and *t*AB in MeOH^53–55^, the excitation probability *x*_*E*_ was calibrated and Φ^iso^ of **2**-*E*P(Φ^iso^ = 0.25 ± 0.05) was obtained. This value is lower than the theoretical Φ^iso^ of an isolated **2**-*E*P molecule in the gas phase; therefore, suggesting that Φ^iso^ of **2** is strongly influenced by the environment.

### Multiscale QM/MM theoretical simulations

The computationally derived mechanism of CCW rotation documented above is assumed to remain invariant in the solvent environment. This assumption was tested by simulating the photoinduced dynamics of *E*P in a methanol solution using quantum-classical trajectory surface hopping (TSH) simulations. This has required the construction of an *S*_0_ quantum mechanics/molecular mechanics (QM/MM) model of *E*P in methanol. A limited set of room-temperature initial conditions have then been generated starting from the constructed model and used to initiate the trajectory propagation in *S*_1_ employing different quantum chemical (CASSCF and SSR) and surface-hop (FSSH^56,57^ methods and DISH-XF^58–60^) to ensure the general validity of the result. The details are given in Supplementary Notes 3 and 4.

The above room-temperature simulations show that the methanol environment does not alter the axial isomerisation mechanism or the electronic characteristics observed for the isolated *E*P model (see Supplementary Movies 4–9) but significantly alters the statistical quantities associated with the system dynamics. In fact, the number of computed trajectories (ca. 40) enables one to obtain information, from which the longer time spent in *S*_1_, the decrease in reactivity, and the longer time required to reach the final configuration (*Z*P) are found to be closer to the experimental observations than to the gas phase simulations. Most importantly, after the *S*_1_ → *S*_0_ relaxation, the slow progression toward the *Z*P configuration (see the above mentioned Supplementary Movies) was related to the existence of a solvent-solute hydrogen bond network restricting the M → P change of helicity and forcing the molecule to reside in a transient M-helical configuration for several ps (from 1 ps to over 10 ps according to a FSSH study of a set of 40 CCW trajectories). The QM/MM TSH simulations also showed that very few trajectories moved in the CW direction and that these relaxed to *E*P rather than reaching *Z*M or *Z*P after decay at CI${}_{{S}_{1}/{S}_{0}}$ (see Fig. 2A). In all cases, the simulations in solution suggest a relatively low quantum efficiency, because the majority of the CCW trajectories are found to be unproductive.

In general, we concluded that the number of CCW rotating trajectories that successfully reached the *Z*P isomer in methanol are substantially decreased compared to isolated conditions. This brings the theoretical simulation closer to the experiment. The results of the TSH simulations are confirmed by the QM/MM optimization of MEPs in methanol, which points (similar to the gas phase) to the absence of stable M helicity.

## Discussion

The general agreement between the simulated dynamics and the spectral evolution of the photoisomerisation of the *E*-**2** diastereomer supports the computationally derived mechanism. This implies that the CCW direction of torsion is imposed by the initial P helicity in the vicinity of the FC geometry^8^, while the slope of the *S*_1_ MEP drives the rotator in the CCW direction until the *Z* diastereomer is reached through a concerted process. Although it was currently impossible to carry out femtosecond TA studies of the *Z*-**2** diastereoisomer, the simulations point to the same photon-only concerted mechanism for the *Z*P → *E*P photoisomerisation; hence, for the second half of the rotary cycle. Indeed an additional set of trajectories starting from the room-temperature equilibrated *Z*P model in methanol, display the same axially rotating photoisomerisation mechanism seen in the *E*P simulation (see Supplementary Movie 10). The trajectories also indicate a perfect CCW unidirectionality of rotation (no L3), a faster isomerisation process and, potentially, a higher reactivity (i.e., reduced L1 and L2) with respect to the *E*P half-cycle discussed above. For instance, the productive trajectory analysis (see Supplementary Movies 4, 6–8) clearly shows that the molecule spends a limited amount of time in the *E*M configuration before finally generating the starting diastereoisomer *E*P.

The experimentally confirmed photon-only *E*P → *Z*P transformation allows us to investigate the origin of the removal of the THI step in MTDP. In an LDRM, the favored helicity is defined by the absolute configuration of the ring-embedded stereogenic center of the stator (see Fig. 1)^8^. In fact, there is a direct relationship between the axial position of the methyl group at the *R* stereogenic center and the P helicity of the fjord region. Most relevantly, the *Z*M to *Z*P THI occurs concurrently with the equatorial to axial ring inversion of the methyl^8^.

**Fig. 7:**
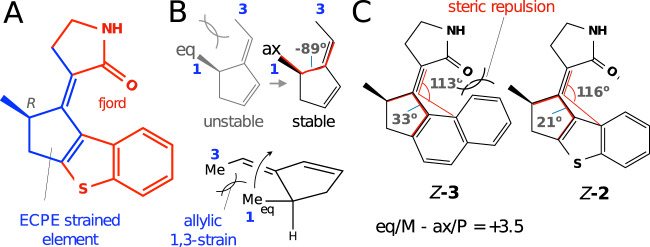
Illustration of the factors contributing to absence of the stable M conformations in MTDP. **A** *Z*-**2** structure highlighting the strained unit (blue) and the clashing fjord region (red). **B** Pictorial illustration of the equatorial to axial relaxation imposed by the strain in a model of the ECPE unit. In the equatorial position the Me in position 1 is almost parallel/aligned with the methyl substituent in position 3. The large dihedral angle (in red) in the axial conformer is consistent with removal of the strain. **C** Geometrical parameters (planar and dihedral angles in red) justifying the reduced steric repulsion in *Z*-**2** vs. *Z*-**3**. The energy difference (kcal/mol) between the M and P conformers of *Z*-**3** is also given.

We will now show that, in contrast to the classic motor based on a cyclohexene stator (see Fig. 1), the inclusion of the cyclopentene-based strained element ECPE in *Z*-**2** (see Fig. 7A) leads to destabilization of the equatorial position of the methyl substituent (the unit chirality is necessary as an achiral unit will generate energetically equivalent P and M configurations). This is demonstrated by the computational search for an equatorial conformation of the ECPE model in Fig. 7B, which exclusively produced an axial conformation. This behavior, that points at the removal of THI, is attributed to the allylic 1,3-strain^61,62^. As documented in Supplementary Note 4, such a strain is decreased in the corresponding cyclohexene-based element found in classical motors that show stable equatorial and axial conformers and, therefore, a THI step.

The ECPE strain pushing the methyl substituent to an axial position must drive the molecule to the P-helicity. However, this can only occur if the steric repulsion in the fjord region is decreased. This is demonstrated by additional computations of the model compound **3** (see Fig. 7C). In fact, *Z*-**3**, a close analogue of *Z*-**2**, has both equatorial and axial conformers. This is attributed to the steric repulsion caused by the proximity of the carbonyl group of the rotator and the terminal phenyl group of the stator. This steric repulsion is relaxed in *Z*-**2** due to the presence of the five-membered thiophene moiety that places the terminal stator unit at a larger distance from the rotator.

However, it should be realized that such a decrease in strain must also have an impact on the level of unidirectionality that leads to L3. This means that a trade-off between a high level of directionality and THI removal has to be achieved in the ideal two-photon candidate. Of course, a more quantitative estimate of this effect requires a larger number of TSH simulations; a goal that goes beyond the present qualitative/mechanistic study.

### Conclusions

In summary, the design and preparation of MTDP-like systems and, possibly, of an entire library of prospective 2-stroke motors must be guided by engineering rules ensuring the demonstrated axial rotation, unidirectionality and absence of stable M-intermediates. As we previously reported, the first requirement can be achieved by modulating the S_0_ homolytic and heterolytic breaking of the central double bond (the axle). Narrowing the gap between the heterolytic (usually, disfavored) and homolytic bond breaking enables one to switch from a twist-pyramidalisation (typical in classic LDRMs) to a twist-BLA geometry at the CI; thus, achieving a nearly perfect axial rotation^14,22,48,50,51^. Concurrently, this enhances accessibility of the CI seam, which may lead to a faster photoisomerisation and, possibly, an enhanced quantum yield^14,51^. The second requirement has instead been defined in the present work in terms of the presence of a chiral strained unit (e.g., ECPE) and a stator “geometry” allowing for a decreased steric repulsion in the fjord region (e.g., including a pentalene-like unit with two fused five-membered rings). When applying the two rules above, it is recommended to combine chemical intuition with proper computational modeling performed by appropriate computational tools; such as those employed in this work.

In this work, we have provided combined computational and experimental evidence that the *E*P → *Z*P half rotary cycle of **2** has the properties of a photon-only LDRM and disentangled the structural requirements associated with such behavior. Since SSR MEP and trajectory calculations (both in isolated conditions and in the QM/MM model in solution) indicate that the complementary *Z*P → *E*P half-cycle should display the same properties, the presented results appear to open a realistic route toward the preparation of prospective photon-only 2-stroke rotary motors.

## Methods

### Materials

Details of the synthesis and characterization of the MTDP motor are reported in Supplementary Note 2.

### Computational methods

In this work, the state-interaction state-averaged spin-restricted ensemble-referenced Kohn-Sham (SI-SA-REKS, or SSR) method^29–31,48,63^ is used to obtain the total energies, the forces on the nuclei, and the nonadiabatic couplings. The SSR method employs eDFT^32–41^ to introduce the strong non-dynamic correlation into the description of the ground and excited electronic states of molecules and to obtain the excitation energies in a time-independent fashion reminiscent of the multi-configurational methods of wavefunction theory. The use of eDFT enables a seamless incorporation of the multi-reference effects into the computations, where the results of the standard KS computations are recovered for the weakly correlated (single-reference) systems while providing a much improved description of the systems with dissociating chemical bonds, biradical and polyradical electronic states, and electronic states with pronounced charge transfer^30,31^. The use of ensemble representation leads to the occurrence of the fractional occupation numbers of several frontier KS orbitals, which can be used to characterize the strength of the multi-configurational correlation effects.

The decoherence induced surface hopping from exact factorization (DISH-XF) method^58,64^ combines the electronic equations derived from the exact factorization of the electronic-nuclear wavefunction^65–69^ with the conventional TSH formalism^70^. The exact factorization enables one to seamlessly incorporate the effect of nuclear quantum momentum, which depends on the shape of nuclear distribution, into the classical equations of motion for the nuclei. In this way, the decoherence of the nuclear trajectories is achieved seamlessly^58^.

All the quantum chemical computations are carried out using the local version of the GAMESS-US program (2018.v3)^71,72^, which implements the SSR method and the analytic derivatives formalism^73^. All the calculations employ the 6-31G* basis set^74^ and the BH&HLYP exchange-correlation density functional^75–77^. The geometry optimizations are performed using the DL-FIND module^78^ interfaced with GAMESS-US. The geometries of the CIs are optimized by the CIOpt program^40^ with the penalty function formalism and using the analytic energy gradients of the intersecting states. The gas-phase NAMD simulations are performed by the pyUNI-xMD program^64^, a standalone code which implements the DISH-XF method^79^. The multiscale QM/MM calculations (including the NAMD) have been performed using the MOLCAS/Tinker interface^80,81^. The XMS-CASPT2 calculations have been performed using OpenMolcas^82^.

More information on the computational methods and the detail of computations can be found in Supplementary Note 3. The results of the theoretical simulations are reported in Supplementary Note 4.

### Transient absorption measurements

A fresh methanol solution of *E*-**2** was prepared to an absorbance A = 1.1/mm at its absorption maximum of λ_max_ = 306 nm. The sample was investigated by femtosecond TA spectroscopy with a pump-probe setup described elsewhere^83^. In short, we used the 800-nm, 40-fs pulse of a Ti:sapphire regenerative amplifier (amplitude, operating at 5 kHz repetition rate) to pump a commercial, tunable optical parametric amplifier (TOPAS; Light Conversion) followed by fourth harmonic generation to produce a 290-nm pump pulse. A white-light continuum was generated with the fundamental 800 nm pulse in a 2 mm thick CaF_2_ crystal, and used as a probe pulse offering an absorption detection window spanning from 310 to 750 nm. The polarization of the pump beam was set at the magic angle (54.7 degrees) with respect to that of the probe. Both pump and probe beams were focused and overlapped in the sample, which was circulated in a 0.5-mm-thick cuvette with a peristaltic pump. The choice of a sub-300-nm pulse enables detecting the GSB signal (GSB observed from 310 to ∼350 nm) without any experimental noise due to pump light scattering, since the pump pulse lies outside this probe detection window. The pump power is adjusted within the linear regime of excitation, corresponding to pulse energies in the range of ∼1 mJ/cm^2^ or below. Quantitative data analysis was performed by singular value decomposition, followed by the global fitting of the dominating four singular transients; the analysis employed a multiexponential decay convoluted by a Gaussian function modeling the instrument response function. More details about data acquisition, processing and analysis can be found in Supplementary Note 5.

## Supplementary information

Supplementary Information

Description of Additional Supplementary Files

Supplementary Movie 1

Supplementary Movie 2

Supplementary Movie 3

Supplementary Movie 4

Supplementary Movie 5

Supplementary Movie 6

Supplementary Movie 7

Supplementary Movie 8

Supplementary Movie 9

Supplementary Movie 10

## Data Availability

Crystallographic data for the structure in this paper were deposited on the Cambridge Crystallographic Data Centre under accession code CCDC 2150133. Copy of the data can be obtained, free of charge, on application to CCDC, 12 Union Road, Cambridge CB2 1EZ, UK; (fax: +44(0) 1223 336 033; or e-mail: deposit@ccdc.cam.ac.uk). Supplementary Movies 1 and 2 show the *E*P → *Z*P and *Z*P → *E*P gas phase trajectories, Supplementary Movie 3 shows animation of the complete working cycle of the motor, and Supplementary Movies 4–10 show the QM/MM trajectories in methanol solution. Source data are provided with this paper.

## Source data

Source Data

## Code availability

The computer code used in this research is available from the corresponding author (M.F.) upon request.
