# Author Correction: Towards the engineering of a photon-only two-stroke rotary molecular motor

**DOI:** 10.1038/s41467-022-35006-w

**Published:** 2022-12-07

**Authors:** Michael Filatov(Gulak), Marco Paolino, Robin Pierron, Andrea Cappelli, Gianluca Giorgi, Jérémie Léonard, Miquel Huix-Rotllant, Nicolas Ferré, Xuchun Yang, Danil Kaliakin, Alejandro Blanco-González, Massimo Olivucci

**Affiliations:** 1 Department of Chemistry, Kyungpook National University, Daegu, 702-701 South Korea; 2 Dipartimento di Biotecnologie, Chimica e Farmacia, Università di Siena, Via A. Moro 2, 53100 Siena, Italy; 3 Institut de Physique et Chimie des Matériaux de Strasbourg, Université de Strasbourg, CNRS UMR 7504, Strasbourg, France; 4 Institut de Chimie Radicalaire (UMR-7273), Aix-Marseille Université, CNRS, 13397 Marseille Cedex 20, France; 5 Chemistry Department, Bowling Green State University, Overmann Hall, Bowling Green, OH 43403 USA

**Keywords:** Light harvesting, Atomistic models, Excited states, Energy transfer

Correction to: *Nature Communications* 10.1038/s41467-022-33695-x, published online 28 October 2022

The original version of this Article contained errors in the reference list, in which reference [80] “Aquilante, F. et al. Molcas 8: new capabilities for multiconfigurational quantum chemical calculations across the periodic table. *J. Comput. Chem.* 37, 506–541 (2016)” was incorrectly given as [81], reference [81] “Ponder, J. W. & Richards, F. M. Tinker molecularmodeling package. *J. Comput. Chem.* 8, 1016–1024 (1987)” was incorrectly given as [82], reference [82] “Fdez Galván, I. et al. OpenMolcas: from Source Code to Insight. *J. Chem. Theory Comput.* 15, 5925–5964 (2019)” was incorrectly given as [83], and reference [83] “Briand, J. et al. Coherent ultrafast torsional motion and isomerization of a biomimetic dipolar photoswitch. *Phys. Chem. Chem. Phys.* 12, 3178–3187 (2010)” was incorrectly given as [80]. This has been corrected in the PDF and HTML versions of the Article.

