# Supplementary material for: Towards the engineering of a photon-only two-stroke rotary molecular motor
Source: Nat Commun. 2022 Oct 28;13:6433. doi: 10.1038/s41467-022-33695-x (PMC9616945; doi:10.1038/s41467-022-33695-x)
Supplement: Supplementary file 1 — Supplementary Information [file 41467_2022_33695_MOESM1_ESM.pdf]

Supplementary Information: Towards the engineering of a photon-only two-stroke rotary molecular motor

Michael Filatov(Gulak),[*,†,‖] Marco Paolino,[*,‡,‖] Robin Pierron,[¶,‖] Andrea Cappelli,[‡] Gianluca Giorgi,[‡] Jérémie Léonard,[*,¶] Miquel Huix-Rotllant,[§] Nicolas Ferré,[§] Xuchun Yang,[‡,⊥] Danil Kaliakin,[‡,⊥] Alejandro Blanco González,[‡,⊥] and Massimo Olivucci[*,‡,⊥]

†*Department of Chemistry, Kyungpook National University, Daegu 702-701, South Korea*

‡*Dipartimento di Biotecnologie, Chimica e Farmacia, Università di Siena, Via A. Moro 2, 53100 Siena, Italy*

¶*Institut de Physique et Chimie des Matériaux de Strasbourg, Université de Strasbourg, CNRS UMR 7504, Strasbourg, France*

§*Institut de Chimie Radicalaire (UMR-7273), Aix-Marseille Université, CNRS, 13397 Marseille Cedex 20, France*

‖*Contributed equally to this work*

⊥*Chemistry Department, Bowling Green State University, Overmann Hall, 43403 Bowling Green, OH, USA*

E-mail: mike.filatov@gmail.com; paolino3@unisi.it; Jeremie.Leonard@ipcms.unistra.fr; olivucci@unisi.it

Contents

1 Supplementary Note 1. List of acronyms used in the main article S4

2 Supplementary Note 2. Synthesis and characterization S5

2.1 Reagents S5

2.2 Synthesis S5

2.3 X-Ray crystallography and mass-spectrometry S6

3 Supplementary Note 3. Computational Methods S9

3.1 SSR method S9

3.2 DISH-XF method S10

3.3 Computational details S12

4 Supplementary Note 4. Theoretical Simulations S13

4.1 Adiabatic potential energy surfaces in the gas phase S13

4.1.1 Ground and excited state species S13

4.1.2 Comparison of the theoretically obtained S_0 equilibrium geometry with the X-ray crystallographic geometry S17

4.1.3 Minimum Energy Paths S19

4.1.4 The absence of the M diastereomers S22

4.1.5 Vertical excitation energies and absorption spectra of *E*P and *Z*P configurations S28

4.2 Non-adiabatic molecular dynamics simulations in isolated conditions S30

4.3 Non-adiabatic molecular dynamics simulations in solution S36

4.3.1 Stationary Points and Adiabatic Minimum Energy Paths S39

4.3.2 QM/MM NAMD Simulations of the *E*P Diastereoisomer S44

4.3.3 QM/MM NAMD Simulations of the *Z*P Diastereoisomer S55

4.4 Evaluation of the total rotation quantum yield under steady state conditions S60

5 Supplementary Note 5. Transient Absorption (TA) spectroscopy of *E*-2 S63

5.1 Preliminaries: steady-state absorption spectra of the pure isomers S63

5.2 TA Experimental set-up S64

5.3 TA Data analysis S64

5.4 Photoisomerisation quantum yield of MTDP S65

5.4.1 Preliminary comment on the excitation probability S65

5.4.2 Using tAB as actinometer to determine η_E S67

Supplementary References S69

1 Supplementary Note 1. List of acronyms used in the main article

BLA	bond length alternation
*c*AB	*cis*-azobenzene
CASSCF	complete active space self-consistent field method
CI	Conical Intersection
CCW	Counterclockwise
CW	Clockwise
DAS	decay associated spectra
ECPE	3-ethylidene-cyclopentene
eDFT	ensemble density functional theory
ESA	excited state absorption
FC	Franck-Condon
GSB	ground state bleach
LDRM	Light Driven Rotary Motor
MEP	Minimum Energy Path
MTDP	*E*-3′-(2-methyl-2,3-dihydro-1H-benzo[b]cyclopenta[d]thiophen-1-ylidene)pyrrolidin-2′-one
PA	photoabsorption
PES	potential energy surface
QM/MM	quantum mechanics / molecular mechanics (multi-scale modelling technique)
SE	stimulated emission
SI	Supporting Information
SSR	State-interaction State-averaged spin-Restricted ensemble-referenced Kohn-Sham method
TA	transient absorption
*t*AB	*trans*-azobenzene
TFA	trifluoroacetic acid
THI	Thermal Helix Inversion
TSH	trajectory surface-hopping
XMS-CASPT2	extended multi-state complete active space second-order perturbation theory method

2 Supplementary Note 2. Synthesis and characterization

2.1 Reagents

Reagent grade chemicals were used for the synthesis. Yields refer to purified products and are not optimized. Column chromatography uses the Merck silica gel 60 (230-400 mesh). Thin layer chromatography (TLC) uses the Merck TLC plates and silica gel 60 F254. Melting points were determined in open capillaries on a Gallenkamp apparatus and are uncorrected. The ^{1}H NMR spectra were recorded at 600 MHz and the ^{13}C NMR were recorded at 150 MHz using the Bruker DRX-600 AVANCE spectrometer in the indicated solvents (TMS is used as the internal standard). The values of the chemical shifts are expressed in ppm and the coupling constants (J) in Hz. The Agilent 1100 LC/MSD spectrometer operating with an electrospray source was used in the mass spectrometry experiments.

2.2 Synthesis

(E)-3-(2-methyl-2,3-dihydro-1H-benzo[b]cyclopenta[d]thiophen-1-ylidene)pyrrolidin-2-one (MTDP, **2**) molecule was synthesized in the following way: To a solution of N-Boc-2-pyrrolidinone (**4**) (0.17 g, 0.94 mmol) dissolved in anhydrous THF (5 mL), a 1 M solution of lithium hexamethyldisilazide (LiHMDS) in anhydrous THF (1.08 mL, 1.08 mmol) was added at -78 °C under the nitrogen atmosphere. After 15 min., a solution of compound **3**[1] (0.20 g, 0.99 mmol) and $BF_3{\cdot}Et_2O$ (1.49 mmol, 122 μL) in anhydrous THF (3 mL) was added dropwise. The reaction mixture was stirred at -78 °C for 3 h. Then, NH_4Cl (s.s.) was added, and the crude was extracted with dichloromethane. The combined organic layers were dried over sodium sulphate and concentrated under reduced pressure. The residue was dissolved in dichloromethane (5.0 mL) and trifluoroacetic acid (0.6 mL) was added. The resulting reaction mixture was stirred at room temperature for 30 min. Then, $NaHCO_3$ (s.s.) was added and the crude was

extracted with dichloromethane. The residue was purified by flash chromatography using ethyl acetate/petroleum ether (1:1) as eluent to obtain compound MTDP as pale yellow solid (0.13 g, yield 55%). An analytical sample (melting point 238.2-238.9 °C) was obtained by crystallization from a solution of methanol-dichloromethane (8:2) by slow evaporation of the solvent. ^{1}H NMR (600 MHz, $CDCl_3$): 1.31 (d, *J* = 6.9, 3H), 2.65 (d, *J* = 16.7, 1H), 3.19-3.39 (m, 2H), 3.43-3.53 (m, 2H), 3.56-3.66 (m, 1H), 4.55 (m, 1H), 6.59 (br s, 1H), 7.31 (t, *J* = 7.5, 1H), 7.38 (t, *J* = 7.5, 1H), 7.80 (d, *J* = 8.0, 1H), 8.05 (d, *J* = 8.1, 1H). ^{13}C NMR (150 MHz, $CDCl_3$): 22.7, 30.3, 37.2, 40.4, 43.0, 115.7, 123.5, 123.8, 124.0, 124.6, 133.5, 137.2, 138.9, 145.0, 152.2, 173.5. MS(ESI): *m/z* 292.1 (M + Na^+).

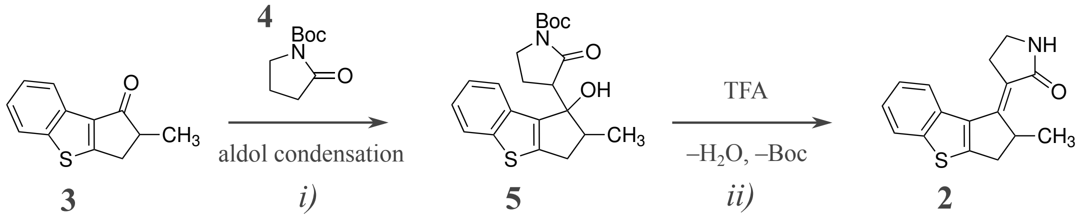

Supplementary Figure 1: Synthesis of MTDP (**2**). Reagents and conditions: (*i*) LiHMDS, $BF_3 \cdot Et_2O$, THF dry, -78 °C; (*ii*) TFA, DCM, 0 °C to room temperature.

2.3 X-Ray crystallography and mass-spectrometry

Single crystal of MTDP was submitted to X-ray data collection on the Oxford-Diffraction Xcalibur Sapphire 3 diffractometer with a graphite monochromated Mo-Kα radiation (λ = 0.71073 Å) at 293 K. The structures were resolved by direct method implemented in the SHELXS-97 program.[2] The refinements were carried out by the full-matrix anisotropic least squares method on F2 for all reflections for non-H atoms by means of the SHELXL program.[3] Crystallographic data for the structure in this paper were deposited on the Cambridge Crystallographic Data Centre as supplementary publication no. CCDC 2150133 (MTDP). Copy of the data can be obtained, free of charge, on application to

CCDC, 12 Union Road, Cambridge CB2 1EZ, UK; (fax: +44(0) 1223 336 033; or e-mail: deposit@ccdc.cam.ac.uk).

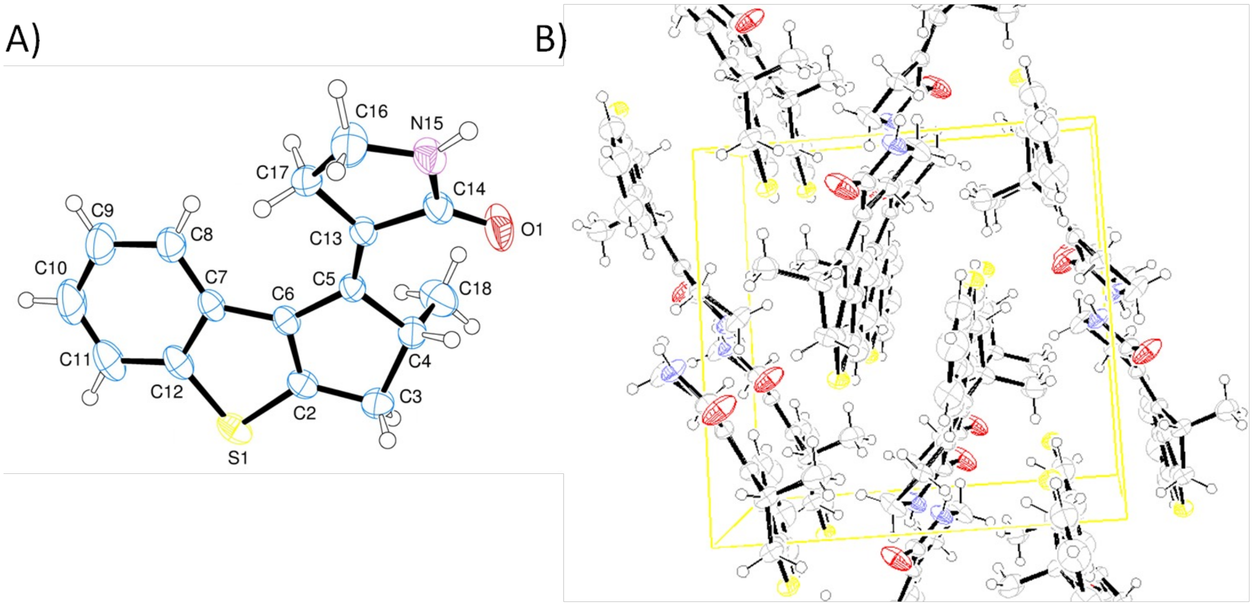

Supplementary Figure 2: Crystallographic structure of racemic compound MTDP; A) Detail of R enantiomer and B) crystallographic cell unit showing R and S enantiomers. Ellipsoids enclose 50% probability.

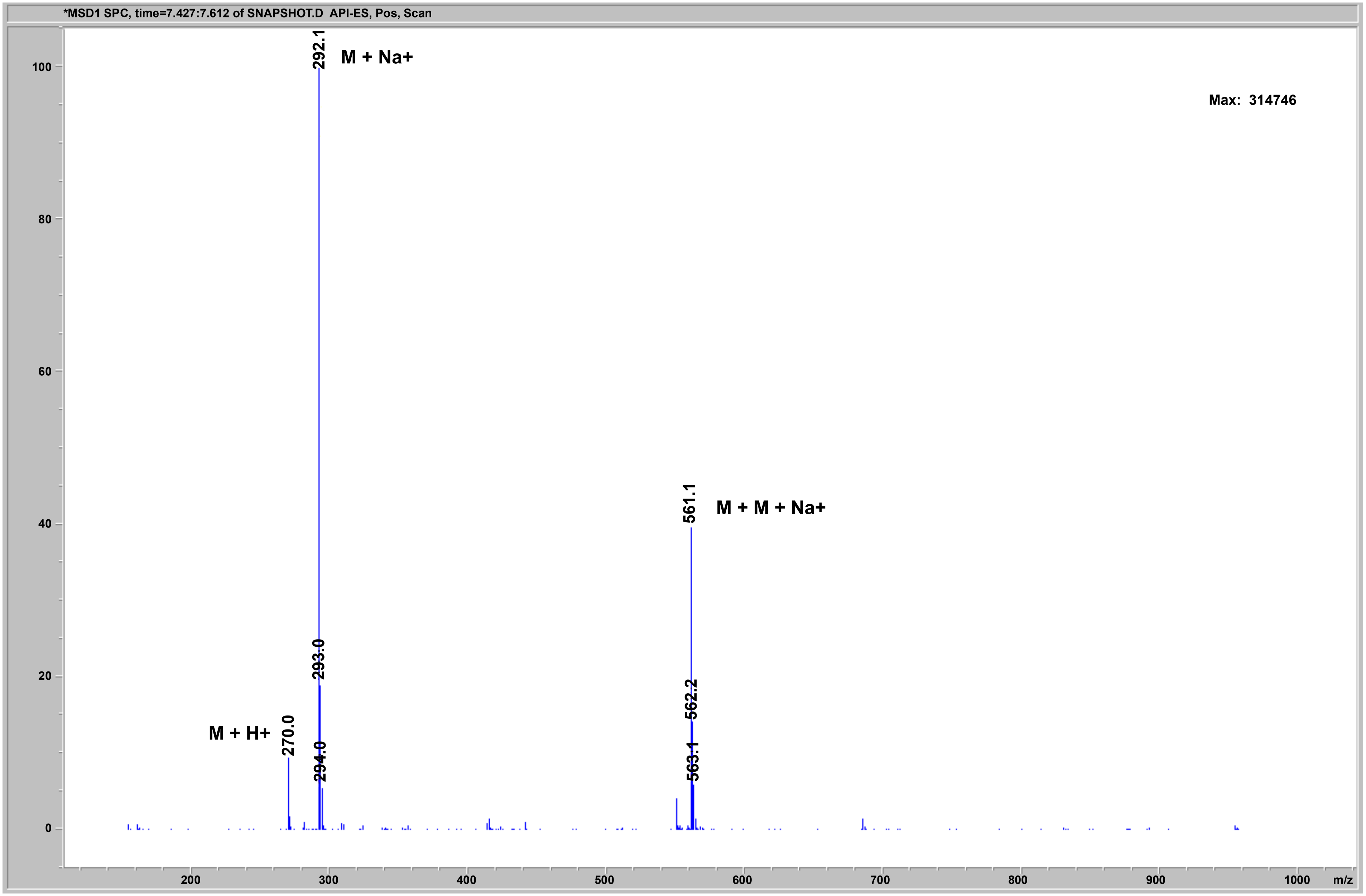

Supplementary Figure 3: Electrospray ionisation mass spectrum (ESI MS) of MTDP. M/Z of the main peak at 292.1 amu corresponds to M + Na^{+}, which yields 269.1 amu as the mass M of MTDP.

3 Supplementary Note 3. Computational Methods

3.1 SSR method

The SSR method[4–9] employed in this work to obtain the ground and excited electronic states energies, forces on the nuclei (the analytic gradient), and the non-adiabatic coupling (NAC) vector employs ground state eDFT[10–15] to describe the non-dynamic electron correlation occurring due to multireference character of the ground state and eDFT for ensembles of ground and excited states[16–19] to obtain excitation energies from a variational time-independent formalism. The ensemble representation of the density and energy of a strongly correlated electronic state results in occurrence of the fractional occupation numbers (FONs) of several frontier KS orbitals. Here, the SSR(2,2) method is used, where two fractionally occupied orbitals accommodate two electrons in total. The energies of the ground S_0 and excited S_1 states are obtained in SSR(2,2) from variational optimization of an ensemble of a perfectly spin-paired singlet (PPS) electronic configuration and an open-shell singlet (OSS) configuration with respect to the KS orbitals and their FONs (the SA-REKS(2,2)[6] orbital optimization), which is followed by solving a 2×2 secular problem

$$\begin{pmatrix} E_0^{PPS} & \Delta_{01}^{SA} \\ \Delta_{01}^{SA} & E_1^{OSS} \end{pmatrix} \begin{pmatrix} a_{00} & a_{01} \\ a_{10} & a_{11} \end{pmatrix} = \begin{pmatrix} E_{S_0} & 0 \\ 0 & E_{S_1} \end{pmatrix} \begin{pmatrix} a_{00} & a_{01} \\ a_{10} & a_{11} \end{pmatrix} \tag{1}$$

for the actual S_0 and S_1 states.[7–9] The interstate coupling parameter Δ_{01}^{SA} is calculated simultaneously with the E_0^{PPS} and E_1^{OSS} energies when optimizing the KS orbitals (and their FONs) in the SA-REKS(2,2) calculation.[7–9]

For the S_0 and S_1 energies, the analytic gradients are available.[20–22] The gradients of the S_0 and S_1 states are related to the gradients of the PPS and OSS states and the coupling

element Δ_{01}^{SA} as

$$\nabla E_{S_k} = a_{kk}^2 \nabla E_0^{PPS} + a_{lk}^2 \nabla E_1^{OSS} + 2\, a_{kk}\, a_{lk}\, \nabla \Delta_{01}^{SA}\,; \quad l \neq k\,; \quad l,k = 0,1 \tag{2}$$

From the gradients ∇E_{S_k} and ∇E_0^{PPS} and ∇E_1^{OSS}, the NAC vector $\mathbf{H}_{01} = \langle \mathrm{S}_0 | \nabla \mathrm{S}_1 \rangle = \langle \mathrm{S}_0 | \nabla \mathbf{H} | \mathrm{S}_1 \rangle / (E_{S_1} - E_{S_0})$ between S_0 and S_1 is obtained as

$$\mathbf{H}_{01} = \frac{1}{E_{S_1} - E_{S_0}} \left(\frac{a_{00}^2 - a_{10}^2}{2\, a_{00}\, a_{10}} \left(\mathbf{G}_{01} - (a_{00}^2 - a_{10}^2)\, \mathbf{g}_{01} \right) + 2\, a_{00}\, a_{10}\, \mathbf{g}_{01} \right), \tag{3}$$

where

$$\mathbf{G}_{01} = \frac{1}{2} (\nabla E_{S_0} - \nabla E_{S_1}) \tag{4}$$

$$\mathbf{g}_{01} = \frac{1}{2} (\nabla E_0^{PPS} - \nabla E_1^{OSS})\,. \tag{5}$$

3.2 DISH-XF method

The DISH-XF method has been described in detail elsewhere[23,24] and here only a brief account of its basic features will be given. The DISH-XF method combines the electronic equations derived from the exact factorization of the electronic-nuclear wavefunction[25–29] with the conventional TSH formalism.[30] The exact factorization enables one to seamlessly incorporate the effect of nuclear quantum momentum, which depends on the shape of nuclear distribution, into the classical equations of motion for the nuclei.

DISH-XF is a mixed quantum-classical method where the electronic degrees of freedom are treated quantum mechanically and the classical description in terms of trajectories is used for the nuclear degrees of freedom. For a given nuclear trajectory $\mathbf{R}^{(I)}$, the time-dependent electronic wavefunction $\Phi_{\mathbf{R}^{(\mathbf{I})}}(\mathbf{r}, t)$ is expanded in terms of the wavefunctions of the Born-Oppenheimer (BO) states as $\Phi_{\mathbf{R}^{(\mathbf{I})}}(\mathbf{r}, t) = \sum_l C_l^{(I)}(t) \phi_l(\mathbf{r}; \mathbf{R}^{(I)}(t))$ and the time evolution of an element

$\rho_{lk}^{(I)}(t) = C_l^{(I)*}(t)C_k^{(I)}(t)$ of the reduced density matrix $\rho^{(I)}(t)$ is described by

$$\frac{d}{dt}\rho_{lk}^{(I)}(t) = \frac{i}{\hbar}\left\{E_l^{(I)}(t) - E_k^{(I)}(t)\right\}\rho_{lk}^{(I)}(t) - \sum_j \left\{\sigma_{lj}^{(I)}(t)\rho_{jk}^{(I)}(t) - \rho_{lj}^{(I)}(t)\sigma_{jk}^{(I)}(t)\right\}$$
$$+ \sum_j \left\{Q_{jl}^{(I)}(t) + Q_{jk}^{(I)}(t)\right\}\rho_{lj}^{(I)}(t)\rho_{jk}^{(I)}(t) \quad (6)$$

where $E_l^{(I)}$ and $\phi_l(\mathbf{r};\mathbf{R}^{(I)}(t))$ are the energy and the wavefunction of the l-th BO state, $\sigma_{jk}^{(I)}$ is a non-adiabatic coupling matrix element between the jth and kth BO electronic states, and $Q_{jk}^{(I)}$ is a term that gives quantum correction to the nuclear motion arising from electronic-nuclear correlations;[27–29] the latter term is derived from the exact factorization of the electronic-nuclear wavefunction.[23] In Eq. (6),the superscript (I) indicates a quantity obtained at the nuclear configuration $\mathbf{R}^{(I)}$.

In Eq. (6), the $Q_{jk}^{(I)}$ term yields coupling between the nuclear quantum momenta $i\hbar\nabla_\nu|\chi|/|\chi|$ and the electronic phases $\mathbf{f}_{\nu,j}^{(I)}$ and $\mathbf{f}_{\nu,k}^{(I)}$

$$Q_{jk}^{(I)} = \sum_\nu \frac{i\hbar}{M_\nu}\frac{\nabla_\nu|\chi|}{|\chi|}\Bigg|_{\mathbf{R}^{(I)}(t)} \cdot \left(\mathbf{f}_{\nu,j}^{(I)} - \mathbf{f}_{\nu,k}^{(I)}\right) \quad (7)$$

where M_ν is a mass of the νth nucleus. The nuclear quantum momenta are obtained from a number of auxiliary nuclear trajectories generated for electronic states other than the running state l. Although the concept of auxiliary trajectories for decoherence-induced surface hopping dynamics is not new,[31] the correction used here is derived from the exact quantum equations and no further renormalisation of the electronic density matrix is needed.

An auxiliary trajectory $\mathbf{R}^{(I)}(t')$ is generated when a nonzero $\rho_{kk}(t')$ is encountered at a time t' and is evolved classically with a uniform velocity obtained from the energy conservation law. The electronic phase term $\mathbf{f}_{\nu,k}^{(I)} = -\int^t \nabla_\nu E_k^{(I)}(t')dt'$ is evaluated by time integration of the momentum changes at the kth BO state. To calculate the

quantum momentum, a fictitious Gaussian nuclear density $|\chi_k|^2$ with a uniform variance σ is associated with the auxiliary trajectory at each BO state. Then, the nuclear quantum momentum is $\nabla_\nu|\chi|/|\chi|(\mathbf{R}^{(\mathbf{I})}(\mathbf{t})) = -\frac{1}{2\sigma^2}(R_\nu^{(I)}(t) - \langle R_\nu^{(I)}(t)\rangle)$, where $\langle R_\nu^{(I)}(t)\rangle = \sum_k \rho_{kk}^{(I)} R_{k\nu}^{(I)}(t)$. The uniform variance σ can be either obtained from the initial distribution of nuclear trajectories or set as a parameter. Here, a uniform value $\sigma = 0.1$ a.u. is used.

Nuclear trajectories follow the Newtonian equations of motion on the PES of the running state l, *i.e.*, the force on the νth nucleus is $\mathbf{F}_\nu^{(I)} = -\nabla_\nu E_l^{(I)}$. When the surface hop probability becomes greater than a random number, a surface hop is initiated. The surface hop probability from the running state l to another state $k(\neq l)$ at a time interval $[t, t + \Delta t]$ is calculated as

$$P_{l\to k} = \frac{2\Re\left[\rho_{lk}^{(I)}(t)\sigma_{lk}^{(I)}(t)\right]}{\rho_{ll}^{(I)}(t)}\Delta t\,, \tag{8}$$

where the same constraints as in the Tully's fewest switches algorithm[30,32] are imposed on the hopping transition; *i.e.*, when $E_k^{(I)}$ is greater than the total energy, the transition is forbidden and, when a negative hopping probability is obtained (*e.g.*, due to the phase factor of $\rho_{lk}^{(I)}(t)$), the probability is set to zero. Upon a successful surface hop, the nuclear velocities are rescaled to satisfy the total energy conservation, and the running state is switched to the new state.

3.3 Computational details

All the quantum chemical computations are carried out using the local version of the GAMESS-US program (2018.v3),[33,34] which implements the SSR method and the analytic derivatives formalism.[20] All the calculations employ the 6-31G* basis set[35] and the BH&HLYP exchange-correlation density functional.[36–38] The geometry optimizations are performed using the DL-FIND module[39] interfaced with GAMESS-US. The geometries

of the conical intersections are optimized by the CIOpt program[40] with the penalty function formalism and using the analytic energy gradients of the intersecting states.

The gas-phase NAMD simulations are performed by the pyUNI-xMD program,[24] a standalone code which implements the DISH-XF method.[23] The NAC vectors Eq. (3) and the energy gradients ∇E_{S_k} $(k = 0, 1)$ were calculated by the GAMESS-US program. The nuclear equations of motion are integrated using the velocity-Verlet algorithm with the time step of 20 a.u. (0.48 fs). The electronic equations of motion (6) are integrated by the 4-th order Runge-Kutta method with the time step of 0.002 a.u. (4.8×10^{-5} fs). When integrating the electronic equations of motion, the electronic energies $E_l^{(I)}$ and the non-adiabatic $\sigma_{lk}^{(I)}$ couplings are linearly interpolated between the end points of the integration interval of the nuclear equations of motion.

The initial conditions at the start of the NAMD trajectories were set up by sampling the Wigner function of a canonical ensemble of harmonic oscillators.[41,42] The trajectories starting in the *E*P configuration were propagated for 1200 steps (576fs) and the trajectories starting in the *Z*P conformation for 900 steps (432fs). The trajectories were propagated using the NVE ensemble, *i.e.*, the microcanonical ensemble, where the total number of particles N, the system's volume V and the total (electronic + nuclear kinetic) energy E are conserved.

4 Supplementary Note 4. Theoretical Simulations

4.1 Adiabatic potential energy surfaces in the gas phase

4.1.1 Ground and excited state species

Here, the MTDP (**2**) molecular rotary motor (MTDP: (E)-3-(2-methyl-2,3-dihydro-1H-benzo[b]cyclopenta[d]thiophen-1-ylidene)pyrrolidin-2-one) is first studied computationally to establish detailed mechanism of its photoisomerisation. As

was demonstrated in the previous computational study,[43] a simpler analogue of the MTDP motor, the DTPN (**1**) motor (DTPN: 3-(3,5-dimethyl-5,6-dihydro-4H-cyclopenta[b]thiophen-4-ylidene)pyrrolidin-2-one) is capable of performing a full revolution of its rotor part by absorbing photons only, *i.e.*, without involvement of the thermally activated helix inversion steps. The working cycle of the MTDP

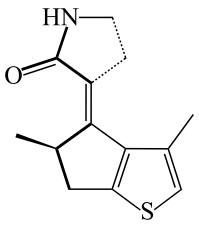

1

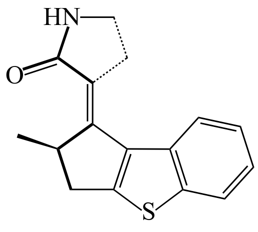

2

motor can thus be presented as in Supplementary Figure 4. Upon completion of the photoisomerisation step, the motor molecule ends up in a configuration suitable for the further photoisomerisation step in the same sense of torsion; *i.e.*, if the first isomerisation step started in the *E*P configuration, it ends up in the *Z*P conformer and not in the ZM diastereoisomer.

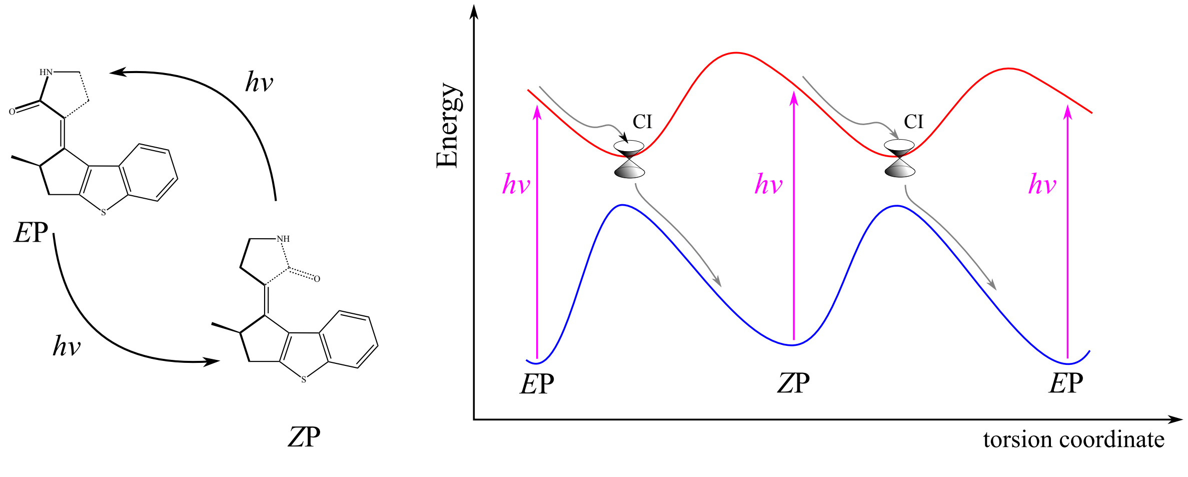

Supplementary Figure 4: Working cycle of a 2-stroke molecular rotary motor studied in this work. The right panel shows schematically the ground and excited electronic state PESs along the torsional coordinate.

The numbering of atoms in the MTDP motor is shown in Supplementary Figure 5 along with the definition of the central dihedral angle θ. The equilibrium geometries

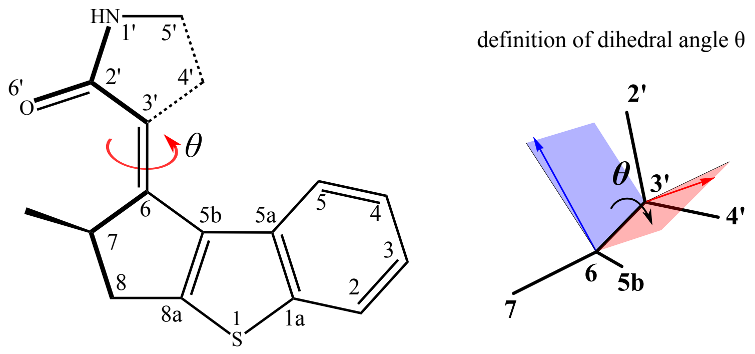

Supplementary Figure 5: Chemical formula and atomic numbering of MTDP motor studied in this work. Also shown is the definition of the central dihedral angle θ.

of the *E*P and *Z*P conformers of MTDP in the ground S_0 electronic state were optimized using the SSR-BH&HLYP/6-31G* method (see the Supporting information for the Cartesian coordinates of the atoms). The lowest singlet excited state S_1 corresponds to the $\pi \rightarrow \pi^*$ one-electron transition, which leads to breaking of the π-component of the central C_6=$C_{3'}$ double bond. Breaking the C_6=$C_{3'}$ bond results in reorganization of the single and double bonds along the $C_{2'}$–$C_{3'}$=C_6–C_{5b}=C_{8s} chain known as the bond length alternation (BLA); typically defined as the difference between the average lengths of formal single bonds and formal double bonds, *i.e.*, $\mathrm{BLA} = {}^1\!/\!{}_2(R_{2'-3'} + R_{6-5b}) - {}^1\!/\!{}_2(R_{6-3'} + R_{5b-8a})$. The S_1/S_0 conical intersections (CIs) were optimized as described in Section 3.3. Two minimum energy CIs corresponding to torsion through 90° and 270° were obtained; the CIs are labelled as CI_{90} and CI_{270}, respectively.

The relative energies and the most important geometric parameters of the S_0 minima and CIs are reported in Supplementary Table 1. The geometries of the four species are presented in Supplementary Figure 6. As can be seen in Supplementary Figure 6, the CI_{90} and CI_{270} geometries do not feature pyramidalisation of the carbon atoms at the central C_6=$C_{3'}$ bond, *i.e.*, these geometries are reached by (nearly) pure torsion in combination with the BLA distortion. As was shown in Ref. 44, the absence of pyramidalisation at the CI geometry is a pre-requisite of efficient photoisomerisation.

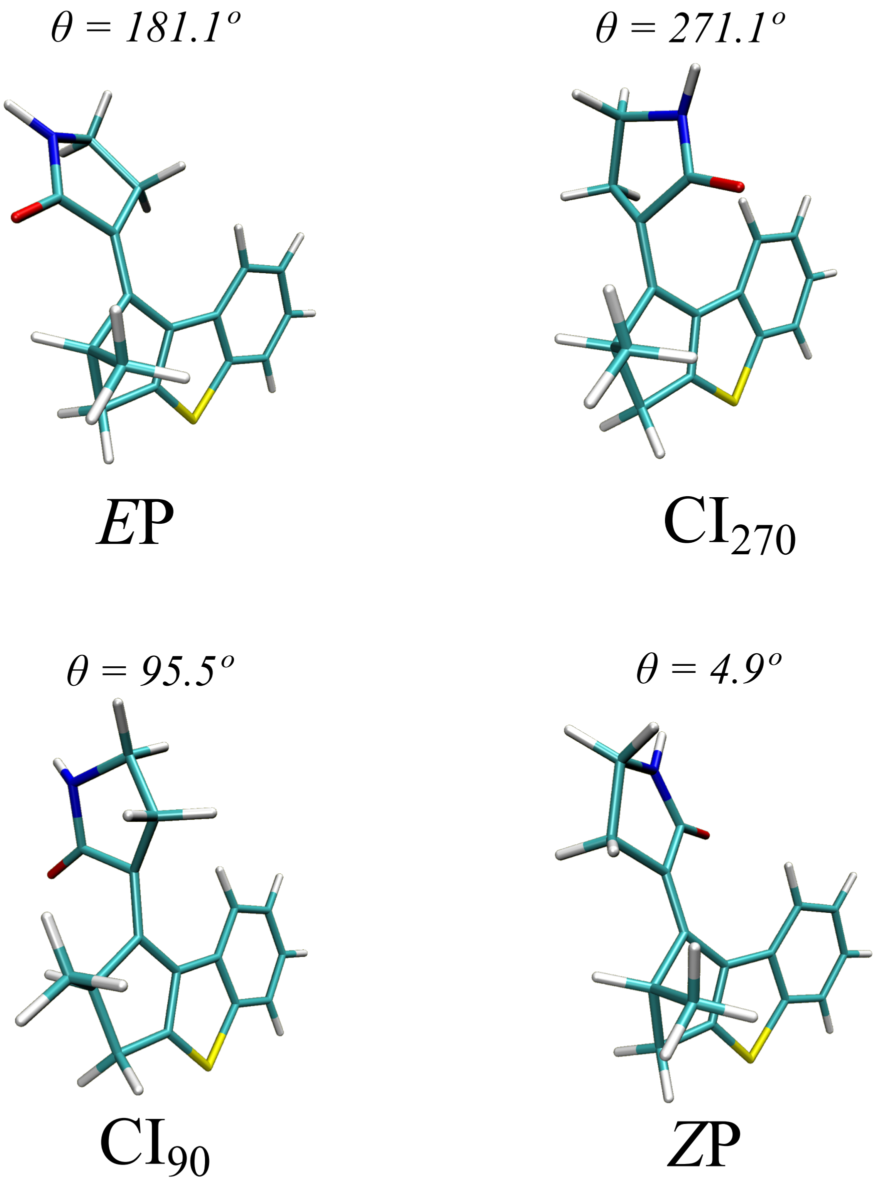

Supplementary Figure 6: Molecular geometries of the ground state minima and conical intersections of MTDP. For definition of the central dihedral angle θ, see Supplementary Figure 5.

Supplementary Table 1: Relative energies ΔE (in kcal/mol) of the S_0 and S_1 states, values of the central dihedral angle θ (in deg.), the bond length alternation (BLA, in Å), the C–O bond length (in Å), and the total charges on the pyrrolidinone unit (in $\bar{e}$) of the respective conformations of the MTDP motor **2**.

configuration	$\Delta E(S_0)$	$\Delta E(S_1)$	θ	BLA	$R(C_{2'}–O_{6'})$	$Q(S_0)^a$	$Q(S_1)^b$
*E*P	0	109.7	181.1	0.133	1.213	-0.10	-0.17
*Z*P	4.0	110.7	4.9	0.130	1.210	-0.09	-0.22
CI_{90}	72.0	72.0	95.5	0.016	1.228	-0.13	-0.53
CI_{270}	72.1	72.1	271.1	0.016	1.227	-0.17	-0.51

a) Total charge on pyrrolidinone in the S_0 state.
b) Total charge on pyrrolidinone in the S_1 state.

*E*P is the most stable diastereoisomer of MTDP, with the *Z*P diastereoisomer lying 4 kcal/mol higher in energy. The vertical excitation energy (VEE) at the *E*P geometry is *ca.* 4.76 eV (109.7 kcal/mol); a slightly lower VEE of 4.63 eV (106.7 kcal/mol) is obtained for the *Z*P diastereoisomer. The S_1/S_0 CIs occur at almost the same energy of *ca.* 72 kcal/mol (3.12 eV) with respect to the *E*P S_0 energy. Hence, there is a substantial driving force (potential difference of *ca.* 1.5 eV) for photoisomerisation starting in both *E*P and *Z*P configurations. At the CI geometries, there is a noticeable equalization of the lengths of the single and double bonds along the $C_{2'}–C_{3'}=C_6–C_{5b}=C_{8s}$ chain; see the BLA values in Supplementary Table 1. This equalization is typical for the CIs reached by torsion and BLA displacement.[44–46]

4.1.2 Comparison of the theoretically obtained S_0 equilibrium geometry with the X-ray crystallographic geometry

The theoretically obtained S_0 equilibrium geometry of the most stable, *E*P, conformer of MTDP is compared in Supplementary Figure 7 with the geometry determined by the X-ray crystallography. There is a good agreement between the experimental and theoretical geometries; the root mean square deviation (RMSD) for the heavy atoms is

only 0.075 Å. Positions of the hydrogen atoms cannot be accurately determined by the X-ray crystallography;[47] hence, they were excluded from the comparison.

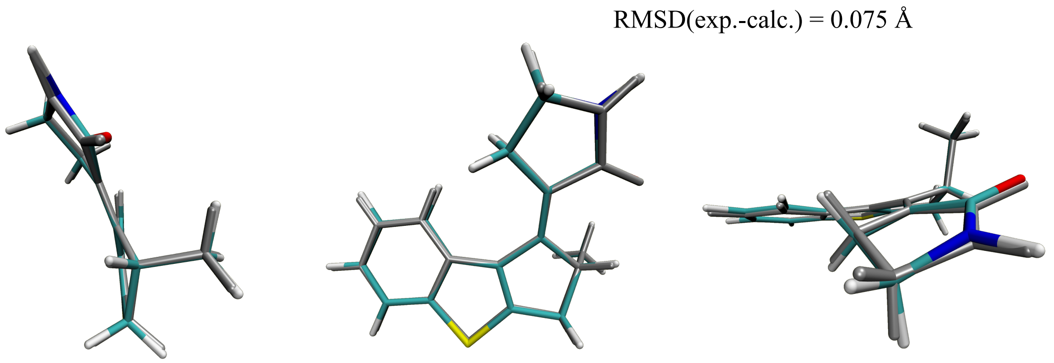

Supplementary Figure 7: Molecular geometry obtained from the X-ray crystallographic measurement (dark grey colour) superimposed against the geometry of the *E*P S_0 minimum (conventional colours) optimized with the SSR-BH&HLYP/6-31G* method. Left panel – front view, middle panel – side view, right panel – top view. The RMSD between the heavy atoms in the two geometries is also given.

Supplementary Table 2: Comparison of the key geometric parameters of the MTDP motor **2** obtained in the X-ray crystallographic measurements (exp.) with the parameters of the *E*P S_0 equilibrium geometry optimized with the SSR-BH&HLYP/6-31G* method in gas phase (calc.).

parameter	exp.	calc.	parameter	exp.	calc.
θ,$^{a)}$ °	177.2	181.1	$C_{2'}$=$O_{6'}$, Å	1.233	1.213
BLA,$^{b)}$ Å	0.133	0.133	S_1–C_{8a}, Å	1.709	1.722
$C_{2'}$–$C_{3'}$, Å	1.489	1.488	S_1–C_{1a}, Å	1.736	1.751
C_6=$C_{3'}$, Å	1.338	1.339	C_{5a}–C_{5b}, Å	1.454	1.451
C_6–C_{5b}, Å	1.472	1.470	$N_{1'}$–$C_{2'}$, Å	1.337	1.364
C_{5b}=C_{8a}, Å	1.357	1.353	$N_{1'}$–$C_{5'}$, Å	1.435	1.443

a) Central dihedral angle; see Scheme 5 for definition.
b) Bond length alternation (BLA) along the $C_{2'}$–$C_{3'}$=C_6–C_{5b}=C_{8s} chain. BLA is defined as the difference between the average formal single bond in the chain and the formal double bond.

Supplementary Table 2 reports the most important geometric parameters, the bondlengths, BLA, and θ, of the *E*P conformer obtained theoretically and experimentally.

There is a slight difference of *ca.* 3.9° between the experimental and theoretical value of the central dihedral angle θ. This difference is likely caused by the effect of the crystal environment (the theoretical calculations were performed in the gas phase). For the bondlengths, the biggest deviations from the experimental values are observed for the S–C bondlengths, *ca.* 0.013–0.014 Å. The other theoretically obtained bondlengths are in a very good agreement with the experimental values.

4.1.3 Minimum Energy Paths

Using the optimized geometries of the S_0 species and CIs minimum energy paths (MEPs) were prepared and optimized using the nudged elastic band (NEB) method with fixed end points.[39] The NEB method has an advantage that it converges to a MEP provided that sufficient number of discrete images are used.[48] The *E*P and *Z*P S_0 minima and CI_{90} and $CI_2$70 were arranged in the order of the increasing central dihedral angle θ and used as the endpoints of the MEPs. The first MEP proceeds on the S_1 potential energy surface (PES) and comprises four segments: *E*P S_0min $\rightarrow$ CI_{270} (30 images), CI_{270} $\rightarrow$ *Z*P S_0min (40 images), *Z*P S_0min $\rightarrow$ CI_{90} (30 images), CI_{90} $\rightarrow$ *E*P S_0min (40 images); when joined, the segments complete a full loop of the motor. The second MEP comprises the same segments and proceeds on the S_0 PES. At the geometries of the CIs, the two MEPs meet. The optimized MEPs are shown in Supplementary Figure 8 in terms of two internal degrees of freedom, the dihedral angle θ and the BLA distortion.

Right after the vertical excitation to the S_1 state at the FC geometry, the *E*P and *Z*P configurations undergo rapid relaxation along the BLA coordinate; the latter reaches near zero values, which indicates weakening and lengthening of the double bonds and certain contraction of the single bonds in the $C_{2'}$–$C_{3'}$=C_6–C_{5b}=C_{8s} chain. This is a consequence of breaking the π component of the $C_{3'}$=C_6 bond in the S_1 state, which leads to reorganization of single and double bonds along the chain and enables rotation about the $C_{3'}$=C_6 bond's axis. In the vicinity of both the *E*P and the *Z*P FC points, there is

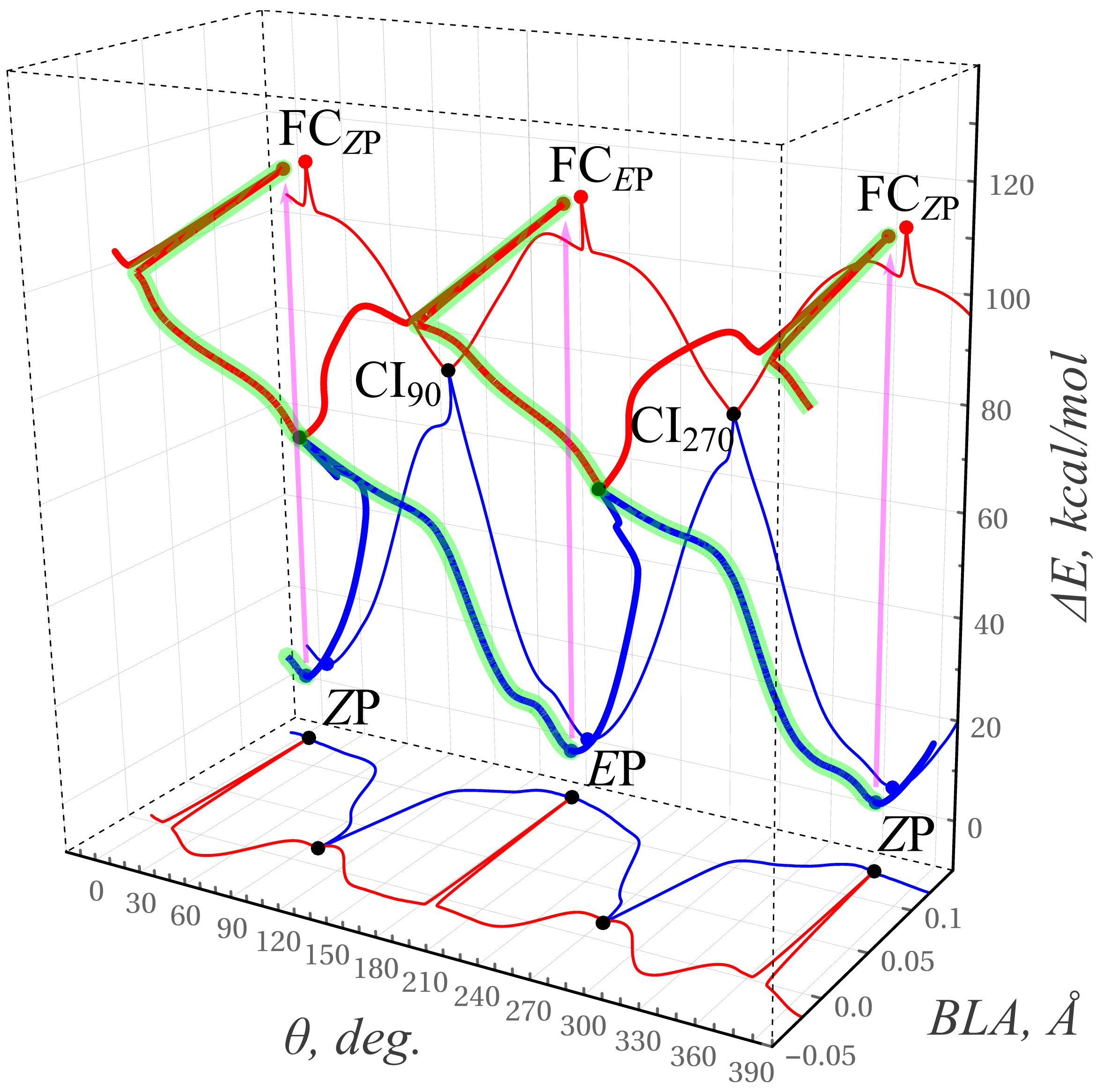

Supplementary Figure 8: Minimum energy pathways on the S_0 (blue) and S_1 (red) PESs of the MTDP motor. The adiabatic reaction path is highlighted in green. Vertical excitations are shown by magenta arrows.

a noticeable slope on the S_1 PES inclined in the direction of the counter clockwise (CCW) rotation of the pyrrolidinone blade towards the geometry of the respective CIs.

Continuing torsion in the CCW direction, the molecule reaches CI_{90} or CI_{270} and undergoes a non-adiabatic population transfer to the S_0 state. Both CIs, CI_{90} and CI_{270}, have pronounced peaked topography and represent efficient funnels for the transfer. As

seen in Supplementary Figure 8, the CIs occur slightly after the top of the barriers on the S_0 PES corresponding to the torsion about the $C_{3'}$=C_6 bond. This suggests that an adiabatic path on the S_0 PES should follow in the same CCW direction as on the S_1 PES.

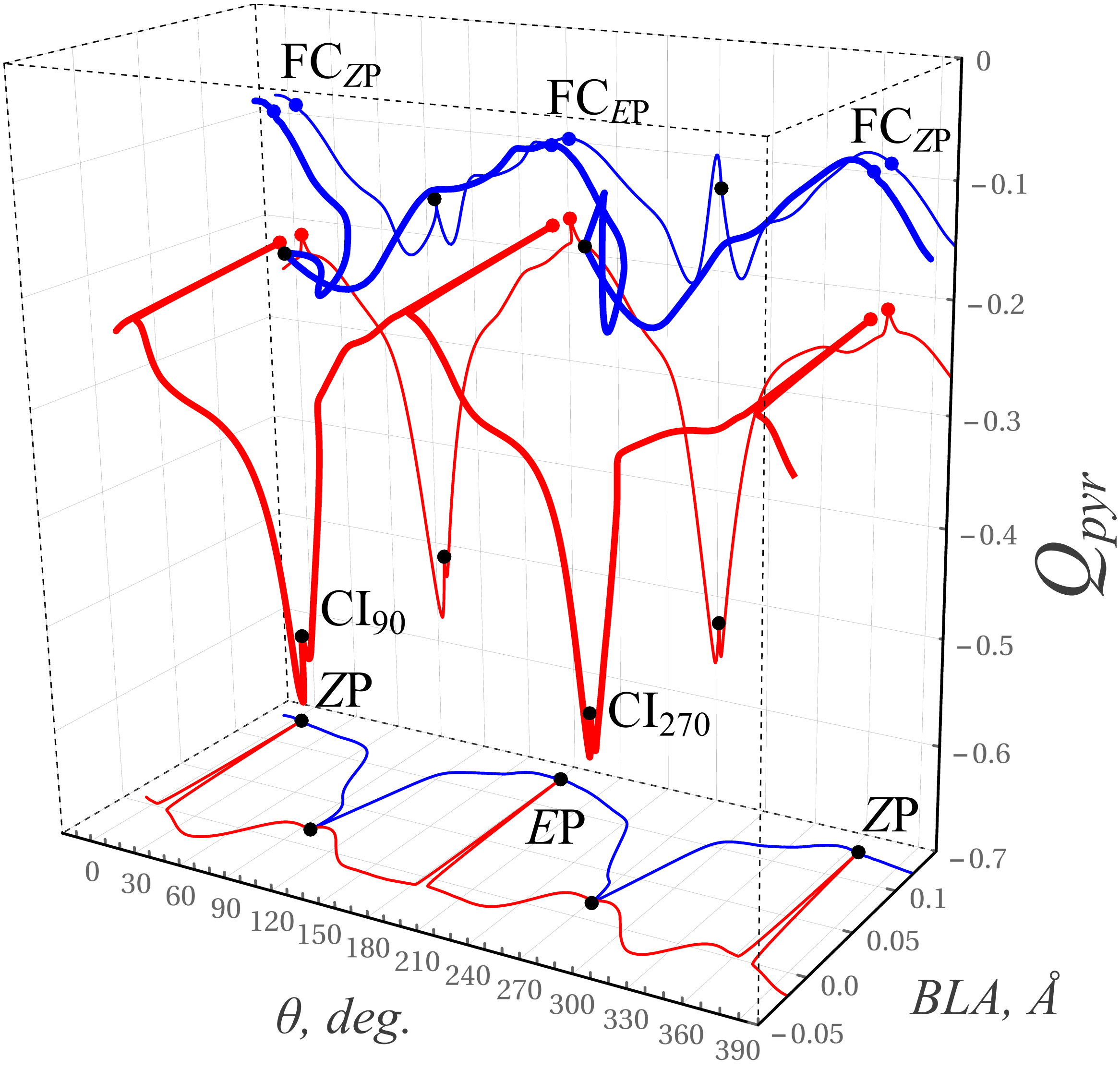

Supplementary Figure 9: Mulliken charge on the pyrrolidinone unit Q_{pyr} along the MEPs in Supplementary Figure 8. The blue line shows the charge in the S_0 state and the red line – in the S_1 state.

The occurrence of the CIs after the S_0 torsional barriers suggests that the MTDP motor may display sufficiently high quantum yield of photoisomerisation, *i.e.*, the reactions *E*P → CI_{270} → *Z*P and *Z*P → CI_{90} → *E*P should be preferred to the aborted isomerisation

reactions EP → CI_{270} → EP and ZP → CI_{90} → ZP. The MEPs in Supplementary Figure 8 display no other S_0 minima except of the EP and ZP geometries. Hence, the MTDP motor should be able to cycle between the EP and ZP configurations via photoisomerisation steps only.

Plot in Supplementary Figure 9 shows the total Mulliken charge on the pyrrolidinone unit along the MEPs in Supplementary Figure 8. The S_1 state displays more pronounced ionic characteristics, than the S_0 state. Especially, in the vicinity of the MECI geometries, the ionicity of the S_1 state increases. The greater ionicity of the S_1 state suggests that, in the polar solvents, it will be stronger stabilized than S_0, which should lead, *i.a.*, to a red-shift of its UV absorption spectrum.

4.1.4 The absence of the M diastereomers

Inspection of the MEPs in Supplementary Figure 8 reveals that, in the ground electronic state, the MTDP motor does not feature stable conformers with the M helicity. To explain the conspicuous absence of the M diastereomers a series of additional geometry optimizations have been undertaken.

For the MTDP motor, two sets of the SSR-BH&HLYP/6-31G* geometry optimizations in the ground electronic state have been carried out starting from the MEP snapshots featuring pronounced M helicity. In particular, snapshots in the middle of the CI_{90} → ZP and the CI_{270} → ZP legs of the MEP were selected, as shown in the left panels of Supplementary Figure 10.

The progress of the geometry optimization is shown in Supplementary Figure 10 as evolution of the energy (calculated relative to the most stable, *i.e.*, EP conformer of MTDP) with the optimization step. For both starting geometries, the optimization process converges initially to a geometry featuring the M helicity; shown in the insets in Supplementary Figure 10. However, the presumed ZM and EM conformers are not local minima on the ground state PES. Instead, these species are plateaux on the PES, which

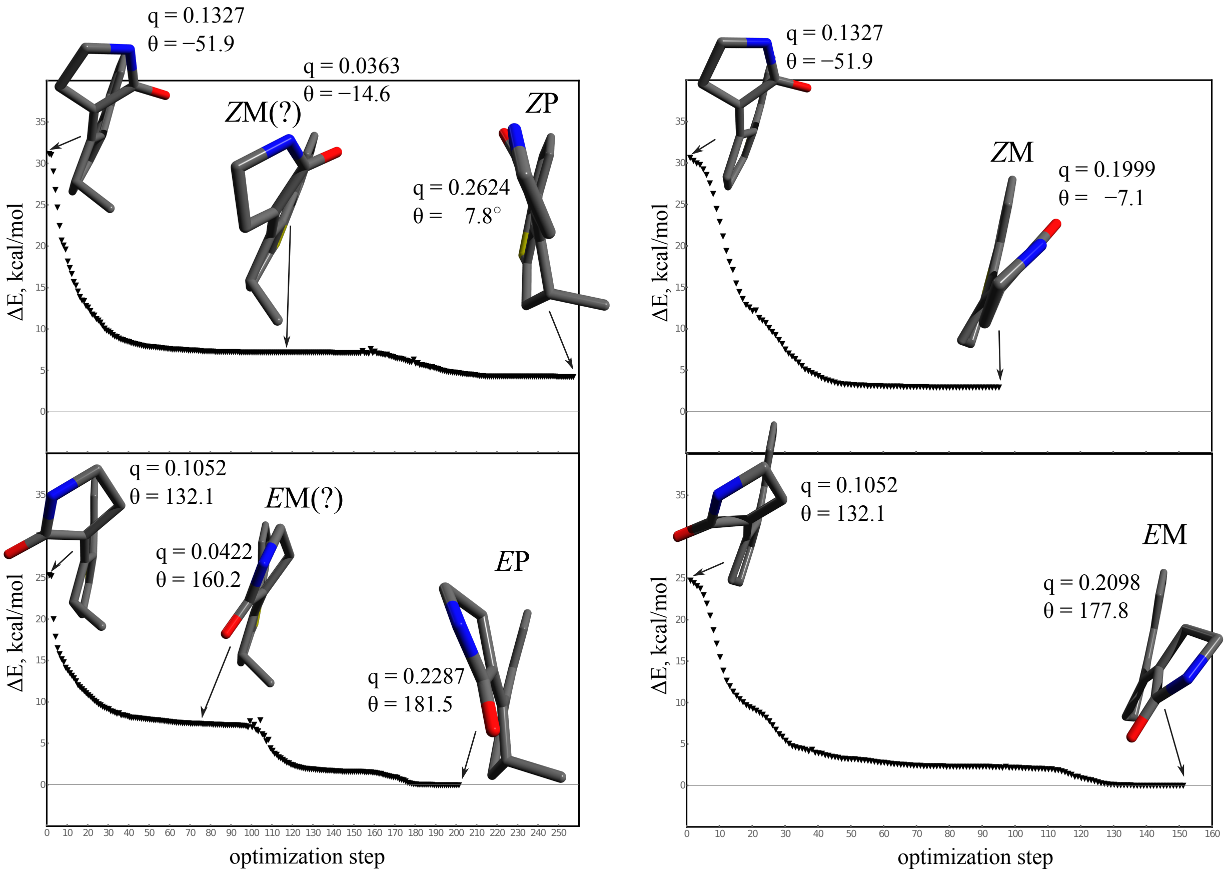

Supplementary Figure 10: Optimization of the geometry of the S_0 equilibrium geometry starting from the image #8 of the $\mathrm{CI}_{S_1/S_0} \rightarrow$ *Z*P and $\mathrm{CI}_{S_1/S_0} \rightarrow$ *E*P legs of the MEPs in Supplementary Figure 8. The left panels show the geometry optimization for MTDP, the right panels show optimization for MTDP with the exocyclic methyl group replaced by hydrogen. The energies (kcal/mol) are shown relative to the stable *E*P isomer. The puckering amplitude of the cyclopropene ring q(Å) and the central torsion angle θ (deg.) are shown for each structure displayed in the figure. The hydrogen atoms have been removed, for clarity.

converge to the stable *Z*P and *E*P minima, as the geometry optimization progresses.

For a few selected species in Supplementary Figure 10, the Cremer-Pople puckering[49] amplitude q of the cyclopentene (CPE) ring is shown alongside the central torsion angle θ. It is seen that the putative *Z*M and *E*M species feature almost planar CPE rings characterized by a very low value of the puckering amplitude q. On the other hand, the stable *E*P and *Z*P species are characterized by a pronounced puckering of the CPE ring, which, for the *E*P diastereoisomer, agrees excellently with the corresponding

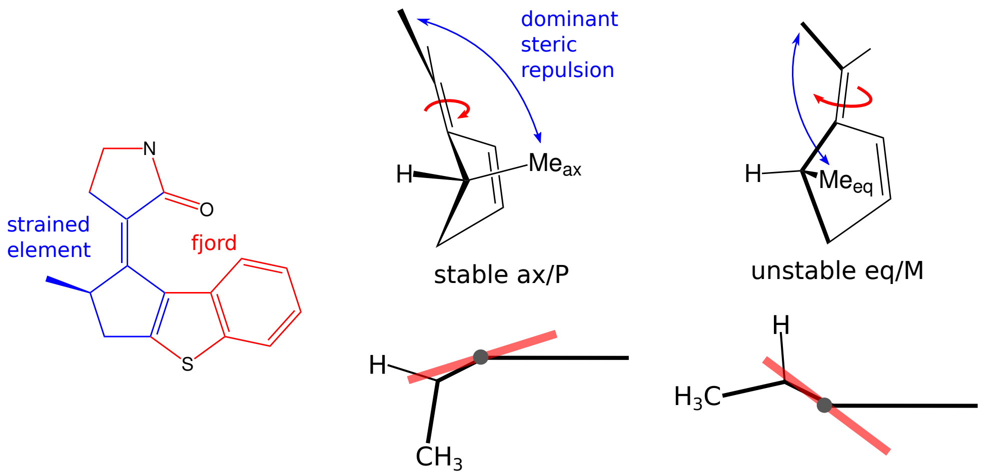

Supplementary Figure 11: Illustration of the factors contributing to absence of the stable M helicity (or helical conformation) of MTDP. The *EP* and *ZP* configurations feature axial (ax) orientation of the exacyclic Me group, whereas the putative *EM* and *ZM* configurations should favour equatorial (eq) orientation. Due to the steric repulsion with the rotor unit (pyrrolidinone) the M helical conformations would require an increased torsion of the central double bond (rotation axis) and this destabilizes the corresponding configurations.

experimentally determined geometry.

It can be hypothesized that the CPE ring puckering is hindered in the M helical species, which therefore cannot become stable local minima and remain transient species on the flat PES. To identify the factor behind inhibiting the stable M helical conformations, an additional set of geometry optimizations have been performed for the MTDP motor modified by substituting the exocyclic Me group by hydrogen atom. The resulting compound **4** loses chirality at the C_7 atom. Hence, the M and P helical species become equivalent.

As seen in the right panels of Supplementary Figure 10, the geometry optimizations of **4** initiated at the same geometries as in the right panels converge to stable *ZM* and *EM* local minima, which are energetically equivalent to the *ZP* and *EP* configurations; geometrically the latter are the mirror reflections of the former and are not shown in Supplementary Figure 10. Both the *ZM* and *EM* (as well as the *ZP* and *EP*) diastereoisomer of **4** are characterized by a pronounced puckering of the CPE ring, which is similar to the stable *ZP* and *EP* diastereoisomers of MTDP (**2**). The M and P

local minima of **4** are separated by shallow transition states occurring at 1.1 kcal/mol (*E*M → *E*P) and 1.5 kcal/mol (ZM → *Z*P), see Supplementary Figure 12. This indicates that the steric strain in the fjord region of **4** (as well as **2**) is rather low.

Hence, it is the presence of the exocyclic Me group in MTDP that hinders the M helical species from being stable local minima. Indeed, as shown in Scheme 11, the M helical species should feature puckered CPE ring with the Me group attaining the equatorial position. For the P helical species, the axial orientation of the Me group is preferred. This observation is consistent with the geometry optimization of **4**, where one can replace Me with a labelled H atom (H*), as well as the experimental observations.[50]

It is noteworthy that planarisation of the central double bond, *i.e.*, bringing the central dihedral angle θ closer to 0° or 180°, should result in increase of the steric repulsion between the exocyclic Me group and pyrrolidinone blade, when the Me group attains the equatorial position. In the case of the axial Me group, planarisation of the central double bond leads to a reduction of the steric repulsion, see Supplementary Figure 11. Hence, it can be assumed that it is the rigidity of the double bond to torsion that destabilizes the M helical conformations of MTDP.

To evaluate whether the above supposition can be true, a number of additional species derived from MTDP were investigated. In the compound **5**, the pyrrolidinone blade is replaced by carbene (CH_2). This essentially eliminates the steric repulsion between the stator and the rotor (CH_2) lobes of **5** and removes hindrance for attaining the equatorial orientation of Me. Indeed, as seen in Supplementary Figure 12, both Me_{eq} and Me_{ax} conformations become stable local minima.

It is however noteworthy that the two local minima are separated by a very shallow transition state; only 0.04 kcal/mol higher than the meta-stable Me_{eq} minimum. This can be traced back to the effect of π-conjugation between the exocyclic double bond and the double bond inside the CPE ring; which should favour a more planar CPE ring. When the conjugation is broken by removing the CH_2 group and saturating the C_6 atom with two

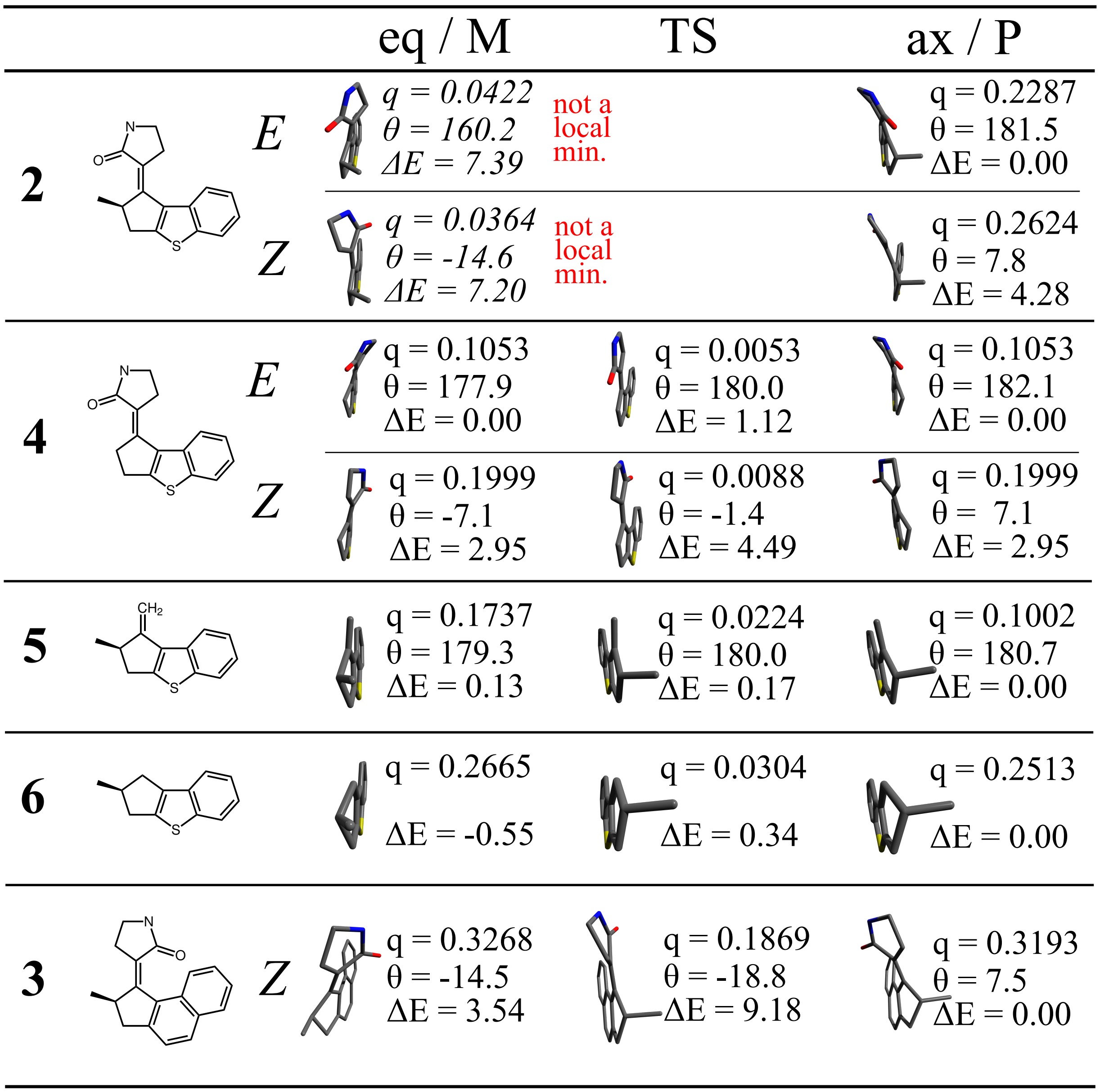

Supplementary Figure 12: Optimized geometries of the ground state stationary points of the species derived from the MTDP motor (**2**). The preferred orientation of the exocyclic Me group is also given for each helicity. The local minima are shown in the column labelled "eq / M" and "ax / P" and the transition states between the local minima are shown in the "TS" column. For the *Z*M and *E*M configurations of MTDP (**2**), it is specifically given that these are not local minima, but transient species on the flat segment of the respective PES, and their characteristics are shown in italicized font.

hydrogens, the resulting compound **6** features a more pronounced CPE ring puckering than **5** and a taller barrier to inversion. It is plausible therefore that the π-conjugation

leads to a certain planarisation of the CPE ring of **5**, which features a somewhat lower puckering amplitude than the other species in Supplementary Figure 12.

Hence, it can be concluded that the absence of the stable M helical conformations of MTDP is caused by the trade-off between the CPE ring puckering and the tendency of the central double bond to attain maximally planar local geometry. As planarisation of the central double bond in the M helical conformations, which simultaneously feature the Me group in the equatorial position, leads to a certain increase in the steric repulsion between the Me group and the pyrrolidinone ring, the putative *Z*M and *E*M configurations are destabilized and occur only as shoulders on the ground state PES.

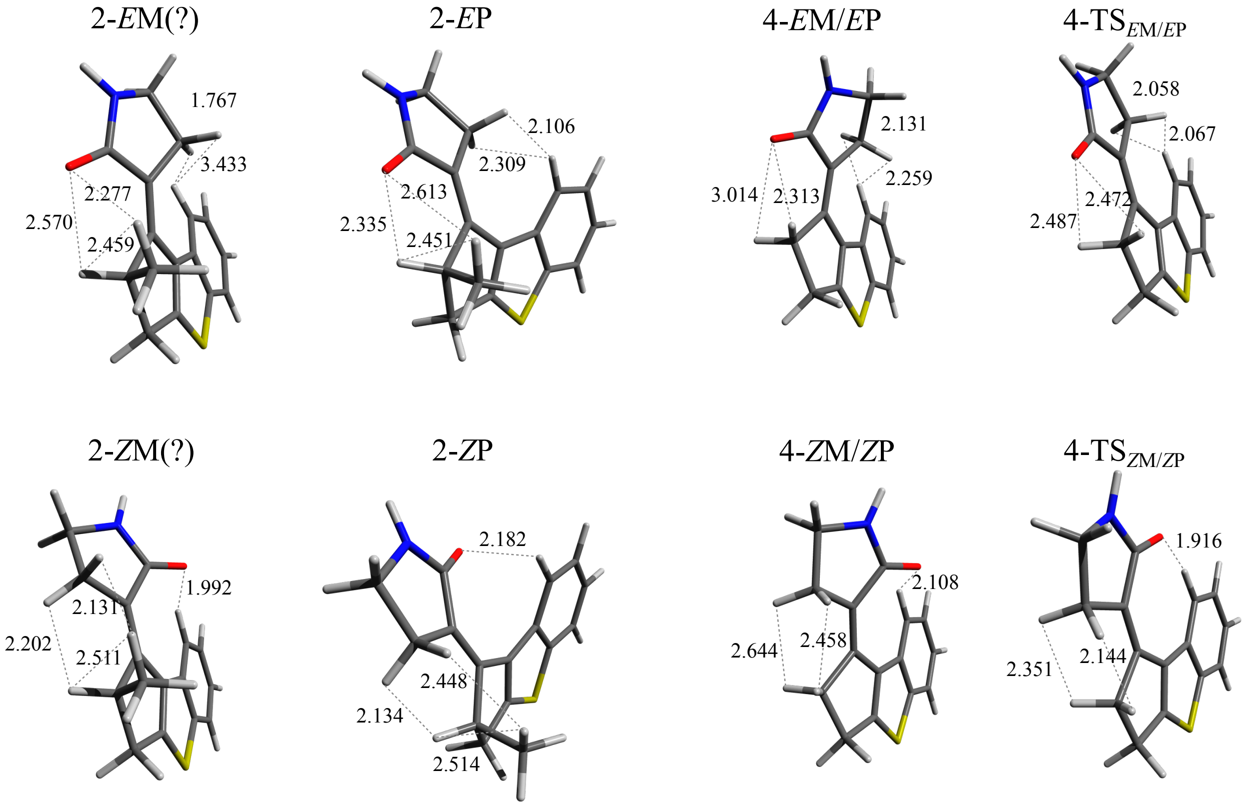

Supplementary Figure 13: Optimized geometries of the ground state stationary points of MTDP (**2**) and demethylated MTDP (**4**). The key bondlengths are shown. The query mark at the *E*M and *Z*M MTDP species denotes that these are transient species on the S_0 PES (see Supplementary Figure 10), rather than stable local minima.

In Supplementary Figure 12, it is seen that the barrier to the "eq / M" → "ax / P" inversion in the demethylated MTDP (**4**) is rather low; only 1.1–1.5 kcal/mol. This can

be explained by a relatively long distances between the exocyclic oxygen and hydrogen atoms of pyrrolidinone and the hydrogen atoms of the CPE and benzene rings of the stator; see Supplementary Figure 13. Supplementary Figure 13 also illustrates that the origin of the instability of the *E*M/*Z*M configurations of MTDP is indeed the proximity of the exocyclic Me group: At the unstable geometries, the distances between the pyrrolidinone atoms and the Me group are noticeably shorter than in the stable *E*P and *Z*P diastereoisomers. This should result to stronger steric repulsion with the Me group and in greater torsional strain on the central double bond; which is also illustrated by the central dihedral angle θ in Supplementary Figure 10.

4.1.5 Vertical excitation energies and absorption spectra of *E*P and *Z*P configurations

At the S_0 equilibrium geometries of the *E*P and *Z*P configurations (optimized by the SSR-BH&HLYP/6-31G* method), the VEEs were calculated by both the SSR method and the TD-BH&HLYP/6-31G* method, see Supplementary Table 3. There is a good agreement between the VEEs obtained by both methods for the optically bright S_1 state; the latter state corresponds to a $\pi \rightarrow \pi^*$ one-electron transition centred around the C_6=$C_{3'}$ double bond and has a large oscillator strength. The S_2 to S_4 states have charge transfer character and involve transitions from the lone pair orbitals of the O atom (S_2) and the S atom (S_3) to the π^* orbital of the benzene ring. These excited states lie *ca.* 0.4–0.7 eV above the S_1 state and have low oscillator strengths. The only other excited state with a relatively large oscillator strength (S_6, not reported in Supplementary Table 3) lies at 5.81 eV and corresponds to one-electron transition between a π orbital centred on the benzene ring to the π^* orbital centred around the C_6=$C_{3'}$ bond. This leaves the S_1 state the only optically bright state populated by photoexcitation within UVA/UVB region. This is a photochemically active state leading to breaking the π component of the central C_6=$C_{3'}$ double bond.

The UV absorption spectra of the *E*P and *Z*P conformers were simulated

Supplementary Table 3: Vertical excitation energies (eV) of the ground state equilibrium configurations of MTDP motor obtained in the SSR-BH&HLYP/6-31G* and TD-BH&HLYP/6-31G* calculations. Oscillator strengths of the transitions are given in parentheses.

	SSR	TD-DFT			
configuration	$S_1 \leftarrow S_0$	$S_1 \leftarrow S_0$	$S_2 \leftarrow S_0$	$S_3 \leftarrow S_0$	$S_4 \leftarrow S_0$
*E*P	4.76 (0.52)	4.73 (0.45)	5.08 (0.10)	5.15 (0.00)	5.26 (0.07)
*Z*P	4.63 (0.50)	4.59 (0.44)	5.09 (0.02)	5.14 (0.01)	5.38 (0.19)

theoretically[51] by convolution of the vertical excitations calculated by the SSR-BH&HLYP/6-31G* method at one hundred geometries obtained from sampling the Wigner function of the respective conformer at T=300K. The Lorentzian lineshape $L(\lambda; \lambda_i, f_{\lambda_i}) = f_{\lambda_i} \frac{\sigma^2}{\sigma^2 + (\lambda - \lambda_i)}$, centred around the calculated excitation wavelengths λ_i with an amplitude given by the respective oscillator strength f_{λ_i} and broadened by a uniform width σ = 10 nm was used to simulate absorption spectra of the lowest (S_1) absorption band. The resulting spectra are shown in the left panel of Supplementary Figure 14, the *E*P diastereoisomer by the blue curve and the *Z*P diastereoisomer by the red curve. The absorption maxima of both diastereoisomers are very close, 273 nm (*E*P) and 275 nm (*Z*P). Extension of the basis set to 6-311G**, *i.e.*, the SSR-BH&HLYP/6-311G** method, leads to a slight red shift of the absorption maxima, 278 nm (*E*P) and 280 nm (*Z*P).

As the shape of the first absorption band is different for the two diastereoisomers, the difference of the absorptivity of the two diastereoisomers can, in principle, be used to determine their relative concentration in a mix of the conformers. The right panel of Supplementary Figure 14 shows the difference of the absorbances A(*Z*P) - A(*E*P), which represents theoretical model of a stationary difference spectrum resulting from the complete (100%) conversion of the *E*P diastereoisomer to *Z*P. If the *E*P *to* *Z*P conversion is incomplete and a stationary mix of the two conformers is formed at the end of the photoreaction, the spectrum in the right panel of Supplementary Figure 14 has to be scaled by a factor $c \leqslant 1$, *i.e.*, the fraction of the *Z*P diastereoisomer in the mix. Thus, the knowledge of the differential spectra of the *E*P/*Z*P mixtures with varying composition

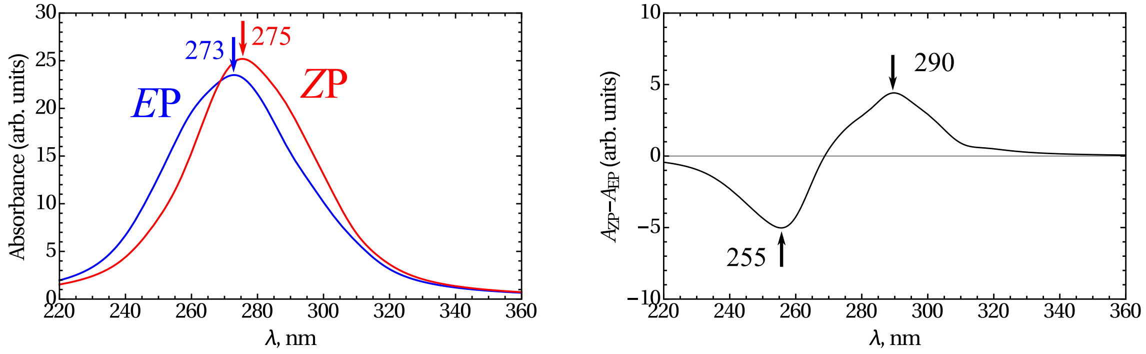

Supplementary Figure 14: Theoretical UV/vis absorption spectra of **2** estimated by the SSR-BH&HLYP/6-31G* method. Left panel: The absorption spectra of the *E*P conformer (blue curve) and for the *Z*P conformer (red curve). Right panel: The difference absorbance A(ZP) - A(*E*P) of the *Z*P and *E*P conformers. Arrows show positions of the respective maxima (minima).

can provide facilities for the determination of the quantum yield of the photoreaction.

The gas phase theoretical UV/Vis spectrum in Supplementary Figure 14 bears some semblance to the experimental spectrum of the *E*P conformer in Supplementary Figure 30. The theoretical absorption maximum is blue shifted by *ca.* 40 nm (from $\sim$ 310-315nm (exp.) to 273nm (theor.)), which can be attributed mostly to the effect of the solvent, as well as to some imperfectness of the computation, such as the use of the approximate density functional and an incomplete basis set. Nevertheless, assuming a constant shift of the theoretical spectrum with respect to the experiment, there is a reasonable agreement with the experimental stationary difference spectrum in Supplementary Figure 30B. The amplitude of the difference spectrum depends linearly on the fraction of the *Z*P isomer in the final (stationary) mix of the reaction products.

4.2 Non-adiabatic molecular dynamics simulations in isolated conditions

For the *E*P configuration 50 trajectories were prepared and ran using the DISH-XF/SSR-BH&HLYP/6-31G* method for 1200 steps with the time step of 0.48fs (576fs), see Section

3 for detail. Starting in the S_1 state with the initial geometries and velocities generated by sampling the Wigner function 46 trajectories underwent non-adiabatic transition to the S_0 state and four trajectories remained in the S_1 state until the end of the simulation. For the *Z*P conformer, 45 trajectories were prepared and ran for 900 steps (432fs). Out of 45 all but one trajectory underwent transition to the S_0 state.

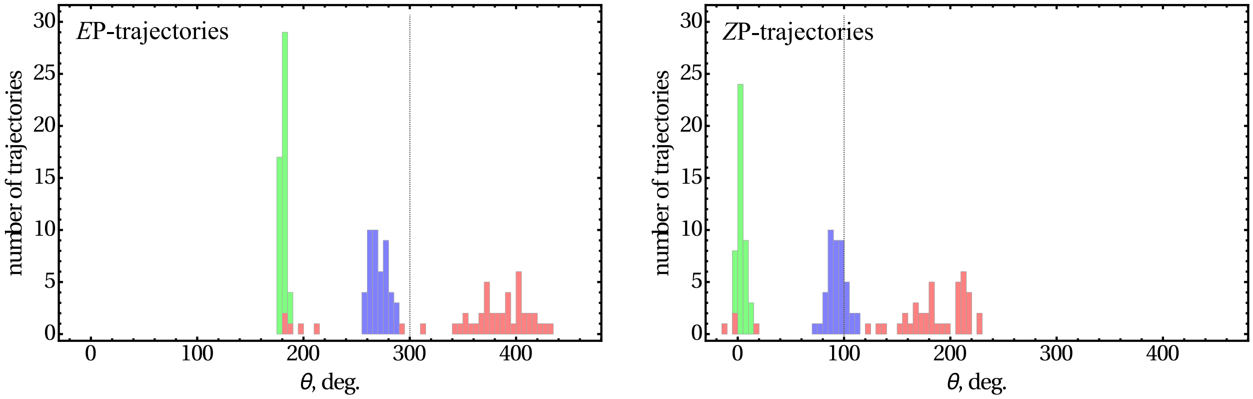

Supplementary Figure 15: Distributions of the central dihedral angle θ (in deg.) at the beginning of the trajectories (green), at the time of surface hop (blue), and at the end of the trajectories (red). Vertical dotted lines show the cut-off θ value used to discriminate between the reactants (to the left of the line) and products (to the right of the line).

To evaluate the results of the NAMD simulations distribution of the central dihedral angle θ was inspected. The distributions of the angle θ are shown in Supplementary Figure 15, where the green bars show angles at the beginning of th respective trajectories, blue bars at the time of $S_1 \rightarrow S_0$ hop, and red bars at the end of the trajectories. To discriminate between the reaction products, the limiting values of the angle θ were set; for discrimination between *E*P and *Z*P conformers in the *E*P trajectories $\theta = 300°$ was used and for the *Z*P trajectories $theta = 100°$. The *E*P trajectories propagate starting with the θ values in the vicinity of 180° towards greater values of θ ($\theta \gtrapprox 270°$). The *Z*P trajectories start at $\theta \approx 0°$ and propagate towards θ values $\gtrapprox 90°$.

A more detailed view of the variation of the key geometric parameters, θ and BLA, along the trajectories is given in Supplementary Figure 16. As seen in Figs. 15 and 16, the trajectories undergo the $S_1 \rightarrow S_0$ transition in a narrow range of angles which match

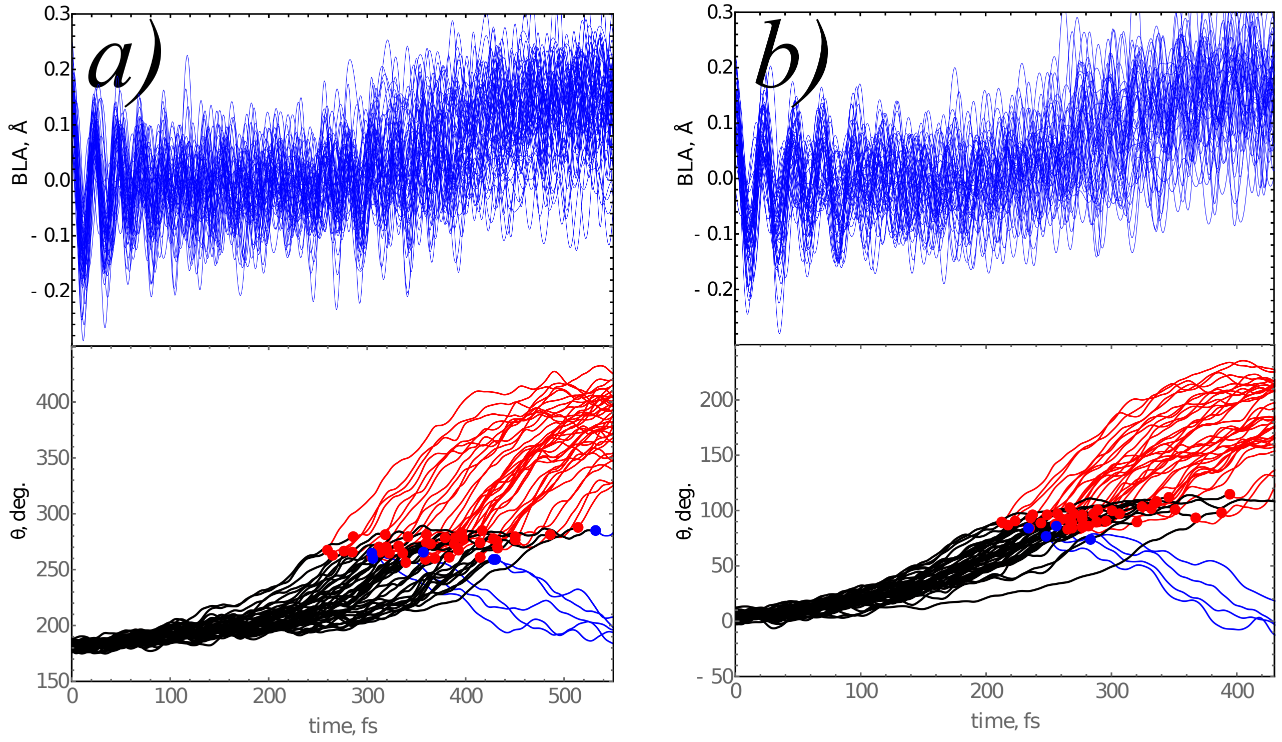

Supplementary Figure 16: Dihedral angle θ (deg.) and the BLA distortion (Å) as functions of the propagation time (fs) along the NAMD trajectories initiated in the *E*P configuration (a) and in the *Z*P configuration (b). The red dots show the $S_1 \rightarrow S_0$ surface hops for the trajectories propagating in the direction of isomerisation (to the *Z*P and *E*P configurations, respectively) and the blue dots show the surface hops for the trajectories turning back to the initial configuration.

the geometry of the CI_{270} for the *E*P trajectories and CI_{90} for the *Z*P trajectories. This direction of the propagation corresponds to the CCW rotation of the pyrrolidinone blade. No trajectories rotating in the opposite, CW, direction were observed. This result was also confirmed by visual inspection of the trajectories. At the beginning of both the *E*P and the *Z*P trajectories, concerted oscillation of the BLA distortion is seen in Supplementary Figure 16. This suggests the presence of coherent vibrational motions, which can be possibly detected by the ultrafast Raman spectroscopy or the ultrafast fluorescence spectroscopy. After *ca.* 100fs, the coherency in the BLA oscillations disappears.

Supplementary Table 4: Parameters (μ and σ) of the Gaussian fit of the $S_1 \to S_0$ hop times, parameters (τ and t_0) of the exponential fit of the S_1 populations, S_1 lifetimes (τ_{S_1}), and quantum yield of isomerisation (ϕ) for the photoisomerisation reactions started in the *E*P and *Z*P configurations of the MTDP motor. The error estimates were obtained by the bootstrap resampling[52] (10^4 bootstrap cycles) of the obtained samples. The average of the samples is reported outside parentheses and the standard deviation is given parenthetically.

	Gaussian fit[a]		exponential fit[b]			
reaction	μ, fs	σ, fs	τ, fs	t_0, fs	τ_{S_1},[c] fs	ϕ[d]
*E*P → *Z*P	372(9)	63(6)	133(13)	280(9)	413(13)	0.87(0.05)
*Z*P → *E*P	281(7)	44(5)	75(9)	221(5)	298(9)	0.91(0.04)

[a] Gaussian fit $g(t) = \frac{1}{\sigma\sqrt{2\pi}} e^{-\frac{(t-\mu)^2}{2\sigma^2}}$ of the $S_1 \to S_0$ hop time distribution. μ and σ are the mean value and the square root of the variance, respectively. The μ and σ parameters were calculated for each bootstrap replica of the initial sample and averaged over all bootstrap samples.
[b] Exponential fit $f(t) = e^{-(t-t_0)/\tau}$ of the S_1 populations. For each bootstrap sample, t_0 and τ were calculated individually and then averaged over all bootstrap samples.
[c] The S_1 state lifetime obtained for each bootstrap sample as $\tau_{S_1} = t_0 + \tau$ and averaged over all bootstrap cycles.
[d] The photoisomerisation quantum yield obtained for each bootstrap sample as a ratio of the number of isomerised trajectories to the total number of trajectories; averaged over all the bootstrap samples.

The parameters of the *E*P and *Z*P photoreactions were obtained by averaging over

all the trajectories that underwent the $S_1 \to S_0$ transition and the error estimates were obtained using the bootstrap resampling technique.[52] The S_1 and S_0 populations were obtained as a fraction of the trajectories residing in the respective electronic state at the given instance of time. The obtained S_1 population dynamics is shown in Supplementary Figure 17. The dependence of the S_1 population on time was fitted by a monoexponential function, see Supplementary Table 4. Distribution of the $S_1 \to S_0$ hop times was fitted by a Gaussian distribution function. The parameters of the distributions and the S_1 lifetime (obtained by the condition $n(t) = \exp(-1)$) are reported in Supplementary Table 4. The isomerisation quantum yields $\phi_{EP \to ZM}$ and $\phi_{ZP \to EM}$ were calculated as the ratio of the number of trajectories successfully isomerised to the total number of trajectories, see Supplementary Table 4.

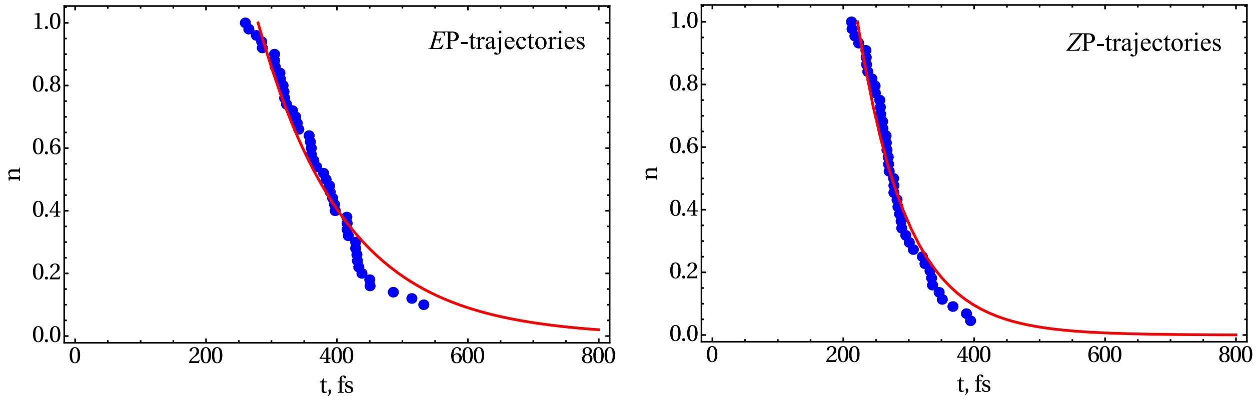

Supplementary Figure 17: Population (n) of the S_1 state as a function of the propagation time t (in fs). The S_1 populations are shown by blue dots and the exponential fits are shown by the red curves. See Supplementary Table 4 for the parameters of the exponential decay.

The MTDP motor shows an ultrafast dynamics of photoisomerisation with the lifetimes τ_{S_1} on the order of 300–400 fs, see Supplementary Table 4. Both photoisomerisation steps propagate in the same, CCW, direction of torsion. As suggested by the adiabatic MEPs in Supplementary Figure 8, the trajectories, if continued to longer times and allowed to dissipate the excess of the vibrational energy, should settle in the configurations with the P helicity, *i.e.*, *E*P or *Z*P. Hence, the adiabatic MEPs and the

results of the NAMD simulations show unambiguously that the MTDP motor should be capable of performing a full 360° revolution by absorbing photons only; no thermally activated helix inversion step is required.

Both photoisomerisation steps of MTDP display sufficiently high isomerisation quantum yields, 0.87±0.05 for the *E*P → *Z*P step and 0.91±0.04 for the *Z*P → *E*P step. However, no selection of the initial conditions, *e.g.*, to match a specific excitation wavelength, has been made when setting up the NAMD simulations. This is somewhat different to what is usually done during the ultra-fast spectroscopy experiments. When selecting only the *E*P and *Z*P trajectories with the initial excitation wavelength of 270nm, the theoretical isosbestic point in Supplementary Figure 14, the respective quantum yields become 0.73±0.12 (*E*P) and 0.67±0.27 (ZP). Note however that the number of trajectories in the 270±5nm excitation wavelength interval is rather small, 14 for *E*P and 3 for *Z*P, which does not enable statistically significant determination of the quantum yield. This notwithstanding, it can be conjectured that a lower quantum yield should be observed under the experimental conditions. Yet another factor reducing the isomerisation quantum yield is the effect of the solvent, which is missing in the gas phase theoretical simulations. As the photoreaction occurs on an ultra-fast timescale, the solvation shell around the molecule cannot adjust to the new molecular configuration rapidly enough. Hence, die to the resilience of the solvation shell, there may be a considerable bias towards the initial configuration, which can further reduce the observed quantum yield.

4.3 Non-adiabatic molecular dynamics simulations in solution

In the following we describe the preparation of the solvent (MeOH) environment for the adiabatic path optimization and NAMD simulations using a suitable hybrid quantum mechanics / molecular mechanics (QM/MM) model. The protocol starts with the construction of an isolated MTDP molecule that is then optimized at the MP2 level[53,54] with 6-31G* basis set[35] (MP2/6-31G*) using an implicit methanol solvent under the Polarisable Continuum Model (PCM).[55] The calculations were performed employing the GAUSSIAN computer package.[56] The optimized MTDP molecule was then embedded in the centre of a cubic box of the size 60 x 60 x 60 Å containing 2947 MeOH molecules. The energy of the solvent system was then minimized at the molecular mechanics (MM) level using the conjugated-gradient method with periodic boundary conditions in the presence of a geometrically frozen MTDP molecule. During this process the assigned atomic charges of the MTDP atoms were calculated using the Second Order Electrostatic Potential Fitting (ESPF) method starting from the MP2/6-31G* data). Instead, the bonding and van der Waals parameters were taken from the OPLS-AA force field.[57] The above energy minimized MTDP-solvent system was then relaxed via a 5000 ps long molecular dynamics (MD) simulation under the isothermal-isobaric ensemble (NPT) set at 1 atm and 298 K using the GROMACS software package.[58] During the first 300 ps of the simulation, the system was gradually thermalised to 298 K, equilibrated for the next 700 ps and sampled for the next 4000 ps. From the latter 4000 ps dynamic simulation step (i.e. the ”production” step), 400 uncorrelated snapshots were extracted every 10 ps and assumed to be representative of the thermal equilibrium ensemble. Each snapshot comprised a 20 Å solvent sphere (906 MeOH molecules) used in the construction of the QM/MM models. In the following computations, a frozen boundary approach[59,60] was used to keep the distribution of MeOH molecules fixed within the boundary layer. In the computations, the inner part of the solvent "blob" and the solute molecule were allowed to move, whereas the outer part, the size of which could vary for different initial

condition/trajectory, was kept fixed.

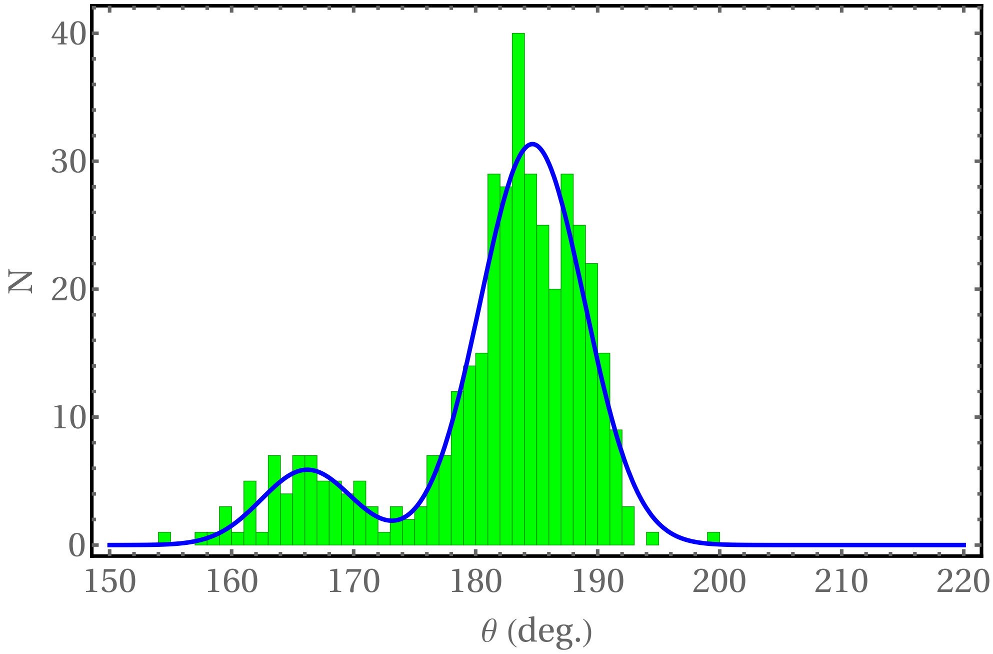

Supplementary Figure 18: Distribution of occurrence of the central dihedral angle θ in the initial conditions generated for the NAMD simulations of the *E*P conformation. The blue line shows a bi-Gaussian fit of the distribution.

Supplementary Figure 18 shows the distribution of the central dihedral angle θ (see 1 of the main text for definition of θ) in the initial conditions generated for the *E* diastereoisomer of MTDP. It is seen that θ follows a bimodal distribution $C_1 \cdot \frac{1}{\sigma_1 \sqrt{2\pi}} e^{-\frac{(\theta-\theta_1)^2}{2\sigma_1^2}} + C_2 \cdot \frac{1}{\sigma_2 \sqrt{2\pi}} e^{-\frac{(\theta-\theta_2)^2}{2\sigma_2^2}}$ where C_1 = 0.86, θ_1 = 184.7°, σ_1 = 4.3°, C_2 = 0.14, θ_2 = 166.2°, and σ_2 = 3.8°. Therefore, the majority of the initial conditions (86%) correspond to the *E*P configuration of MTDP, where the angle $\theta \geq 180°$. A minor part (14%) is in the *E*M configuration, which is unstable, both in the gas phase and, arguably, in solvent; see below. However, the instantaneous snapshots from the classical MD trajectories may have the M helicity. This is one of the manifestations of the solvent influence on the molecule and, obviously, also represents an additional limitation (L4, see main text for L1-L3) to the the function of a unidirectional light-driven molecular motor that we do not discuss in the main text. The presence of a second minor maxima in the distribution in Supplementary Figure 18 does not imply the existence of an energy minimum on

the S_0 PES corresponding to a stable *E*M diastereoisomer. In fact, a shoulder on the S_0 MEP visible in Supplementary Figure 8-10 (gas-phase) and Supplementary Figure 22 (solution phase) and corresponding to the torsion angle values typical for *E*M, is held responsible for the population of metastable *E*M configurations. In other words, the minor "maximum" in the distribution of the initial configurations in Supplementary Figure 18 could be interpreted in terms of the anharmonicity of the S_0 PES.

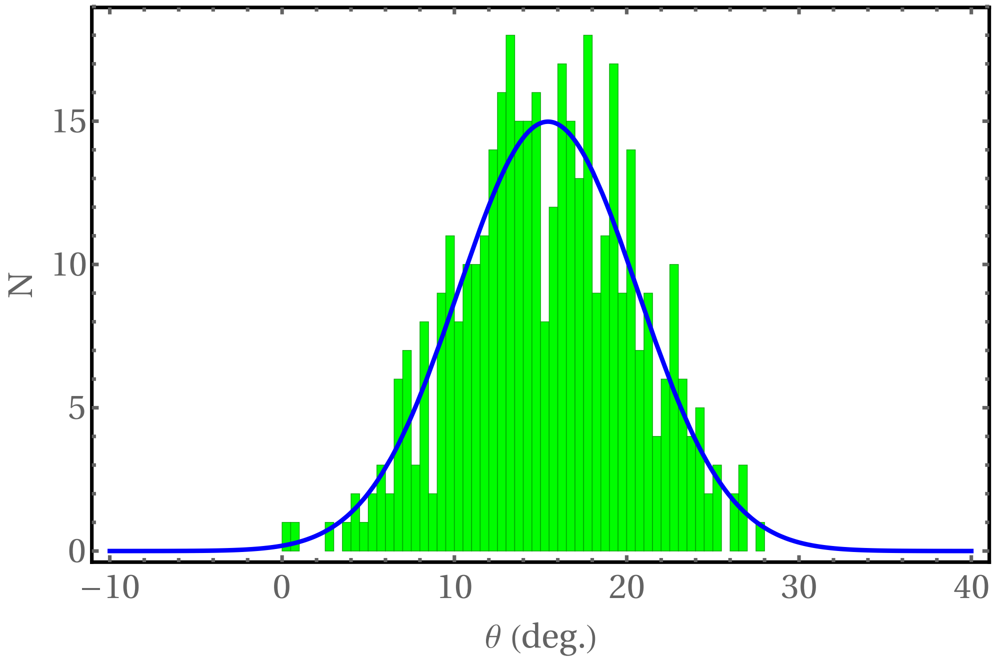

Supplementary Figure 19: Distribution of occurrence of the central dihedral angle θ in the initial conditions generated for the NAMD simulations of the *Z*P conformation. The blue line shows a Gaussian fit of the distribution.

The initial conditions for the *Z*P conformation have been prepared in the same way as for *E*P. The distribution of the dihedral angle θ in the initial geometries of *Z*P is shown in Supplementary Figure 19. The value of θ in *Z*P follows a unimodal distribution centred around a value of 15.4° with a dispersion of 5.2°. The expectation value of θ corresponds to the *Z*P conformation (*Z*M would have a negative value of θ). This implies that the *Z*P conformer is considerably less affected by the solvent and does not display even transient structures with the M helicity.

4.3.1 Stationary Points and Adiabatic Minimum Energy Paths

Before running NAMD trajectories, we have looked at the main features of the PESs of the solvated MTDP. The mapping has been performed with the MTDP solute described at the SSR(2,2)/BH&HLYP/6-31G* level and therefore at the same QM level used to study the gas-phase (i.e. isolated) chromophore. The surrounding solvent "blob" includes 906 MM methanol molecules (see Supplementary Figure 20A), whose initial configuration is taken from one of the snapshots of the ground state MD simulations described above, is treated using the OPLS-AA force field and whose. During all geometry optimizations (minima, conical intersections and MEPs), all the solvent molecules but 49 were kept fixed. The 49 CH_3OH molecules form the immediate solvation shell of MTDP shown in Supplementary Figure 20B. The geometries and the positions of the CH_3OH molecules in the immediate solvation shell have been optimized using TINKER, whereas the geometry of the solute was optimized at the QM/MM level by using GAMESS-US interfaced with TINKER.[61]

In Supplementary Figure 21, we display the S_0 minima of the *E*P and *Z*P diastereoisomer in methanol superimposed with the corresponding gas-phase structures. The effect of the methanol environment on the geometry is relatively weak; the dihedral angle θ deviates from the gas phase values by $\lesssim 1^{\circ}$.

The geometries of the conical intersections were optimized using the CIOpt code[40]. The QM/MM computations of the gradient and the molecular energy was performed, again, with GAMESS-US interfaced with TINKER. The positions and the geometries of 49 methanol molecules in the MTDP solvation shell as well as the geometry of MTDP were updated by the CIOpt program; the positions and geometries of the remaining methanol molecules were kept fixed. The CIs obtained in the QM/MM optimizations are superimposed in Supplementary Figure 21 with the respective gas phase geometries.

Supplementary Table 5 compares the relative energies (with respect to the ground state *E*P conformation) and some of the geometric characteristics of the species optimized in the methanol environment and in the gas-phase. In the selected solvent "blob", the

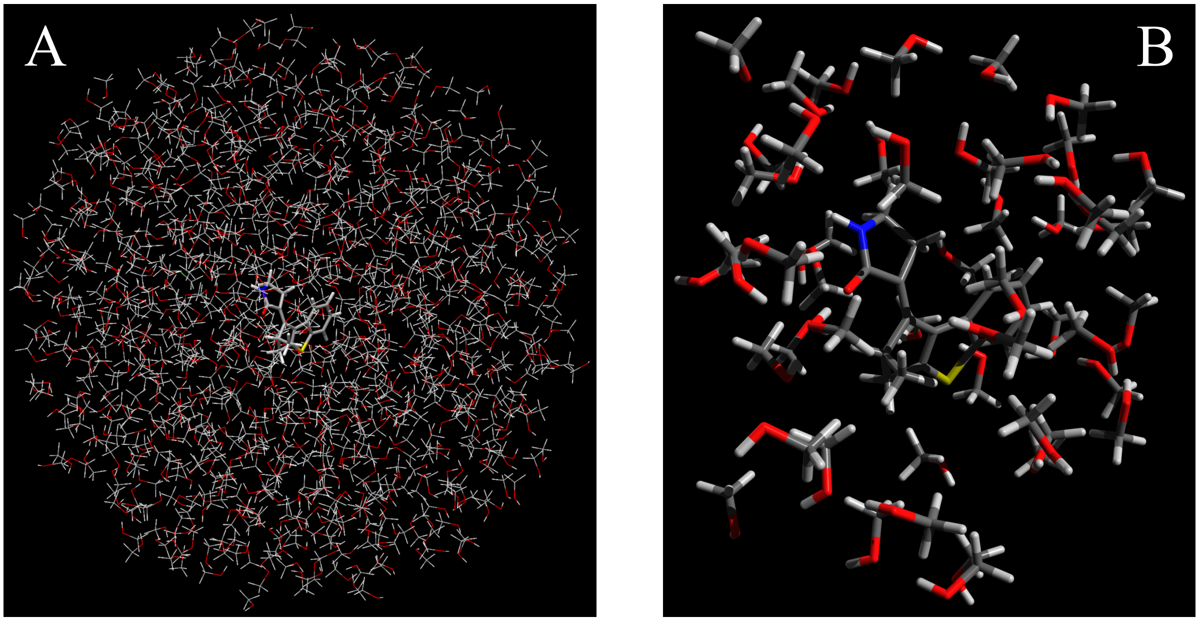

Supplementary Figure 20: The solvent box (the frozen boundary conditions) used in the QM/MM computations. Panel A shows a full box of 906 methanol molecules and MTDP. Panel B shows the solvation shell (49 CH_3OH + MTDP) optimized during the MTDP geometry optimizations and the MEP optimization.

Supplementary Table 5: Comparison of the relative energies ΔE (in kcal/mol) of the S_0 and S_1 states, the values of the central dihedral angle θ (in deg.), the bond length alternation (BLA, in Å), and the $C_{2'}$–$O_{6'}$ bondlength at the stationary geometries of the MTDP motor **2** obtained in the methanol solution and in the gas-phase. The latter values are given parenthetically.

configuration	ΔE(S_0)	ΔE(S_1)	θ	BLA	R($C_{2'}$–$O_{6'}$)
*E*P	0 (0)	110.4 (109.7)	180.8 (181.1)	0.136 (0.133)	1.223 (1.213)
*Z*P	13.8 (4.0)	123.3 (110.7)	6.2 (4.9)	0.136 (0.130)	1.214 (1.210)
CI_{90}	77.1 (72.0)	77.2 (72.0)	96.7 (95.5)	0.018 (0.016)	1.231 (1.228)
CI_{270}	79.9 (72.1)	79.9 (72.1)	267.8 (271.1)	0.010 (0.016)	1.240 (1.227)

*Z*P structure is somewhat destabilized (relative to *E*P) compared to the gas phase. In our model such destabilization includes contribution of the H-bonds between the solute and nearby methanol molecules. The energies of the CIs in the methanol environment are also slightly higher than in the gas phase. The effect of the H-bond in the *E*P conformer is also seen in a slightly longer C=O bond. The remaining geometric parameters in methanol are

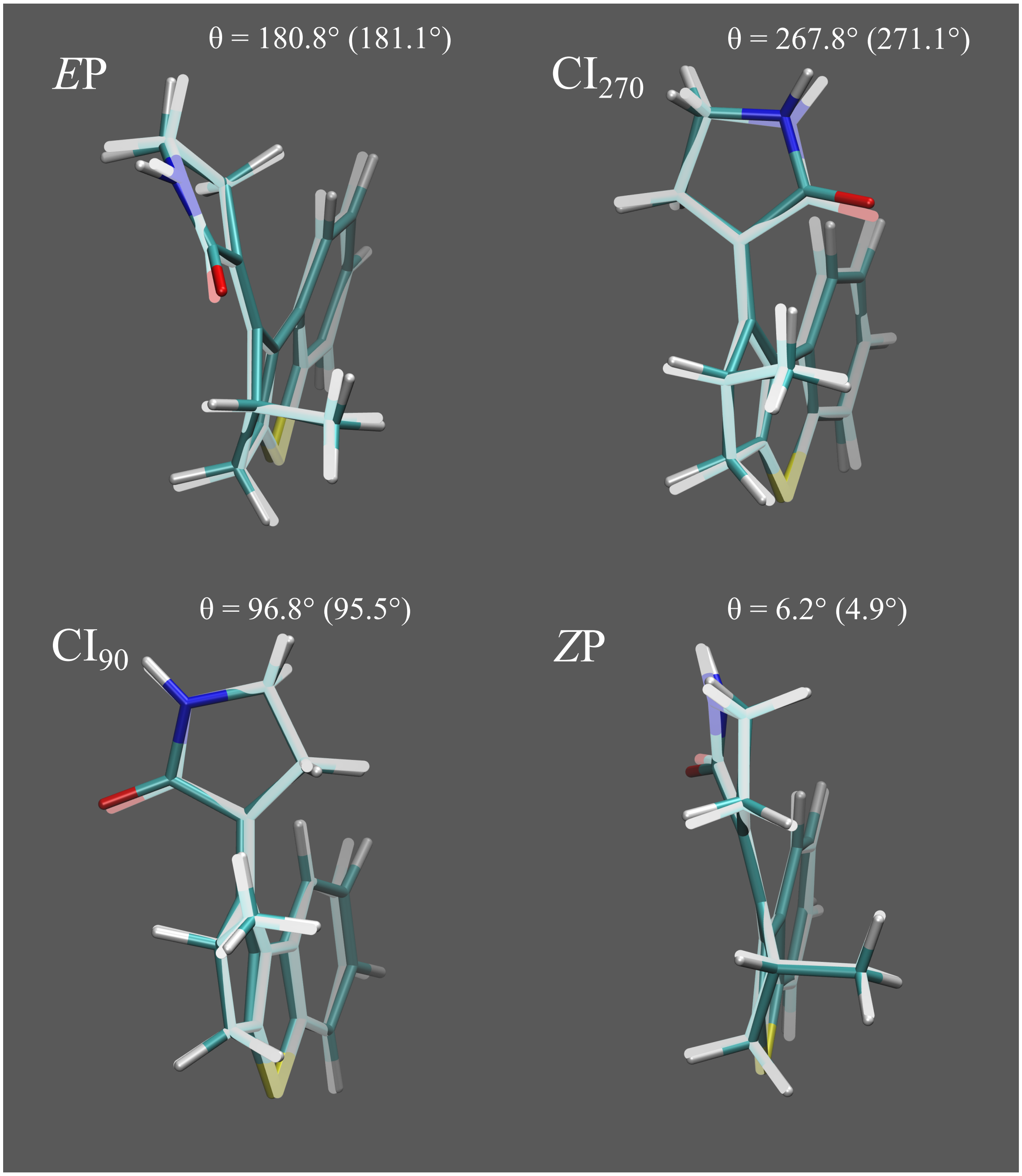

Supplementary Figure 21: Molecular geometries of the ground state minima and conical intersections of MTDP. For definition of the central dihedral angle θ, see Supplementary Figure 5. The structures obtained in the methanol environment are shown with solid colours and the gas phase geometries are shown with watery colours.

close to their gas-phase values.

Similar to the gas-phase calculations, the optimized stationary structures were used to set up approximate MEPs calculation on the S_1 and S_0 PESs. The gas-phase geometries

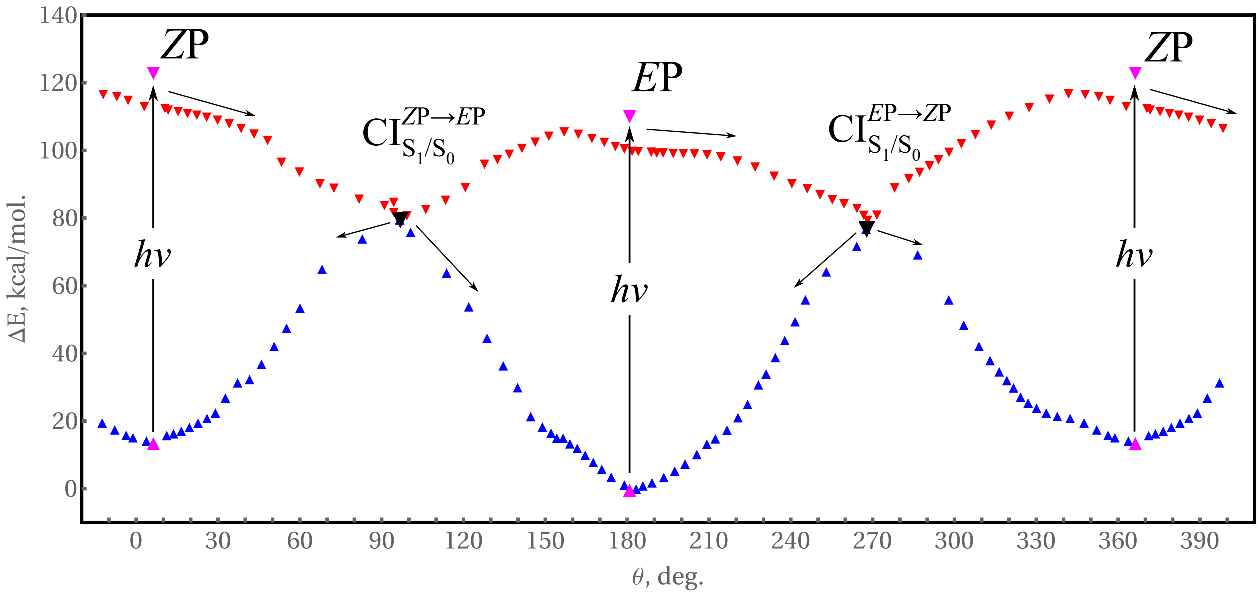

Supplementary Figure 22: The S_1 (red) and S_0 (blue) energy profiles along the MEPs optimized using the NEB method in the methanol environment modelled by QM/MM. The MEPs are shown in dependence on the central dihedral angle θ (deg.) defined in Supplementary Figure 5. The energies (kcal/mol) are given with respect to the *E*P ground state equilibrium configuration. The black triangles show the positions of the respective conical intersections. The magenta triangles show the positions of the *E*P and *Z*P equilibrium configurations and the respective FC points. Source data are provided as a Source Data file.

along the MEP were used as the starting geometries in QM/MM optimizations performed according to the NEB method[48] with fixed end points, as implemented in the DL-FIND code,[39]. During the optimization, the geometries of the MTDP snapshots ("images") along the MEP were updated by DL-FIND and their MM environment was optimized by TINKER. As mentioned above the positions and geometries of only 49 CH_3OH molecules have been optimized.

The obtained S_1 and S_0 MEPs in methanol environment are shown in Supplementary Figure 22, where the relative energies of the snapshots are shown as a function of the central dihedral angle θ. Again, the MEPs in methanol look similar to the gas-phase MEPs. Immediately upon the photoexcitation, shown in Supplementary Figure 22 by the vertical arrows, the relaxation along the BLA distortion occurs; this relaxation is the same

as in the gas-phase. In the vicinity of the EP conformer, there is a slope on the S_1 PES that drives the molecule in the CCW direction of torsion about the central double bond (axle).

At the $CI^{EP \to ZP}_{S_1/S_0}$ (CI_{270}) structure, the non-adiabatic transition to the S_0 state becomes possible. From this point on, the rotation of the rotor can proceed in both directions, CCW and CW, which are shown by short arrows. From the shape of the S_0 PES in the vicinity of $CI^{EP \to ZP}_{S_1/S_0}$ it can be expected that the probability of continuing rotation in the CCW sense may be lower than to move backward, to the original EP configuration. Indeed, the CI occurs somewhat earlier than the summit of the torsional barrier on the S_0 PES; this is also confirmed by the θ values reported in Supplementary Table 5. The somewhat earlier occurrence of the CI along the CCW torsion contrasts with the gas phase model; there the $CI^{EP \to ZP}_{S_1/S_0}$ structure occurred somewhat later and this indicated preference to continue torsion in the CCW direction. The earlier occurrence of the $CI^{EP \to ZP}_{S_1/S_0}$ structure is imposed by the methanol environment and may result in a lower EP $\to$ ZP isomerisation quantum yield, as more excited MTDP molecules in the EP configuration may return to the initial structure than to move forward to ZP.

As also discussed above, the most important characteristic of the EP $\to$ ZP segment of the S_0 MEP is the absence of a local minimum corresponding to the ZM structure. Similar to the gas-phase computations, only a shoulder is observed at the θ values typical for ZM. The same is seen in the ZP $\to$ EP segment of the S_0 MEP, where no stable local minimum corresponding to EM is seen. Hence, the mechanism of operation of the MTDP motor in methanol remains the same as in the gas-phase. The most evident difference between gas-phase and solution is, perhaps, in the probability of continuing rotation likely to be reduced in the solvent environment.

Obviously, the presented geometries of the stationary points and the MEPs have been obtained using only a single selected configuration of the methanol solvent "blob". A more precise answer could be obtained by sampling of a large number of possible solvent arrangements. This would, however, imply a prohibitively large amount of

computational work, which does not seem realistic, at the moment. Nevertheless, as shows the sampling of the ground state MD trajectory, the majority of solvent configurations occur near the selected solvent structure. Hence, we assume that the presented MEPs seem sufficiently representative for a qualitative (i.e. mechanistic) study. The validity of this assumption is somehow confirmed by the NAMD in solution investigations discussed below.

4.3.2 QM/MM NAMD Simulations of the *E*P Diastereoisomer

Three types of the QM/MM NAMD simulations were setup to confirm the mechanism of the *E*P photoisomerisation in methanol environment. First, two different settings were used to perform simulations using the fewest switches surface hopping molecular dynamics method (FSSH[62,63]) implemented in the MolCAS code.[64] In fact, these simulations were carried out by treating the QM solute (A) at the two-root SA CASSCF(12,11)/6-31G*level and (B) at the SSR(2,2)/BH&HLYP/6-31G* level. The former computations were carried out using the MolCAS/TINKER interface while the latter computations were carried out using our own GAMESS-US/TINKER interface to produce energies and gradients and then our in-house developed GAMESS-US/MolCAS interface to transfer the information to the MolCAS module propagating the trajectories (see the FSSH section below). Finally a third set of simulations were carried out using a different quantum-classical trajectory method, where the SSR(2,2) method was combined with DISH-XF as implemented in the PyUNI-xMD program (see the /PyUNI-xMD section below).

In the MolCAS FSSH simulations, the solvent molecules located within 4 Å from any atom of MTDP molecule and treated at the MM level (OPLS-AA force field) were allowed to move during QM/MM molecular dynamics while the rest of the solvent molecules were kept frozen. In order to produce the initial conditions necessary for running the excited state trajectories, the ground state QM/MM trajectories were

propagated following a single-root CASSCF gradient for case A and SSR(2,2) for case B for 200 fs and with a time step of 1 fs (41.3 au). The final snapshot was considered a point belonging to a room-temperature Boltzmann-like population in the electronic ground state. The corresponding atomic positions and velocities were used as initial conditions for the QM/MM trajectory propagation in the excited state.

FSSH trajectories simulations using MolCAS To elucidate the mechanistic details of photoisomerisation of MTDP in methanol solution we computed ten NAMD SSR(2,2) trajectories all starting in the Franck-Condon region of the S_1 PES of the *E*P diastereoisomer of MTPD. The ten corresponding initial conditions (geometries and velocities) were randomly selected from the above simulated room temperature Boltzmann-like distribution. The trajectories were propagated under imposing a qualitative 5.0 kcal/mol criterion for the S_1–S_0 gap at the surface hopping event. After the decay, the trajectories were continued according to the velocities and forces produced for the geometries immediately following the hops. During the simulation time S_1 to S_0 transitions were detected in seven trajectories where surface hopping occurred at 685, 526, 595, 205, 682, 703, and 621 fs. The total simulation times for these trajectories (including the S_1 and S_0 branch) were 1285, 1126, 1195, 805, 1282, 1303, and 1221 fs, respectively. Out of seven hopped trajectories (average hop time 542 fs) two exhibit clockwise (CW) torsional motion of the rotor with respect to the stator, while five displayed the expected counter-clockwise (CCW) motion. One of the two CW trajectories reverted to *E*P, while the other trajectory produced *Z*P. Of the five CCW trajectories, only one was productive and produced a metastable *Z*M that eventually produced *Z*P, while the other four revert to the *E*P isomer. We summarize the number of trajectories with observed outcomes in Supplementary Table 6 and their corresponding decay times in Supplementary Table 7.

In Supplementary Tables 8 and 9 we report the observed outcomes and decay times for

Supplementary Table 6: Number of trajectories with specific outcomes for SSR(2,2) simulations of EP in methanol solution

Torsional motion direction	unproductive	productive
CCW	4	1
CW	1	1
remained in S_1	3	0

Supplementary Table 7: Decay times (fs) of the SSR(2,2) trajectories of EP in methanol solution

Torsional motion direction	unproductive	productive
CCW	526, 205, 682, 703	595
CW	685	621
remained in S_1	–	–

the trajectories with the same initial geometries and velocities generated by GROMACS as in the SSR(2,2) simulations. However, the ground state QM/MM equilibration and the S_1 progression was simulated at the SA-CASSCF(12,11) level of theory. Notice that for the SA-CASSCF(12,11) trajectories the FSSH method has been used to detect the hopping point. Interestingly, this level of theory only produces CCW rotation motion (no CW motion) and one productive trajectory leading to ZM. The average hopping time of 722 fs is longer than that of the SSR(2,2) but still within a qualitatively acceptable value when compare with the observed time-scale of ca. 600 fs (see the main text).

Supplementary Table 8: Number of trajectories with specific outcomes for CASSCF(12,11) simulations in methanol solution

Torsional motion direction	unproductive	productive
CCW	5	1
CW	0	0
remained in S_1	4	0

Analysis of the only productive CCW trajectory detected by the SSR(2,2) method shows that the ZM to ZP transition is restrained by the solvent cage and requires a

Supplementary Table 9: Decay times (fs) of the CASSCF(12,11) trajectories in methanol solution

Torsional motion direction	unproductive	productive
CCW	729, 448, 585, 728, 757	1087
CW	–	–
remained in S_1	–	–

picosecond scale simulation time. The finding of only two productive trajectories over 20 computed trajectories at both levels of theory also suggests a relatively low quantum efficiency of the isomerisation process in solvent. Furthermore, the fact that only two out of 20 trajectories rotate in the CW rather than CCW direction, indicates a relatively high but not ideal unidirectional rotation. Notice that the productive CCW trajectory has been propagated up to 14458 fs, where the first 2458 fs correspond to the SSR(2,2) simulation and the remaining 12 ps are propagated at the BH&HLYP level to reduce the computational cost. Such a replacement is justified, because, for the ground electronic states of molecules well represented by a single electronic configuration, SSR and the conventional KS methods yield indistinguishable results.[8,9] In the top panel of Supplementary Figure 23 we demonstrate the change of the angle θ (the oxindole ring twisting) for the four selected trajectories representing four distinct observed dynamical behaviours (CW unproductive, CW productive, CCW unproductive and CCW productive). In the middle panel of the same figure we report the time progression of the charge residing on the oxindole ring along the only productive CCW trajectory (see above).

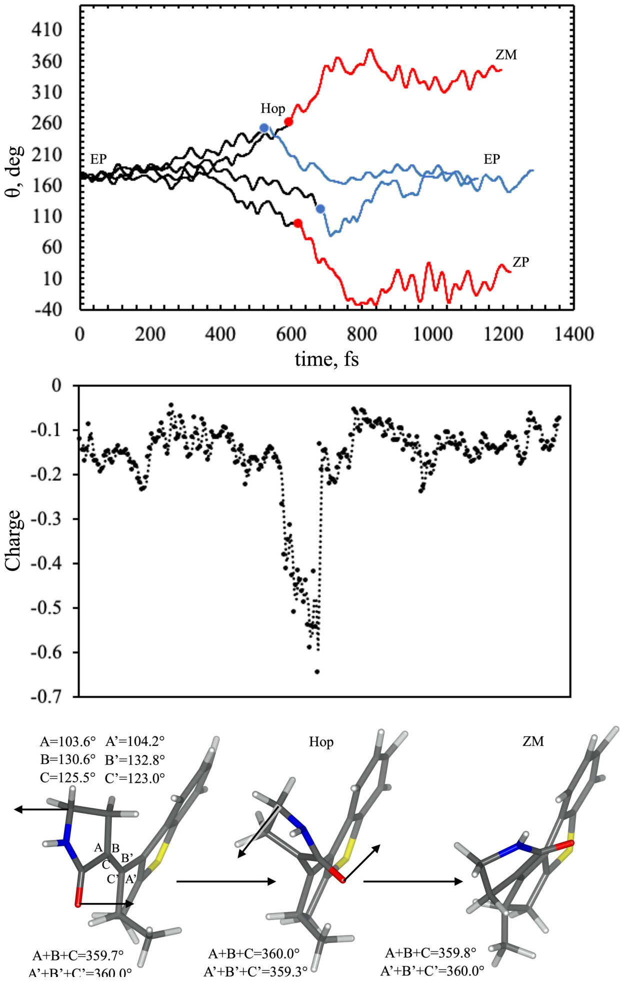

Supplementary Figure 23: Simulated photoisomerisation of the *E*P diastereoisomer of MTDP in methanol solution. Top panel. Progression of the dihedral angle θ along four trajectories representative of four different observed mechanisms. Progression along the S_1 PES (black lines) is connected with productive and unproductive progressions along the S_0 PES (red and blue lines, respectively) by the corresponding hop points (red and blue circles). Middle panel displays the change in the charge residing on the oxindole ring along the only productive CCW trajectory. The structures below the plot (bottom panel) display the geometrical evolution of the *E*P isomer, *Z*M isomer, and the connecting hopping point along the same productive CCW trajectory. The sum of the angles A, B, and C demonstrates the substantial planarity (sp^2 hybridization) of the oxindole ring carbon atom that connects the ring (rotor) to the bicyclic stator. Similarly, the sum of angles A′, B′, and C′ demonstrates the planarity of carbon atom on the other side of this double bond. Notice that the transition from *Z*M to *Z*P conformer occurs on longer (ps) timescale as documented in Supplementary Figure 24 (see also the attached movie).

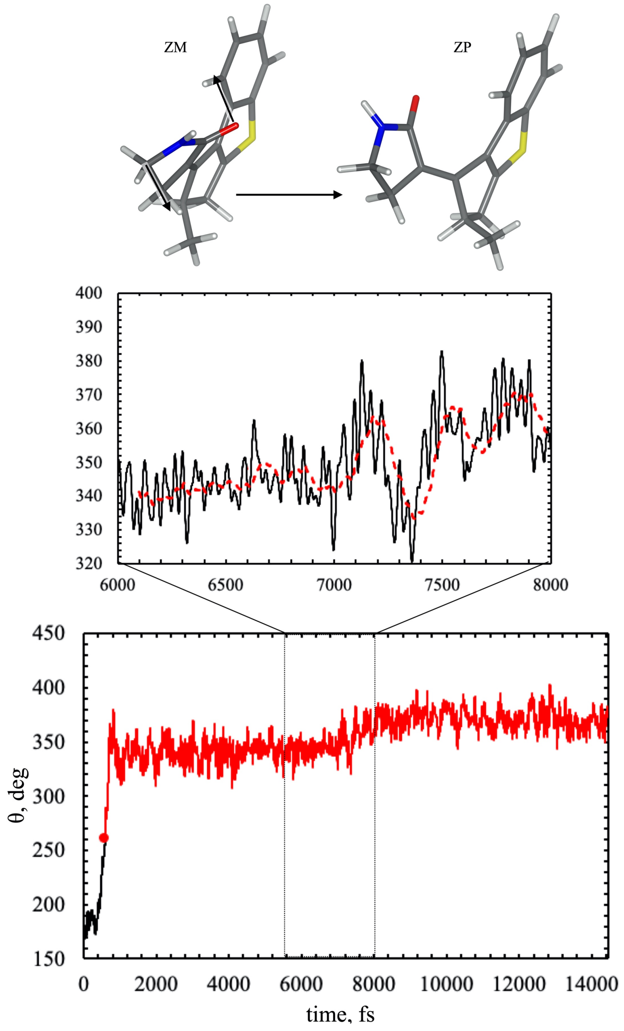

Supplementary Figure 24: Picosecond scale simulation of MTDP photoisomerisation in methanol solution as demonstrated through productive CCW trajectory. In the bottom panel the propagation along the S_1 PES is shown with black line, while the propagation along S_0 PES is shown with red line. The hop point is shown with red circle. Top panel demonstrates a magnified segment of the productive CCW trajectory between 6000 and 8000 fs at which the ZM to ZP transition occurs. Here the solid black line corresponds to propagation of the trajectory, while red dashed line represents the running average with the period of 100. The ZM and ZP structures shown above correspond to 6000 fs and 8000 fs timesteps of productive CCW trajectory. The total simulation time of productive CCW trajectory is 14458 fs, where first 2458 fs are simulated with SSR(2,2) and remaining 12 ps are executed with BH&HLYP.

Time Scale of the EP to ZP CCW motion in Solution using FSSH trajectories In order to improve our description of the time scale of the *E*P to *Z*P transition for the trajectories that undergo, exclusively, CCW isomerisation we selected a set of 40 initial conditions with prominent P-helicity (i.e. with an initial γ value ranging between 20 and 30 degrees. This is the torsional angle defined by the dihedral C4-C6-C8-C13 that is chosen to describe the change in helicity). The 40 corresponding quantum-classical trajectories where started on the S_1 PES and treated with the FSSH method and MolCAS (same as above). Initially the trajectories were executed for 850 fs at the SA-REKS(2,2) level of theory. In four case the propagation time was increased to 1100 fs due to the fact that hop from S_1 to S_0 only occurred at times longer than 790 fs (in the 790-850 fs range). After the hop each trajectory was propagated for, at least, an additional 200 fs using the same level of theory. At this point the subset of "productive" trajectories (i.e. the trajectories relaxing to the ZM photoproduct - not those leading back to EP) were further propagated on the S_0 PES using the fastest BH&HLYP method and, thus, allowing for the more time consuming (picosecond) description of the ZM to ZP transition. After ZP is reached due to a change in helicity, the trajectory was continued for 1 ps to ensure *Z*P stability. The total simulated times resulting from these calculations ranged from 1850 fs to 13500 fs.

Out of 40 simulated trajectories 32 underwent surface hopping. Of these, 32 trajectories only 8 were productive with surface hopping occurs at 396, 829, 799, 578, 343, 358, 385, and 370 fs, respectively. In Supplementary Figure 25 we report the change of θ (this is the dihedral angle C8-C6-C4-C2 describing the isomerization) during EP to ZM transition in all productive trajectories. Six productive trajectories displayed a ZM to ZP transition that occurred at 1469, 4858, 648, 3249, 883 and 1324 fs, respectively (see dashed vertical lines in in the Supplementary Figure 26). In the same figure we show also that θ does not significantly change during the EP to ZP transition. Two out of eight trajectories did not undergo ZM to ZP transition after the 13.5 ps of our simulation. Four movies of the fastest productive trajectory (648 fs), slowest productive trajectories, productive

trajectory where ZM to ZP transition did not occur within 13.5 ps and unproductive trajectory have been included in the Supplementary Data.

These results suggest that while the hop generally occurs within 1 ps for CCW trajectories (see above) the ZM to ZP transition may occur on a much wider time scale ranging (roughly) from 1 ps to over 15 ps.

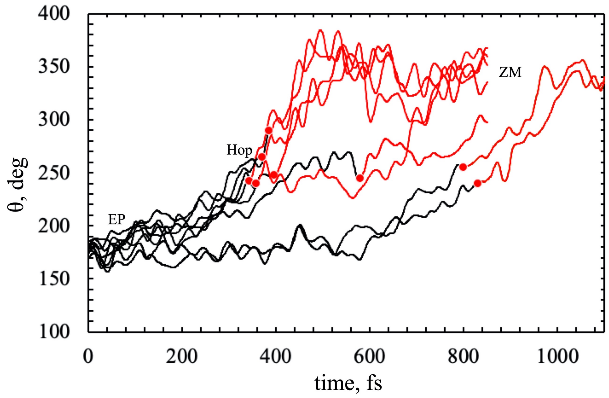

Supplementary Figure 25: Simulated photoisomerisation of MTDP in methanol solution based on 8 productive (i.e. productive. They reach the Z configuration) trajectories observed using 40 preselected initial conditions with prominent P-helicity character. The propagation along the S_1 PES (the black lines) is connected with the productive propagation along the S_0 PES (the red lines) by the corresponding hop points (the red circles).

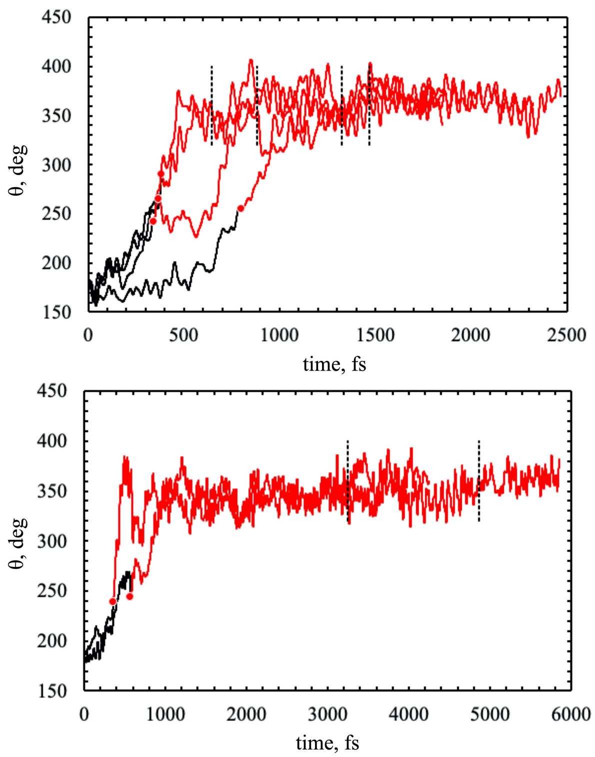

Supplementary Figure 26: Picosecond-scale simulation of MTDP photoisomerisation in methanol solution as demonstrated through 6 productive CCW trajectories successfully showing the ZM to ZP transition. Top panel shows 4 trajectories in which *E*P to *Z*P transition occurs within 2.5 ps. Bottom panel demonstrated 2 trajectories in which *E*P to *Z*P transition occurs within 6 ps. The propagation along the S_1 PES is shown with solid black lines, while the propagation along S_0 is shown with solid red lines. The hop points are marked with red circles. The point of trajectory at which *Z*P conformer is produced is identified with vertical dashed black lines.

SSR(2,2)/DISH-XF simulations The final set of simulations have been carried out using the SSR(2,2) method in connection with the DISH-XF molecular dynamics method implemented in the PyUNI-xMD package,[24] which has also been used in the gas-phase simulations. With this approach, twenty three NAMD trajectories have been completed using the decoherence induced surface hopping method based on the exact factorization of the electron-nuclear wavefunction (DISH-XF).[23,65,66] The reason why only twenty three trajectories have been done with this method is that it requires to calculate simultaneously the forces on the nuclei for both coupled states, S_1 and S_0. This is different from the usual FSSH approach, where the forces are calculated for the running state only, *i.e.*, S_1 or S_0. The number of MeOH molecules in the solvation shell of MTDP, which were active in the simulations, was increased to 100 molecules. The remaining 802 methanol molecules were kept frozen. The rest of the initial settings was the same as in the simulations with the FSSH method in MolCAS; see above.

Supplementary Table 10: Number of EP trajectories with specific outcomes for SSR(2,2)/DISH-XF simulations in methanol solution

Torsional motion direction	unproductive	productive
CCW	11	8
CW	1	0
remained in S_1	3	–

Supplementary Table 11: Decay times (fs) of the SSR(2,2)/DISH-XF trajectories of EP in methanol solution

Torsional motion direction	unproductive	productive
CCW	309,311,1058,241,261,<br>354,328,393,317,524,375	342,371,365,233,<br>463,278,559,498
CW	808	–
remained in S_1	–	–

The outcomes of the SSR(2,2)/DISH-XF simulations are summarized in Supplementary Tables 10 and 11. Eleven trajectories moved in the CCW direction and

fell back to the initial *E*P configuration, eight trajectories went in CCW and isomerised to *Z*P, and one trajectory went the wrong way, in CW, and fell back to *E*P. Three trajectories remained in the S_1 state until 1.5 ps, after which time they were terminated. Generally, this picture is consistent with what was obtained in the two simulations described in the section above.

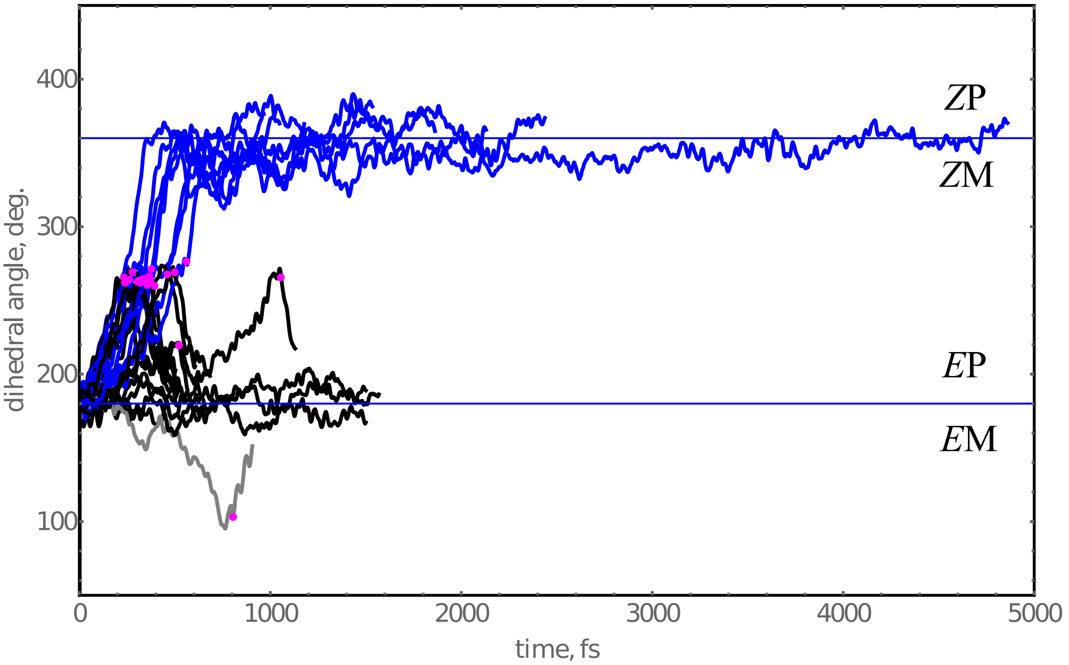

Supplementary Figure 27: Isomerisation of the *E*P isomer simulated using the SSR(2,2)-BH&HLYP/6-31G* method in connection with the DISH-XF method. Evolution of the dihedral angle θ is shown. The blue lines show the productive CCW trajectories, the black lines the unproductive CCW trajectories, and the grey line shows the unproductive trajectory isomerising in the CW direction of torsion. The magenta dots show the $S_1 \rightarrow S_0$ surface hops. The horizontal lines show the values of θ separating the *E*P/*E*M and ZP/ZP isomers.

Similar to the simulations with FSSH/MolCAS NAMD code, the eight productive trajectories first landed in the *Z*M configuration and, after a prolonged dynamics in the S_0 state finally reached the *Z*P configuration. The evolution of the dihedral angle θ along all the trajectories (productive, unproductive, CCW, CW) is shown in Supplementary Figure

27. The surface hops are shown by the magenta dots. As it is seen, after the $S_1 \rightarrow S_0$ transition, the eight productive trajectories (the blue lines) require upto 4 ps to reach the ZP configuration.

The dynamics parameters obtained in the SSR(2,2)/PyUNI-xMD simulations have been refined by bootstrapping[52] with 10^4 bootstrap replicas. In this way, the median values and the margins of error of the obtained median values can be reliably determined. Bootstrapping yields the following average values and margins of error: The S_1 lifetime $\tau_{S_1} = 562 \pm 87$fs, the isomerisation quantum yield $\Phi_{iso} = 0.35 \pm 0.10$, and the unidirectionality (the prevalence of the CCW over CW isomerisation direction) 0.95 ± 0.05. These parameters show that the solvent affects the most the quantum yield and to a lesser extent the unidirectionality of rotation. The main source of influence by the solvent is the "sticky" hydrogen bonds with the oxindole moiety, which often prevent or slow down motion in the direction that was preferred in the gas phase.

4.3.3 QM/MM NAMD Simulations of the *Z*P Diastereoisomer

To elucidate mechanistic details of photoisomerisation of the ZP conformer of MTDP in methanol solution we propagate ten SSR(2,2) trajectories on the S_1 PES starting in the Franck-Condon region. We continued the simulation on the S_0 PES for 600 fs using the geometries corresponding to the trajectory points where the S_1/S_0 gap becomes lower than 5.0 kcal/mol threshold. Upon hopping the trajectories were continued according to the velocities and forces of the geometry immediately following the hop. Imposing the 5.0 kcal/mol surface hopping criteria we observe S_1/S_0 transition in all ten trajectories where the surface hops occur at 233, 306, 269, 402, 204, 322, 359, 327, 351, and 694 fs. Thus, the total simulation times for these trajectories (S_1 and S_0 branch) are 833, 906, 869, 1002, 804, 922, 959, 927, 951, and 1294 fs, respectively.

Unlike the QM/MM trajectory simulations describing the behaviour of the *E*P diastereoisomer (see above), the corresponding *Z*P simulations were faster and they

were performed entirely at the SSR(2,2) level. This is because the *Z*P to *E*P transition occurs on a shorter time scale as compared to the *E*P to *Z*P transition; thus, allowing to finish the simulations within a reasonable time. All of the trajectories reach S_1/S_0 transition through counter-clockwise (CCW) torsional motion. After the S_1/S_0 transition, six trajectories were productive, while four other trajectories were unproductive. Out of six productive trajectories four ended in *E*P conformer, while other two ended in *E*M conformer. Eventually, an *E*M $\rightarrow$ *E*P transitions should occur on the S_0 PES; however, this would require much longer simulation times. Out of four unproductive trajectories three ended in *Z*M conformer, while other one reverted to *Z*P conformer. We summarize the number of trajectories with observed outcomes in Supplementary Table 12 and their corresponding decays time in Supplementary Table 13.

Supplementary Table 12: Number of trajectories with specific outcomes for SSR(2,2) simulations of ZP in methanol solution

Torsional motion direction	unproductive	productive
CCW	4	6
CW	0	0
remained in S_1	0	0

In Supplementary Figure 28 we demonstrate the change of θ for two trajectories that are representative of two observed reaction pathways, while showing the change in geometries and charge residing on the oxindole ring along the productive trajectory leading to *E*P conformer. For productive trajectory Supplementary Figure 28 describes the transition from *Z*P to *E*M, while Supplementary Figure 29 shows the change from *E*M to *E*P.

Supplementary Table 13: Decay times (fs) of simulated SSR(2,2) *ZP* trajectories in methanol solution

Torsional motion direction	unproductive	productive
CCW	269, 402, 351, 694	233, 306, 204, 322, 359, 327
CW	–	–
remained in S_1	–	–

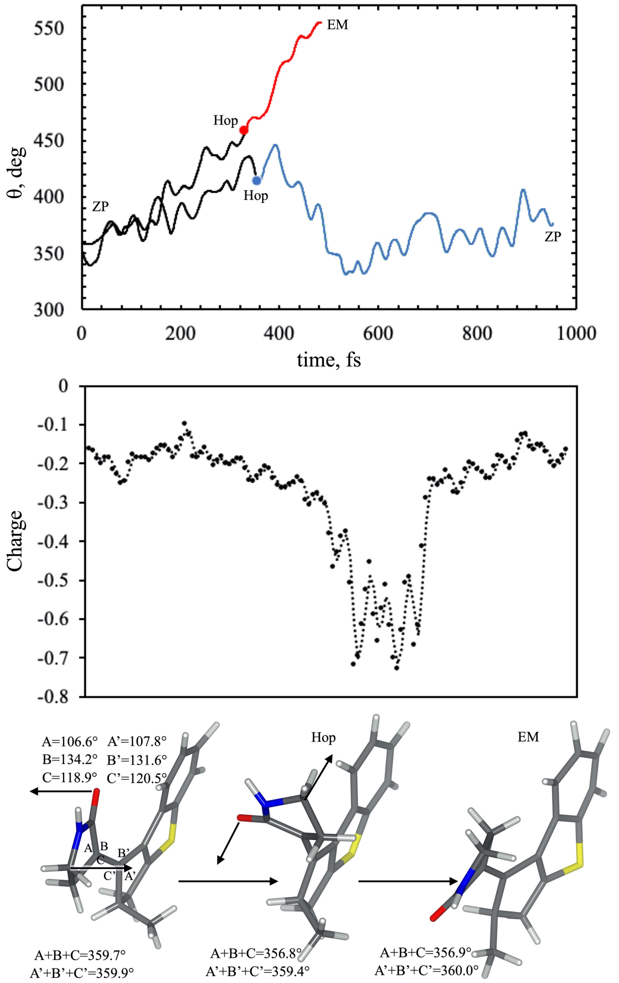

Supplementary Figure 28: Simulated photoisomerisation of *Z*P MTDP in methanol solution. Top panel demonstrates the change of θ for two trajectories that are representative of two observed reaction pathways. The propagation along the S_1 PES (the black lines) is connected with the productive and unproductive propagation along the S_0 PES (the red and blue lines, respectively) by the corresponding hop points (the red and blue circles). Middle panel demonstrates change in charge residing on the oxindole ring along the productive CCW trajectory. The structures below the plot display the geometries of *Z*P isomer, *E*M isomer, and the connecting hopping point along the productive CCW trajectory. Sum of angles A, B, and C characterize the planarity of oxindole ring sp^2 carbon atom that connects oxindole ring (rotor) to the stator, while sum of angles A′, B′, and C′ shows the planarity of carbon atom on the other side of this double bond.

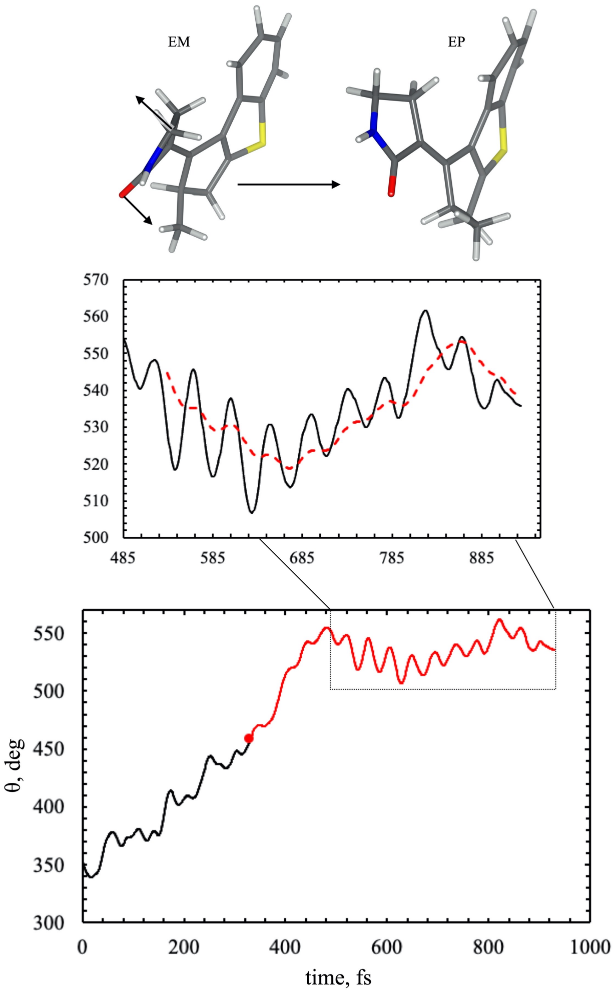

Supplementary Figure 29: The simulation of *Z*P MTDP photoisomerisation in methanol solution as demonstrated through productive trajectory. In the bottom panel the propagation along the S_1 PES is shown with black line, while the propagation along S_0 PES is shown with red line. The hop point is shown with red circle. Top panel demonstrates a magnified segment of the productive trajectory between 485 and 929 fs at which the *E*M to *E*P transition occurs. Here the solid black line corresponds to propagation of the trajectory, while red dashed line represents the running average with the period of 50 fs.

4.4 Evaluation of the total rotation quantum yield under steady state conditions

Let us assume that there is a mixture of the *E*P and *Z*P isomers with the initial fractions $[EP]_0 = a$ and $[ZP]_0 = 1 - a$, respectively. Let us further assume that the quantum yields of the two isomerisation steps (*E*P → *Z*P and *Z*P → *E*P) are ϕ_1 and ϕ_2, respectively, and that the thermal isomerisation steps are absent. The radiation wavelength is chosen such that the absorption cross-sections of both isomers are the same (the isosbestic point). Then, the photoreaction can be represented as in Chart 1. Here, it is also assumed that

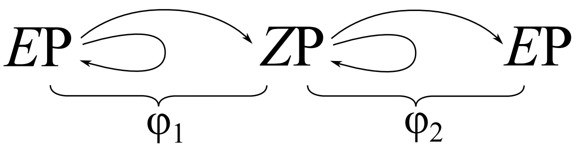

Chart 1: Schematic representation of the photocycle of 2-stroke motor. The forward arrows show the successful isomerisation, the returning arrows show the aborted isomerisations.

the light is shone on the reaction vessel in pulses (bursts) short enough to cause only a single isomerisation event, *E*P → *Z*P and *Z*P → *E*P. It takes two bursts to (possibly) cause one full rotation cycle of the motor; *i.e.*, *E*P → *Z*P → *E*P. In the following, we evaluate the quantum yield of the motor (*i.e.*, the average number of absorbed photons per full revolution) under the steady state conditions.

After the first burst of photons, the fractions of the isomers will change to

$$[EP]_1 = [EP]_0 - [EP]_0 \cdot \phi_1 + [ZP]_0 \cdot \phi_2 = a \cdot (1 - \phi_1) + (1 - a) \cdot \phi_2 \tag{9}$$

$$[ZP]_1 = [ZP]_0 - [ZP]_0 \cdot \phi_2 + [EP]_0 \cdot \phi_1 = 1 - a \cdot (1 - \phi_1) - (1 - a) \cdot \phi_2 \tag{10}$$

After the second burst of photons, the fractions will become

$$[EP]_2 = [EP]_1 \cdot (1 - \phi_1) + [ZP]_1 \cdot \phi_2 \tag{11}$$

$$[ZP]_2 = [ZP]_1 \cdot (1 - \phi_2) + [EP]_1 \cdot \phi_1 \tag{12}$$

Then, the total quantum yield Φ can be defined as, *e.g.*, the fraction of $[EP]$ that occurs after a successful isomerisation ZP $\rightarrow$ EP represented in Chart 1 by the second forward pointing arrow. This fraction is represented by the last term in the equation for $[EP]_2$; *i.e.*,

$$\Phi = [ZP]_1 \cdot \phi_2 \,. \tag{13}$$

The only unknown parameter in this equation is the fraction a, which can be determined by imposing the steady state condition, *i.e.*, $[EP]_0 = [EP]_2$, on the fractions of the isomers. Equating these fractions yields

$$a = a \cdot (1 - \phi_1) \cdot (1 - \phi_1) + (1 - a \cdot (1 - \phi_1) - (1 - a) \cdot \phi_2) \cdot \phi_2 \tag{14}$$

$$a \cdot (\phi_1 + \phi_2)(2 - \phi_1 - \phi_2) = \phi_2 \cdot (2 - \phi_1 - \phi_2) \tag{15}$$

from which follows the steady state value of a

$$a = \frac{\phi_2}{\phi_1 + \phi_2} \tag{16}$$

Substituting a to the equation for Φ yields

$$\Phi = \frac{\phi_1 \cdot \phi_2}{\phi_1 + \phi_2} \,. \tag{17}$$

For $a = 1$ (*i.e.*, no ZP isomer at the beginning of the photoreaction), eq. (13) yields $\Phi = \phi_1 \cdot \phi_2$, which, intuitively, seems the correct result of two consecutive photosteps in Chart 1. However, such an equation neglects the presence of the other isomer (ZP) and the steady state condition.

If one has $\phi_1 = \phi_2 = 0.25$, then the overall quantum yield from the steady fraction is

0.125. If, however, one assumes the initial fraction $a = 1$, then the overall quantum yield is 0.0625. For an ideal motor with $\phi_1 = \phi_2 = 1$, eq. (13) yields $\Phi = 0.5$, which is the maximum quantum efficiency of a 2-stroke light-driven molecular motor (consider that it takes absorption of at least two quanta to cause a full revolution).

5 Supplementary Note 5. Transient Absorption (TA) spectroscopy of *E*-2

5.1 Preliminaries: steady-state absorption spectra of the pure isomers

We prepare a methanol solution of a known mass of **2** in the "dark" (no light at wavelengths shorter than 400 nm is dark enough for compound **2**), and measure its absorption spectrum, before and after illumination with 300 nm light until a photostationary state (PSS) is reached. An isosbestic point at $\lambda = 319$ nm is carefully determined and useful for the correct relative scaling of the absorption spectra of solutions having different isomer content. The *E*:*Z* compositions of "dark" and "PSS" samples are also measured by H-NMR spectroscopy. The "dark" sample appears to be pure *E*, and the pure *Z* absorption spectrum is inferred from the appropriate linear combinations of both "dark" and "PSS" spectra, as illustrated in Supplementary Figure 30.

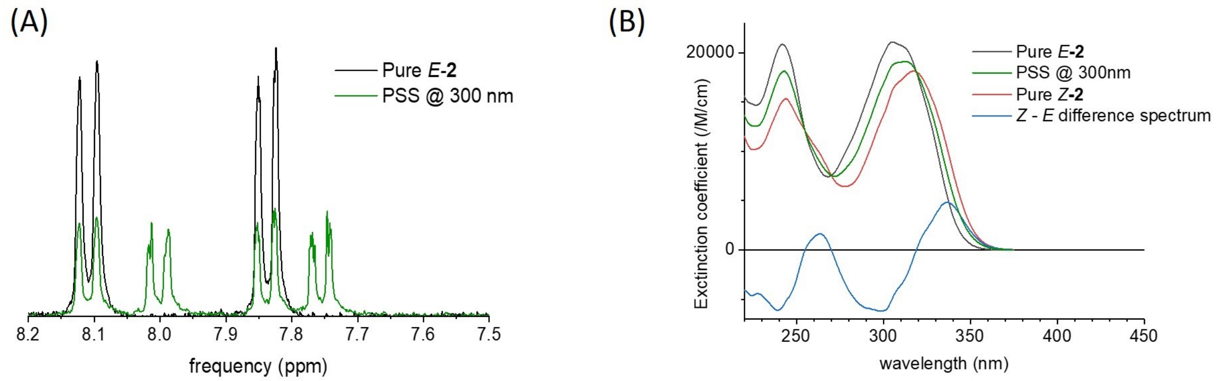

Supplementary Figure 30: (A) The H-NMR spectra of the dark and PSS samples reveal that the dark sample is pure *E* while the 300-nm PSS composition is 52% *E* and 48% *Z*. (B) Knowing the composition and spectra of the dark and PSS samples, we infer the pure *Z* absorption spectrum. The (*Z*- *E*) difference spectrum is also plotted, as we need it later for quantitative analysis of the TA data at long pump-probe delays. An isosbestic point is observed at $\lambda = 319 \pm 1$ nm.

5.2 TA Experimental set-up

A fresh methanol solution of *E*-**2** was prepared to an absorbance A=1.1/mm at its absorption maximum of λ_{max} =306 nm. The sample was investigated by femtosecond TA spectroscopy with a pump-probe set-up described elsewhere.[67] In short, we use the 800-nm, 40-fs pulse of a Ti:sapphire regenerative amplifier (Amplitude, operating at 5 kHz repetition rate) to pump a commercial, tunable optical parametric amplifier (TOPAS; Light Conversion) followed by fourth harmonic generation to produce a 290-nm pump pulse. A white-light continuum is generated with the fundamental 800 nm pulse in a 2 mm thick CaF_2 crystal, and used as a probe pulse offering an absorption detection window spanning from 310 nm to 750 nm. The polarization of the pump beam is set at the magic angle (54.7 degrees) with respect to that of the probe. The choice of a sub-300 nm pump pulse enables detecting the Ground State Bleach signal (GSB, observed from 310 to $\sim$ 350 nm) without any experimental noise due to pump light scattering, since the pump pulse lies outside this probe detection window. The time resolution is in the range 60 fs to 80 fs. Both pump and probe beams are focused and overlapped in the sample which is circulated in a 0.5-mm thick cuvette with a peristaltic pump.

5.3 TA Data analysis

The raw data are processed to subtract the solvent signal (recorded separately) and to correct for the chirp of the probe — *i.e.*, probe-wavelength-dependence of time zero (see the Supplementary Information of Ref. 67 for details). Quantitative data analysis is performed by singular value decomposition, followed by the global fitting of the dominating four singular transients. The fitting function is a multiexponential decay convoluted by a Gaussian function modelling the instrument response function (IRF). More precisely, the four multiexponential decay kinetics used to fit the four singular transients share the same time constants as global fitting parameters. The fits of the four

singular transients are then used to reconstruct the full 2D-map fit. The quality of this global fit is illustrated in Supplementary Figure 31 by overlapping the measured and fitted kinetics extracted from the corresponding 2D-maps at a selection of wavelengths. The result of the fit is disclosed by plotting the Decay-Associated-Spectra (DAS, see Figure 2-E of the main article), which reveal the wavelength dependence of the amplitudes associated with each decay component:

$$\Delta A^{fit}(\lambda, t) = \sum_{i=1}^{4} \mathrm{DAS}_i(\lambda) \exp(-t/\tau_i)$$

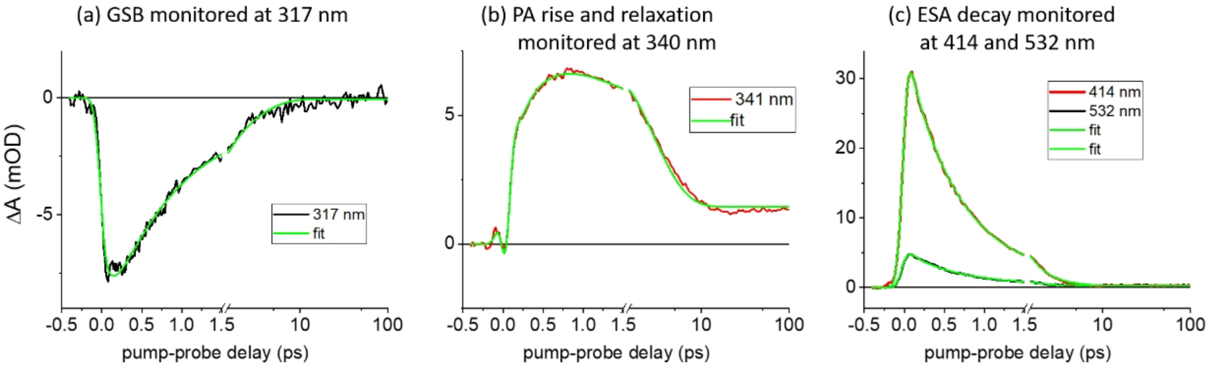

Supplementary Figure 31: Selection of kinetic traces and their fit as obtained from the global fit of the entire data set displayed in Figure 2 of the main article.

5.4 Photoisomerisation quantum yield of MTDP

5.4.1 Preliminary comment on the excitation probability

Consider a pump laser pulse (frequency ν_p, section S, energy per pulse $\mathcal{E}_p$) travelling through a solution of thickness l containing a solute at concentration c_0, with extinction coefficient $\varepsilon(\nu_p)$. The total absorbance of the solution at the pump wavelength is $A_p = \varepsilon(\nu_p)c_0 l$. The light energy $\Delta\mathcal{E}$ absorbed by the solution is the difference between the incident pulse energy and the pulse energy after propagating over l. According to the

Beer-Lambert law:

$$\Delta\mathcal{E} = \mathcal{E}_p \times (1 - 10^{-A_p}).$$

The corresponding number of absorbed photons $\Delta\mathcal{E}/(h\nu_p)$ is equal to the number of molecules excited. When dividing by the total number $c_0\, S\, l\; N_A$ of molecules in the illuminated volume (with N_A the Avogadro number), this gives the excitation probability x (averaged over the sample thickness l):

$$x = \frac{\Delta\mathcal{E}/(h\nu_p)}{c_0\, S\, l\; N_A} \quad \Rightarrow \quad \boxed{x = \frac{\varepsilon(\nu_p)}{N_A}\,\frac{\mathcal{E}_p}{S\, h\nu_p}\,\frac{1 - 10^{-A_p}}{A_p},}$$

where we have used $c_0\, l = A_p/\varepsilon(\nu_p)$.

Performing two TA experiments under "identical experimental conditions" means that $\mathcal{E}_p/(S\, h\nu_p)$ remains unchanged. Hence, one can infer the excitation probability x_E in the TA experiment on E-**2**, from the excitation probability x_t in the TA experiment performed "under the same conditions" on the actinometer t-AB according to:

$$x_E = x_t\,\frac{\varepsilon_E}{\varepsilon_t}\,\frac{1 - 10^{-A_E}}{A_E}\,\frac{A_t}{1 - 10^{-A_t}} \tag{18}$$

where $\varepsilon_E = 15000$ /M/cm (Supplementary Figure 30), $\varepsilon_t = 12683$ /M/cm[68] are the extinction coefficients of the compounds at $\lambda = 290$ nm, and A_E, A_t the samples absorbances at the same wavelength, measured just before the TA experiments.

We performed four distinct pairs of experiments on tAB and E-**2** every time on samples freshly prepared in the dark. For each pair of samples we computed the precise values of the x_E/x_t ratio according to Eq. 18. Since we always adjust the concentrations such that the sample absorbances (tAB or E-**2**) are in the range 0.5 to 0.6 (over 0.5 mm sample thickness), at their λ_{max}, the values for x_E/x_t were in the range of 1.0 to 1.2 for all experiments.

5.4.2 Using tAB as actinometer to determine η_E

For each TA experiment performed on tAB or E-**2**, a relation similar to Eq. (19) (same as Eq. (1) in main text) holds between the final TA spectra and the steady-state difference spectra:

$$\Delta A_E(\lambda, t = \infty) = x_E \times \eta_E \times [A_Z(\lambda) - A_E(\lambda)] \tag{19}$$

Supplementary Figure 32 illustrates the scalings between the "final" TA spectra and the steady-state difference spectra, which yield the products $x \times \eta$ in each case. Knowing the QY of t-AB at 290 nm $\eta_t = 0.15$,[69,70] we can infer the excitation probability x_t for t-AB under a given set of experimental conditions (pump pulse energy, diameter, etc... which are difficult to estimate accurately and need not be thanks to the use of an actinometer). Then, using Eq. (18) (see discussion above), we can infer the excitation probability x_E of E-**2** under the same conditions, hence infer η_E.

For the various pairs of TA experiments performed on tAB and E-**2**, the pump pulse energies were set in the range of 100 to 300 nJ per pulse, resulting in x_t values in the range 2 to 5% at maximum (depending on the pump pulse diameter in the sample). This low excitation probability (i.e. $\ll 1$) ensures the experiments were done in the linear regime of excitation. Given the achieved signal-to-noise in the TA experiment, and despite some variability among the four experimental sets in the actual spectral shapes of the final TA spectra relative to the steady-state difference spectra – Supplementary Figure 32 B and D are representative examples – the η_E values retrieved from the four pairs of experiments coincide within 0.02.

The main error in the η_E value is actually systematic and resides in the determination of (i) the extinction coefficient of E-**2**, (ii) the UV-Vis spectrum of the PSS at 300nm, (iii) the isosbestic point at 319 nm evaluated to within 1 nm, which all affect (iv) the amplitude and shape of the reference $A_Z(\lambda) - A_E(\lambda)$ difference spectrum (Supplementary Figure 30), hence the $x_E\, \eta_E$ scaling factors. We evaluate the overall error

bar on η_E to be 0.05, dominated by such systematic errors.

Taking into account the results of four pairs of experiments and our evaluation of the systematic errors, we conclude: $\eta_E = 0.25 \pm 0.05$.

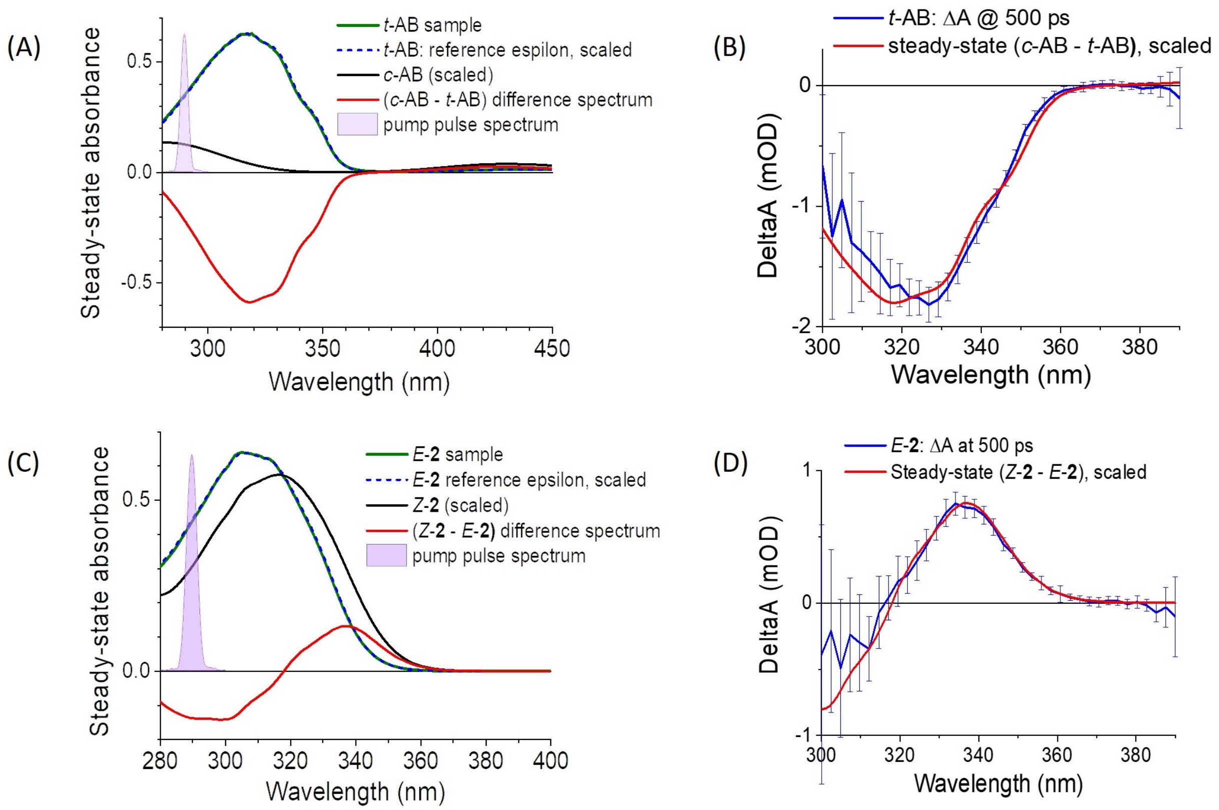

Supplementary Figure 32: Scaling of the steady-state isomer difference spectra to the corresponding final TA spectra. (A) and (C): the steady-state absorbance of the samples used in the TA experiments are displayed as green lines for *t*-AB and *E*-**2**, respectively. The spectra that would correspond to a 100% conversion to the other isomers are inferred from reference data (Ref. 68 for AB and Supplementary Figure 30 for *E*-**2**) and plotted in black. The corresponding isomer difference spectra are displayed in red. (B) and (D) the same isomer difference spectra (in red) are scaled to the final TA spectra (blue lines) recorded on each sample of *t*-AB and *E*-**2**, respectively. According to Eq. (19), the scaling factors are the $x\,\eta$ products characterizing each sample. The scaling factor determined in panel B yields $x_t = 2.1\%$ in this very experiment. Finally, using Eq.18 and the scaling factor determined in panel D, we conclude $\eta_E = 0.23$ for this very experimental run.

Supplementary References

(1) Landaluce, T. F.; London, G.; Pollard, M. M.; Rudolf, P.; Feringa, B. L. Rotary Molecular Motors: A Large Increase in Speed through a Small Change in Design. *J. Org. Chem.* **2010**, *75*, 5323–5325.

(2) Sheldrick, G. A short history of SHELX. *Acta Cryst., Sect. A* **2008**, *64*, 112–122.

(3) Sheldrick, G. Crystal structure refinement with SHELXL. *Acta Cryst., Sect. C Struct. Chem.* **2015**, *71*, 3–8.

(4) Filatov, M.; Shaik, S. A spin-restricted ensemble-referenced Kohn-Sham method and its application to diradicaloid situations. *Chem. Phys. Lett.* **1999**, *304*, 429–437.

(5) Moreira, I. d. P. R.; Costa, R.; Filatov, M.; Illas, F. Restricted ensemble-referenced Kohn-Sham versus broken symmetry approaches in density functional theory: Magnetic coupling in Cu binuclear complexes. *J. Chem. Theory Comput.* **2007**, *3*, 764–774.

(6) Kazaryan, A.; Heuver, J.; Filatov, M. Excitation Energies from Spin-Restricted Ensemble-Referenced Kohn-Sham Method: A State-Average Approach†. *J. Phys. Chem. A* **2008**, *112*, 12980–12988.

(7) Filatov, M. Assessment of density functional methods for obtaining geometries at conical intersections in organic molecules. *J. Chem. Theory Comput.* **2013**, *9*, 4526–4541.

(8) Filatov, M. Spin-restricted ensemble-referenced Kohn-Sham method: basic principles and application to strongly correlated ground and excited states of molecules. *WIREs Comput. Mol. Sci.* **2015**, *5*, 146–167.

(9) Filatov, M. In *Density-functional methods for excited states*; Ferré, N., Filatov, M., Huix-Rotllant, M., Eds.; Top. Curr. Chem.; Springer: Heidelberg, 2016; Vol. 368; pp 97–124.

(10) Valone, S. M. A one-to-one mapping between one-particle densities and some n-particle ensembles. *J. Chem. Phys.* **1980**, *73*, 4653–4655.

(11) Lieb, E. H. Density functionals for Coulomb systems. *Int. J. Quantum Chem.* **1983**, *24*, 243–277.

(12) Perdew, J. P.; Parr, R. G.; Levy, M.; Balduz Jr., J. L. Density-Functional Theory for Fractional Particle Number: Derivative Discontinuities of the Energy. *Phys. Rev. Lett.* **1982**, *49*, 1691–1694.

(13) Englisch, H.; Englisch, R. Hohenberg-Kohn Theorem and Non-V-Representable Densities. *Physica* **1983**, *A121*, 253–268.

(14) Englisch, H.; Englisch, R. Exact Density Functionals for Ground-State Energies. I. General Results. *Phys. Stat. Sol. (b)* **1984**, *123*, 711–721.

(15) Englisch, H.; Englisch, R. Exact Density Functionals for Ground-State Energies II. Details and Remarks. *Phys. Stat. Sol. (b)* **1984**, *124*, 373–379.

(16) Gross, E. K. U.; Oliveira, L. N.; Kohn, W. Rayleigh-Ritz variational principle for ensembles of fractionally occupied states. *Phys. Rev. A* **1988**, *37*, 2805–2808.

(17) Gross, E. K. U.; Oliveira, L. N.; Kohn, W. Density-functional theory for ensembles of fractionally occupied states. I. Basic formalism. *Phys. Rev. A* **1988**, *37*, 2809–2820.

(18) Oliveira, L. N.; Gross, E. K. U.; Kohn, W. Density-functional theory for ensembles of fractionally occupied states. II. Application to the He atom. *Phys. Rev. A* **1988**, *37*, 2821–2833.

(19) Oliveira, L. N.; Gross, E. K. U.; Kohn, W. Ensemble-Density Functional Theory. *Int. J. Quantum Chem.: Quantum Chem. Symp.* **1990**, *24*, 707–716.

(20) Filatov, M.; Liu, F.; Martínez, T. J. Analytical derivatives of the individual state energies in ensemble density functional theory method. I. General formalism. *J. Chem. Phys.* **2017**, *147*, 034113.

(21) Filatov, M.; Lee, S.; Choi, C. H. Computation of Molecular Ionization Energies Using an Ensemble Density Functional Theory Method. *J. Chem. Theory Comput.* **2020**, *16*, 4489–4504.

(22) Filatov, M.; Lee, S.; Choi, C. H. Description of Sudden Polarization in the Excited Electronic States with an Ensemble Density Functional Theory Method. *J. Chem. Theory Comput.* **2021**, *17*, 5123–5139.

(23) Ha, J.-K.; Lee, I. S.; Min, S. K. Surface Hopping Dynamics beyond Nonadiabatic Couplings for Quantum Coherence. *J. Phys. Chem. Lett.* **2018**, *9*, 1097–1104.

(24) Lee, I. S.; Ha, J.-K.; Han, D.; Kim, T. I.; Moon, S. W.; Min, S. K. PyUNIxMD: A Python-based excited state molecular dynamics package. *J. Comp. Chem.* **2021**, *42*, 1755–1766.

(25) Hunter, G. Conditional probability amplitudes in wave mechanics. *Int. J. Quantum Chem.* **1975**, *9*, 237–242.

(26) Abedi, A.; Maitra, N. T.; Gross, E. K. U. Exact factorization of the time-dependent electron-nuclear wave function. *Phys. Rev. Lett.* **2010**, *105*, 123002.

(27) Abedi, A.; Maitra, N. T.; Gross, E. K. U. Correlated electron-nuclear dynamics: Exact factorization of the molecular wave-function. *J. Chem. Phys.* **2012**, *137*, 22A530.

(28) Abedi, A.; Agostini, F.; Suzuki, Y.; Gross, E. K. U. Dynamical steps that bridge piecewise adiabatic shapes in the exact time-dependent potential energy surface. *Phys. Rev. Lett.* **2013**, *110*, 263001.

(29) Agostini, F.; Abedi, A.; Suzuki, Y.; Min, S. K.; Maitra, N. T.; Gross, E. K. U. The exact

electronic back-reaction on classical nuclei in non-adiabatic charge transfer. *J. Chem. Phys.* **2015**, *142*, 084303.

(30) Tully, J. C. Molecular dynamics with electronic transitions. *J. Chem. Phys.* **1990**, *93*, 1061.

(31) Granucci, G.; Persico, M.; Zoccante, A. Including quantum decoherence in surface hopping. *J. Chem. Phys.* **2010**, *133*, 134111.

(32) Hammes-Schiffer, S.; Tully, J. C. Proton transfer in solution: Molecular dynamics with quantum transitions. *J. Chem. Phys.* **1994**, *101*, 4657–4667.

(33) Schmidt, M. W.; Baldridge, K. K.; Boatz, J. A.; Elbert, S. T.; Gordon, M. S.; Jensen, J. J.; Koseki, S.; Matsunaga, N.; Nguyen, K. A.; Su, S.; Windus, T. L.; Dupuis, M.; Montgomery, J. A. *J. Comput. Chem.* **1993**, *14*, 1347–1363.

(34) Gordon, M.; Schmidt, M. In *Theory and Applications of Computational Chemistry, the first forty years*; Dykstra, C. E., Frenking, G., Kim, K. S., Scuseria, G. E., Eds.; Elsevier: Amsterdam, 2005; pp 1167–1189.

(35) Krishnan, R.; Binkley, J. S.; Seeger, R.; Pople, J. A. Self-consistent molecular orbital methods. XX. A basis set for correlated wave functions. *J. Chem. Phys.* **1980**, *72*, 650–654.

(36) Becke, A. D. Density-Functional Exchange-Energy Approximation with Correct Asymptotic Behavior. *Phys. Rev. A* **1988**, *38*, 3098–3100.

(37) Lee, C.; Yang, W.; Parr, R. G. Development of the Colle-Salvetti Correlation-Energy Formula into a Functional of the Electron Density. *Phys. Rev. B* **1988**, *37*, 785–789.

(38) Becke, A. D. A New Mixing of Hartree-Fock and Local Density-Functional Theories. *J. Chem. Phys.* **1993**, *98*, 1372–1377.

(39) Kästner, J.; Carr, J. M.; Keal, T. W.; Thiel, W.; Wander, A.; Sherwood, P. DL-FIND: An Open-Source Geometry Optimizer for Atomistic Simulations. *J. Phys. Chem. A* **2009**, *113*, 11856–11865.

(40) Levine, B.; Coe, J. D.; Martínez, T. J. Optimizing conical intersections without derivative coupling vectors: application to multistate multireference second-order perturbation theory (MS-CASPT2). *J. Phys. Chem. B* **2008**, *112*, 405–413.

(41) Oppenheim, I.; Ross, J. Temperature Dependence of Distribution Functions in Quantum Statistical Mechanics. *Phys. Rev.* **1957**, *107*, 28–32.

(42) Davies, R. W.; Davies, K. T. R. On the Wigner Distribution Function for an Oscillator. *Ann. Phys.* **1975**, *89*, 261–273.

(43) Filatov, M.; Paolino, M.; Min, S. K.; Choi, C. H. Design and photoisomerization dynamics of a new family of synthetic 2-stroke light driven molecular rotary motors. *Chem. Commun.* **2019**, *55*, 5247–5250.

(44) Filatov, M.; Olivucci, M. Designing conical intersections for light-driven single molecule rotary motors: From precessional to axial motion. *J. Org. Chem.* **2014**, *79*, 3587–3600.

(45) Huix-Rotllant, M.; Filatov, M.; Gozem, S.; Schapiro, I.; Olivucci, M.; Ferré, N. Assessment of Density Functional Theory for Describing the Correlation Effects on the Ground and Excited State Potential Energy Surfaces of a Retinal Chromophore Model. *J. Chem. Theory Comput.* **2013**, *9*, 3917–3932.

(46) Gozem, S.; Melaccio, F.; Valentini, A.; Filatov, M.; Huix-Rotllant, M.; Ferré, N.; Frutos, L. M.; Angeli, C.; Krylov, A. I.; Granovsky, A. A.; Lindh, R.; Olivucci, M. Shape of Multireference, Equation-of-Motion Coupled-Cluster, and Density Functional Theory Potential Energy Surfaces at a Conical Intersection. *J. Chem. Theory Comput.* **2014**, *10*, 3074–3084.

(47) Wilson, C. C. *Single Crystal Neutron Diffraction from Molecular Materials*; World Scientific Publishing Corporation, Singapore, 2000.

(48) Jónsson, H.; Mills, G.; Jacobsen, K. W. In *Classical and Quantum Dynamics in Condensed Phase Simulations*; Berne, B. J., Ciccotti, G., Coker, D. F., Eds.; World Scientific: Singapore, 1998; Chapter 16, pp 385–404.

(49) Cremer, D.; Pople, J. A. General definition of ring puckering coordinates. *J. Am. Chem. Soc.* **1975**, *97*, 1354–1358.

(50) Koumura, N.; Zijlstra, R. W. J.; van Delden, R. A.; Harada, N.; Feringa, B. L. Light-Driven Monodirectional Molecular Rotor. *Nature* **1999**, *401*, 152–155.

(51) Lan, Z.; Lu, Y.; Weingart, O.; Thiel, W. Nonadiabatic Decay Dynamics of a Benzylidene Malononitrile. *J. Phys. Chem. A* **2012**, *116*, 1510–1518.

(52) Nangia, S.; Jasper, A. W.; Miller, T. F.; Truhlar, D. G. Army ants algorithm for rare event sampling of delocalized nonadiabatic transitions by trajectory surface hopping and the estimation of sampling errors by the bootstrap method. *J. Chem. Phys.* **2004**, *120*, 3586–3597.

(53) Head-Gordon, M. .; Pople, J. A. .; Frisch, M. J. MP2 Energy Evaluation by Direct Methods. *Chem. Phys. Lett.* **1988**, *153*, 503–506.

(54) Frisch, M. J.; Head-Gordon, M.; Pople, J. A. A Direct MP2 Gradient Method. *Chem. Phys. Lett.* **1990**, *166*, 275–280.

(55) Tomasi, J.; Mennucci, B.; Cammi, R. Quantum Mechanical Continuum Solvation Models. *Chem. Rev.* **2005**, *105*, 2999–3094.

(56) Frisch, M. J. et al. Gaussian 09 Revision C.01. 2016; Gaussian Inc. Wallingford CT.

(57) Caleman, C.; van Maaren, P. J.; Hong, M.; Hub, J. S.; Costa, L. T.; van der Spoel, D. Force Field Benchmark of Organic Liquids: Density, Enthalpy of Vaporization,

Heat Capacities, Surface Tension, Isothermal Compressibility, Volumetric Expansion Coefficient, and Dielectric Constant. *J. Chem. Theory Comput.* **2012**, *8*, 61–74.

(58) Pronk, S.; Páll, S.; Schulz, R.; Larsson, P.; Bjelkmar, P.; Apostolov, R.; Shirts, M. R.; Smith, J. C.; Kasson, P. M.; van der Spoel, D.; Hess, B.; Lindahl, E. GROMACS 4.5: a high-throughput and highly parallel open source molecular simulation toolkit. *Bioinformatics* **2013**, *29*, 845–854.

(59) Kawai, S.; Canova, F. F.; Glatzel, T.; Foster, A. S.; Meyer, E. Atomic-scale dissipation processes in dynamic force spectroscopy. *Phys. Rev. B* **2011**, *84*, 115415.

(60) Aldaz, C. R.; Martínez, T. J.; Zimmerman, P. M. The Mechanics of the Bicycle Pedal Photoisomerization in Crystalline cis,cis-1,4-Diphenyl-1,3-butadiene. *J. Phys. Chem. A* **2020**, *124*, 8897–8906.

(61) Ponder, F. M., J. W .; Richards TINKER Molecular Modeling Package. *J. Comput. Chem.* **1987**, *8*, 1016–1024.

(62) Tully, J. C. Molecular dynamics with electronic transitions. *J. Chem. Phys.* **1990**, *93*, 1061.

(63) Granucci, G.; Persico, M. Critical appraisal of the fewest switches algorithm for surface hopping. *J. Chem. Phys.* **2007**, *126*, 134114.

(64) Aquilante, F. et al. Molcas 8: New capabilities for multiconfigurational quantum chemical calculations across the periodic table. *J. Comput. Chem.* **2016**, *37*, 506–541.

(65) Min, S. K.; Agostini, F.; Tavernelli, I.; Gross, E. K. U. Ab Initio Nonadiabatic Dynamics with Coupled Trajectories: A Rigorous Approach to Quantum (De)Coherence. *J. Phys. Chem. Lett.* **2017**, *8*, 3048–3055.

(66) Agostini, F.; Min, S. K.; Abedi, A.; Gross, E. K. U. Quantum-Classical Nonadiabatic

Dynamics: Coupled- vs Independent-Trajectory Methods. *J. Chem. Theory Comput.* **2016**, *12*, 2127–2143.

(67) Briand, J.; Bräm, O.; Réhault, J.; Léonard, J.; Cannizzo, A.; Chergui, M.; Zanirato, V.; Olivucci, M.; Helbing, J.; Haacke, S. Coherent ultrafast torsional motion and isomerization of a biomimetic dipolar photoswitch. *Phys. Chem. Chem. Phys.* **2010**, *12*, 3178–3187.

(68) Vetráková, Ĺ.; Ladányi, V.; Al Anshori, J.; Dvořák, P.; Wirz, J.; Heger, D. The absorption spectrum of cis-azobenzene. *Photochem. Photobiol. Sci.* **2017**, *16*, 1749–1756.

(69) Ladányi, V.; Dvořák, P.; Al Anshori, J.; Vetráková, Ĺ.; Wirz, J.; Heger, D. Azobenzene photoisomerization quantum yields in methanol redetermined. *Photochem. Photobiol. Sci.* **2017**, *16*, 1757–1761.

(70) Quick, M.; Dobryakov, A. L.; Gerecke, M.; Richter, C.; Berndt, F.; Ioffe, I. N.; Granovsky, A. A.; Mahrwald, R.; Ernsting, N. P.; Kovalenko, S. A. Photoisomerization Dynamics and Pathways of trans- and cis-Azobenzene in Solution from Broadband Femtosecond Spectroscopies and Calculations. *J. Phys. Chem. B* **2014**, *118*, 8756–8771.
