# Supplementary material for: Towards the engineering of a photon-only two-stroke rotary molecular motor
Source: Nat Commun. 2022 Oct 28;13:6433. doi: 10.1038/s41467-022-33695-x (PMC9616945; doi:10.1038/s41467-022-33695-x)
Supplement: Supplementary file 2 — Description of Additional Supplementary Files [file 41467_2022_33695_MOESM2_ESM.docx]

Source Data: A zipped archive with: 1) xlsx spreadsheets with the source data for Figures 4A and 4B of the main article, 2) Supplementary Figure 21 of the Supplementary Information, 3) Figure 6A of the main article, 4) two folders with the source data for Figures 4C and 4D of the main article.

Supplementary Movie 1: Animation of gas phase productive EP -> ZP trajectories

Supplementary Movie 2: Animation of gas phase productive ZP -> EP trajectories

Supplementary Movie 3: Animation of the full working cycle of MTDP

Supplementary Movie 4: Animation of a productive EP -> ZP trajectory in methanol solution (from SSR/DISH-XF simulations)

Supplementary Movie 5: Animation of an unproductive EP -> ZP trajectory in methanol solution (from SSR/DISH-XF simulations)

Supplementary Movie 6: Animation of a productive EP -> ZP trajectory in methanol solution (from SSR/FSSH simulations). The trajectory is characterised by a fast isomerisation time.

Supplementary Movie 7: Animation of a productive EP -> ZP trajectory in methanol solution (from SSR/FSSH simulations). The trajectory is characterised by a slow isomerisation time.

Supplementary Movie 8: Animation of a productive EP -> ZP trajectory in methanol solution (from SSR/FSSH simulations), where no ZM -> ZP transition occurs after 13.5ps.

Supplementary Movie 9: Animation of an unproductive EP -> ZP trajectory in methanol solution (from SSR/FSSH simulations).

Supplementary Movie 10: Animation of a productive ZP -> EP trajectory in methanol solution (from SSR/FSSH simulations)
