# Supplementary figures and images for: Towards the engineering of a photon-only two-stroke rotary molecular motor
Source: Nat Commun. 2022 Oct 28;13:6433. doi: 10.1038/s41467-022-33695-x (PMC9616945; doi:10.1038/s41467-022-33695-x)

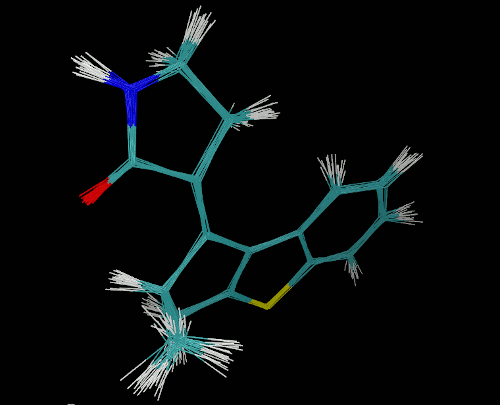

Supplement: Supplementary file 3 — Supplementary Movie 1 [file 41467_2022_33695_MOESM3_ESM.gif]

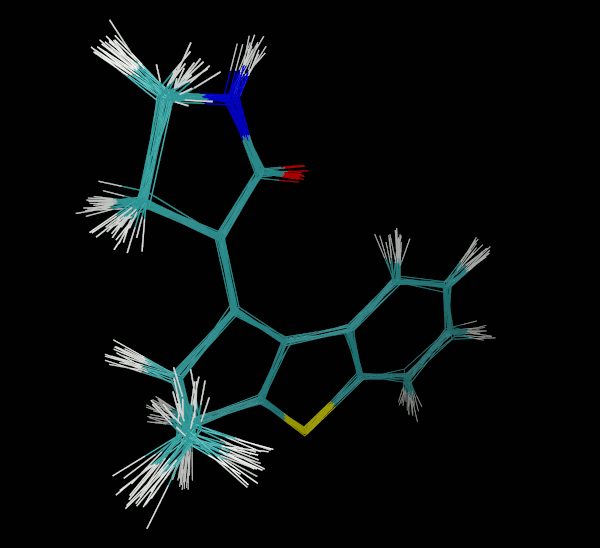

Supplement: Supplementary file 4 — Supplementary Movie 2 [file 41467_2022_33695_MOESM4_ESM.gif]

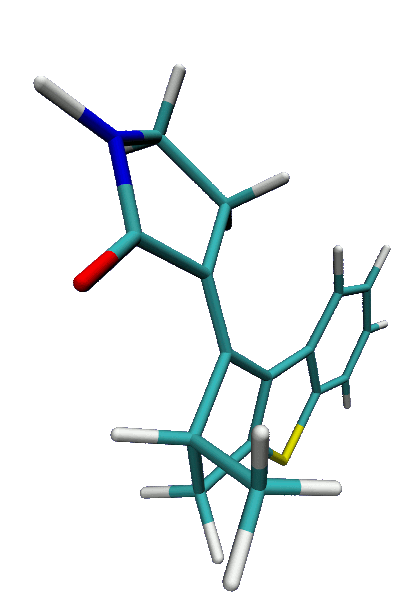

Supplement: Supplementary file 5 — Supplementary Movie 3 [file 41467_2022_33695_MOESM5_ESM.gif]
